# Equal accuracy for Andrew and Abubakar—detecting and mitigating bias in name-ethnicity classification algorithms

**DOI:** 10.1007/s00146-022-01619-4

**Published:** 2023-02-09

**Authors:** Lena Hafner, Theodor Peter Peifer, Franziska Sofia Hafner

**Affiliations:** 1 Department of Politics and International Studies, University of Cambridge, Cambridge, UK; 2 Munich Center for Digital Sciences and AI, Hochschule München, Munich, Germany; 3 Department of Social and Public Policy and Computer Science, University of Glasgow, Glasgow, UK

**Keywords:** Name-ethnicity-classification, Machine learning, Artificial intelligence, AI fairness audit, Algorithmic bias, Critical tech, Ethnic inequalities

## Abstract

Uncovering the world’s ethnic inequalities is hampered by a lack of ethnicity-annotated datasets. Name-ethnicity classifiers (NECs) can help, as they are able to infer people’s ethnicities from their names. However, since the latest generation of NECs rely on machine learning and artificial intelligence (AI), they may suffer from the same racist and sexist biases found in many AIs. Therefore, this paper offers an algorithmic fairness audit of three NECs. It finds that the UK-Census-trained *EthnicityEstimator* displays large accuracy biases with regards to ethnicity, but relatively less among gender and age groups. In contrast, the Twitter-trained *NamePrism* and the Wikipedia-trained *Ethnicolr* are more balanced among ethnicity, but less among gender and age. We relate these biases to global power structures manifested in naming conventions and NECs’ input distribution of names. To improve on the uncovered biases, we program a novel NEC, *N2E*, using fairness-aware AI techniques. We make *N2E* freely available at www.name-to-ethnicity.com.

**Supplementary Information:**

The online version contains supplementary material available at 10.1007/s00146-022-01619-4.

## Introduction

**Table:** 

Katrin Müller	from ————————
José Maria Garcia Lopez	from ————————
Yǔtóng Zhang	from ————————
David Smith	from ————————

Even without knowing these individuals, most people accurately deduce the origin of their names. They can do this because through their lived experience they have internalised the cultural–ethnic–linguistic conventions that underlie naming practices from around the world. Nevertheless, letting people perform this task might have two drawbacks. First, computer programmes will beat them at it. In fact, so-called name-ethnicity classifiers (NECs) are gaining traction in business, research and policy as they enable the analysis of large datasets of personal names for their ethnic or national composition. Second, humans are likely to be biased. The more restricted a name is to underprivileged backgrounds the less likely it is that one might learn to classify the name by encountering it in one’s personal environment or in the media. However, just like humans, algorithms are vulnerable to bias. A growing body of algorithmic fairness research has demonstrated how AI—from facial recognition to word embeddings—is ‘sexist and racist’ (Zou and Schiebinger 2018, p. 324). This literature has so far not scrutinised NECs. The present paper fills this gap by auditing NECs with regards to their race, gender and age biases. Its goal is, first, to empower researchers to use NECs more consciously, and, second, to highlight ways for de-biasing existing NECs. To this end, the paper first gives an introduction to NECs in connection to the algorithmic fairness literature. Second, a methodology section details the procedures and metrics. Furthermore, the section describes how we train our own NEC to minimise bias. Third, an analysis section compares the biases of three NECs. It finds that whereas the UK-Census-trained EthnicityEstimator has large variations in sensitivities amongst its ethnic categories, it is relatively balanced with regards to gender and age. In contrast, NamePrism and Ethnicolr, trained on Twitter and Wikipedia, respectively, are more balanced with regards to ethnicity, but introduce further bias among gender and age groups. To varying degrees, these biases are found to originate from naming conventions and the distribution of names in NECs’ training-data. Subsequently, the paper shows how much we were able to reduce these biases through fairness-aware AI design. By making our model, N2E, freely available on www.name-to-ethnicity.com, we hope to contribute to fulfilling NECs’ promise to uncover racial inequalities. De-biased NECs will be sharper tools for de-biasing our world.

## NECs and their (unknown) location in the bias landscape

### The genesis of NECs

If the similarity of your name and those around you were plotted in a naming network graph they would likely show up in close proximity to each other. Mateos et al. have visualised such a graph for 17 countries, with names being represented as nodes and shared forename–surname pairs as the edges between them (2011, p. 2). The resulting ‘names map’ bears striking similarity to a geographical map of the world. Thus, Mateos et al. confirmed in a computerised way what onomasticians have long observed—that names are reliable markers of cultural, ethnic and linguistic origin (Kandt and Longley 2018, p. 1). Today, naming networks stretch beyond the geographical areas in which the names originated as they are extended through international migration (Mateos et al. 2011, p. 1) and cultural exchange, e.g. of music or movies.

Therefore, it might be no surprise that the first attempts to use the co-existence of different naming networks came from a longstanding immigration country, namely the USA. In 1953, the California Department of Public Health developed the ‘Generally Useful Ethnicity Search System’ (GUESS) to assign Hispanic ethnicity based on the linguistic structure of last names (Fiscella and Fremont 2006, p. 1489). Since then, many other NECs have been developed, first in the field of public health and population genetics, and later in the social sciences (Mateos 2007, p. 243). The initial tools assign ethnicity based on the probabilities calculated from name reference lists. Most of them are confined to identifying just one or few ethnicities. Examples are Nam Pehchan (Cummins et al. 1999, p. 401) and SANAGRA (Nanchahal et al. 2001, p. 278).^1^

Subsequent NECs became increasingly complex, based on larger datasets and distinguishing between more ethnicities or nationalities. A much-used tool from this epoch is Onomap. Onomap comprises more than 600,000 names gathered from name registers of 26 countries. All these names have been classified into so-called ‘cultural ethnic linguistic’ groups. Onomap then calculates the probability of a name to belong to one of these groups (Lakha et al. 2011, p. 689). Another variant is the trigram-based classification from Schnell et al., which slices names into letter groups of three (e.g. ‘Hafner’ → ‘Haf’ – ‘afn’ – ‘fne’ – ‘ner’) (2013, p. 5). A further significant advance is EthnictyEstimator, which uses 51 million micro records from the 2011 UK Census (Kandt and Longley 2018, pp. 4–21). Some tools have been commercialised, such as NamSor^2^ and OriginsInfo,^3^ which on their website boast clients such as the United States Agency of International Development, the City of Boston, the airline Emirates (NamSor), Microsoft, the Labour Party, and the Premier League (OriginsInfo).

The latest generation of NECs rely on artificial intelligence and large datasets of personal names openly available on the Web. A major trend is to use Deep Learning methods, for instance in the form of word embeddings. Hereby, names are split into n-grams (unigrams ‘H’ – ‘a’ – ‘f’…, bigrams ‘Ha’ – ‘af’ – ‘fn’…, and so on) which are then set into relation with each other via unsupervised learning methods. At this, the algorithms’ learning method can vary. Lee et al., for instance, propose a LSTM recurrent neural network approach, which they applied on 17,653 Olympic athletes (2017, p. 2083). Ambekar et al. train Ethnicolr using Hidden Markov Models on 130,137 names scraped from Wikipedia (2009, pp. 2–3). For more examples of efforts to combine AI and online data, see Ye et al (2017, p. 2) and Ye and Skiena (2019, p. 3). However, the most ambitious NEC project arguably comes from Ye et al. themselves. To develop NamePrism, Ye and colleagues used a Naive Bayes classifier on 68 million names from emails and 6 million from Twitter (Ye et al. 2017, p. 6).

It has been common practice that NEC developers report the accuracy of their method by testing it on a ‘gold standard’, i.e. a list of names with self-reported ethnicities. Whereas most studies assess only their own method in this way (e.g. Kandt and Longley 2018, p. 6; Kozlowski et al. 2021, p. 5; Mateos et al. 2006, p. 3), others compare their method to the accuracy rates of other NECs (e.g. Ye et al. 2017, p. 6; Jain et al. 2022, p. 15). Another strand of research conducts *independent* assessments of NECs. For instance, several public health studies use patient records to test how recommendable the use of NECs is in a medical context. Lakha et al. (2011, p. 688) and Smith et al. (2017, p. 1) test Onomap, Brant and Boxall test NamPehchan (2009, p. 316) and Ryan et al. compare Onomap and NamPehchan (2012, p. 1).

This body of ‘NEC assessment work’ has two fundamental shortcomings. First, it has not yet covered the latest generation of AI-based NECs. Second, it is blind to intersectional differences in accuracy rates. If differences in accuracy rates are reported at all, they are mostly confined to the classified ethnicity groups. However, intersectionality studies show that the decisive difference might not be related to a *single* axis of differentiation (here: ethnicity), but to the re-enforcing interconnections between *several* axes (e.g. ethnicity coupled with gender, age, class…) (Cooper 2016, p. 385).

### The bias landscape of AIs

The hypothesis that intersectional biases might be inscribed in NECs seems plausible, as this phenomenon has already been detected in a wide array of AIs. In recent years, a rapidly growing body of ‘algorithmic fairness research’ has discarded the original hopes that AI could help keep the biases of human beings out of decision-making. Instead, AIs turned out to perpetuate, and even amplify, the human bias encoded in them (Hajian et al. 2016, p. 2125; Rozado 2020, p. 2).

In the field of facial recognition, the influential ‘gender shades’ report was the first to reveal intersectional accuracy disparities (Buolamwini and Gebru 2018, p. 1). Boulamwini and Gebru compared three programs that infer gender from peoples’ images (from Microsoft, IBM and Face + +). They found that all classifiers performed less well on female than on male faces, and worst on darker female faces (ibid., p. 8). Subsequently, many studies confirmed these findings for other facial recognition AIs: Acien for VGGFace and Resnet50 (2019, p. 584); Balakrishnan for Resnet50 trained on faces of celebrities (CelebA) and on the more balanced FairFace databank (2020, p. 548); and Raji et al. for Microsoft, Amazon and Clarifai facial recognition tools across tasks like gender prediction, age prediction and smile detection (2020, p. 146). For another image processing AI, which was trained to detect skin cancer, Zou and Schiebinger report the equivalent decrease in performance from ‘lighter males’ to ‘darker females’ (2018, p. 325).

But whether it is detecting smiles or cancer, the main underlying reason for the performance differentials is the following: highly unbalanced training datasets. The cancer detection AI was trained on 120,000 GoogleImages, of which fewer than 5% depict darker-skinned individuals (ibid., p. 325). It comes as no surprise that facial recognition datasets based on personalities deemed as ‘celebrities’ by Western pop culture, are unbalanced. However, other facial databanks turned out to be equally skewed. For instance, LFW contains 77.5% images of males and 83.5% of Whites (Buolamwini and Gebru 2018, p. 3); Adience 41.6% lighter males in comparison to 7.4% darker females; and IJB-A 59.6% lighter males and only 4.4% darker females (ibid., p. 6).

The study of other AI tools might not be as advanced in intersectionality, but the separate analysis of ethnicity and gender differences paints a similar picture. Take word embedding AIs for automated translation, text generation and Web search suggestions. On the gender axis, Bolukbasi et al.’s title ‘Man is to Computer Programmer as Woman to Homemaker’ illustrates the gender stereotyping in word associations (2016, p. 5). On the ethnicity axis, studies have shown systemic bias against minorities. For instance, names popular amongst African-Americans are disproportionately associated with negative terms (Rozado 2020, p. 2) and searching for these names online will more likely show ads for arrest records (Hajian et al. 2016, p. 2125). Once again, the main culprit is the training database. Models trained on digital text such as GoogleNews or Wikipedia display stereotypes just as much as those trained on historic text corpora (Garg et al. 2017, p. 35).

Another biased AI application is risk assessment in the criminal justice system. The company Northpoint falsely flags black defendants for recidivism twice as often as their white co-inmates (Silva and Kenney 2018, p. 16). Hamilton has shown that the AI tool Compas wrongly predicts Hispanics as ‘high risk’ in eight out of ten cases (2019, p. 1575). Similarly, 40% of false matches from Amazon’s Rekognition program—designed to detect criminals—involve people of colour (Khalil et al. 2020, p. 2). Again, training data explains these demerits. Algorithms built on data from a historically biased justice system are bound to have this bias hardwired into them (Silva and Kenney 2018, p. 16).

The long list of AI bias goes on as follows: advertisement algorithms show job ads for high paying jobs less often to women than men (Hajian et al. 2016, p. 2125; Mehrabi et al. 2019, p. 3); hiring software, trained on the CVs of those that have been successful within a firm in the past, label women and members of minorities as less suitable candidates (Yarger et al. 2020, p. 383); credit score algorithms deny funding to those who have previously been excluded from the credit system, namely women and minorities (Silva and Kenney 2018, p. 18).

These injustices have led to the emergence of an epistemic community warning about AI’s potential to have dangerous consequences for underrepresented communities (Mehrabi et al. 2019, p. 8), to exacerbate socioeconomic disparities (Gianfrancesco et al. 2018, p. 1544), to project toxic power-structures into the decision-making machinery of the future (Zou and Schiebinger 2018, p. 325) and to violate human rights (Rodrigues 2020, p. 1; Fukuda-Parr and Gibbons 2021, p. 40). These concerns have also reached policy circles, with the US (Altenburger and Ho 2019, p. 1), Canada (Engelke 2020, p. 2) and the EU (Vesnic-Alujevic et al. 2020, p. 1; Robinson 2020, p. 1; Stahl et al. 2022, p. 3) enshrining concerns about AI into their public policies.

These alarms did not remain without consequences. In their strive to make fairer AIs, researchers have created facial datasets for hitherto unrepresented communities, such as the Indian Movie Database or the Chinese Face Database (Khalil et al. 2020, p. 8). They have proposed sector-specific bias mitigation strategies in the fields of medicine (Vokinger et al. 2021, p. 1; Zhang et al. 2022, p. 1), public health (Zink and Rose 2020, p. 973) and law enforcement (Pastaltzidis et al. 2022, p. 2302). They have proposed AIs that can perform their tasks while suppressing sensitive attributes such as ethnicity and gender (Acien 2019, p. 591; Bolukbasi et al. 2016, p. 1; Papakyriakopoulos et al. 2020, p. 446). They have developed tools to address biases, such as AIF360, FairLearn and Aequitas (Pagano et al. 2022, p. 2).^4^ They have moved corporate giants as folows: After having been publicly audited, Amazon and Microsoft updated their facial recognition AIs with significantly lower differences in gender classification (Raji et al 2020, p. 147). And they have gained policy backing for fairness audits to serve as conditions for AIs’ accreditation (ibid., p. 145).

This track record is impressive—but it only covers a fraction of the entire world of AI. Therefore, we follow Rozado’s call to ‘help the fairness epistemic community [by contributing to a] more comprehensive exploration of the bias landscape’ (2020, p. 1). As NECs’ place within it is still unchartered, we set out to fill in this white spot on the map.

## Charting NECs in the bias landscape

### Methodology

To devise our methodology we grapple with five questions raised by the AI fairness literature: Which AIs to audit? Which tasks to audit? Which metrics to use? Which benchmark to use? How to design a fairer AI?

#### Which AIs?

Start with the choice of AIs to audit. Raji et al. have put together guidelines for ethical algorithmic auditing. Their first advice is to begin by questioning the ethical use of the AI itself (2020, p. 150). Otherwise, if auditors’ ‘seal of approval’ was based on a merely technical assessment, it might legitimise the use of technology that is inherently detrimental to certain communities. Raji et al. instance gender identification tools that can promote gender stereotypes and exclude non-binary individuals (ibid., p. 147).

Parallel to gender classification, NECs might be offensive by assigning individuals an ethnicity label they do not identify with. Also, just like gender, ethnicity is a social construct. ‘Ethnicity’ does not characterise the ‘essential’ characteristics of an individual, but rather the socially constructed behaviours *between* individuals (Luhmann 1997, p. 72; Hess 2004, p. 169). However, reifying ethnicity into an AI category assigned to an individual’s name might contribute to an essentialist understanding of ethnicity. Therefore, NECs must not be misused to draw conclusions about individuals. Instead, they should only be used to make group-level inferences about the distributions of ethnicities in large datasets. Some NECs, like EthnicityEstimator, ensure this by setting the minimum of names to 100 and only returning the overall distribution of ethnicities. Still, a further caveat remains: History has taught us that ethnicity or ‘race’ as statistical categories can reinforce ethnic stratification (Zuberi 2001, p. 17). In fact, the racialisation of data can be traced back to colonial projects and their disempowering of those labelled ‘ethnic’ (Godin 2007, p. 691).

So why even consider using NECs today? Because the ethnicity label is a double-edged sword, It can be used not only to reinforce, but also to mitigate inequalities. Focussing on one blade of the sword, the lesson some nations drew from the historic misdeeds was not to collect any ethnicity-related information. Here (e.g. France, Germany), the underlying credo is a ‘colour blind’ approach to justice. However, in nations that eventually switched to the other blade (e.g. UK), it was the ‘agenda for diversity’ that pushed for the re-inclusion of ethnicity-related questions in the census to address discrimination based on colour (Aspinall 2009, p. 1418).

This ‘colour conscious’ approach has become consensus in many fields of research (Mateos 2007, p. 244). In medical research, the Coronavirus pandemic laid bare the inequalities in health outcomes that had been less visible in ‘colour blind’ times, making pleas to collect more ethnicity-related data (e.g. Lakha et al. 2011, p. 688; Fiscella and Fremont 2006, p. 1482) more urgent than ever. In the social sciences, the BlackLivesMatter movement made researchers denounce the dearth of data even louder. Most studies depend on censuses, which are typically collected only every ten years and contain—if at all—merely a few ethnicity categories (Mateos et al. 2006, p. 2). For historical datasets, adding self-reported ethnicity in retrospect is impossible.

Research in which NECs were used to overcome such data scarcity abounds. They have been crucial in assessing disparities in the composition of patients (Lakha et al. 2011, p. 693), cancer incidences (Jacobs and Lauderdale 2001, p. 257; Coronado et al. 2002, p. 979) and mortality rates (Rosenwaike et al. 1991, p. 175). They have helped trace patterns of ethnic segregation in cities (Simpson 2004, p. 661; Mateos et al. 2006, p. 2), as well as in rural Africa (Harris 2015, p. 220). They have revealed discrimination against political candidates from ethnic minorities (Thrasher et al. 2017, p. 413), against ethnic scholars in the publishing industry (Kozlowski et al. 2021, p. 1), as well as the efficiency of equal opportunity policies (Johnston et al. 2004, p. 237). NamePrism^5^ alone has been used in over 200 research projects, and it is just one of many NEC providers.

These studies contribute to an evidence base for affirmative action. As this will be increasingly important in our multicultural future, improved ethnicity classification tools will be of major policy relevance. Therefore, we want to back NECs through our audit and contribute to their advancement.

Having affirmed the question about the ethical use of NECs, it remains to choose which specific NECs to focus on. We pick EthnicityEstimator,^6^ NamePrism and Ethnicolr.^7^ Our choice is informed by three considerations. First, all have thus far not been audited. Second, they display awareness of offering double-edged swords. EthnicityEstimator features the above-mentioned minimum group size for anonymisation. EthnicityEstimator and NamePrism require researchers to apply with a description of the research project for ethical screening. All three are developed by and for the scientific community, with clear mission statements of wanting to support ‘colour conscious’ research. Third, all three are free and user-friendly. The former quality places them in contrast to NEC providers whose paywalls restrict them to corporate use only (Namsor, OriginsInfo); the latter to computer science projects that are meant more as ‘proof-of-concepts’ of the underlying code rather than for public use (e.g. Schnell et al. 2013; Lee et al. 2017). EthnicityEstimator offers a Webpage for uploading a list of names and then downloading the calculated ethnic distributions. NamePrism has an API for bulk classification. Ethnicolr offers a Python package. Combined, these three considerations lead us to expect that EthnicityEstimator, NamePrism and Ethnicolr will be amongst the NECs most widely used in the future, and therefore most worthy of an audit.

#### Which tasks?

The next advice from Raji et al. is to be aware of the great responsibility of choosing which aspects of an AI to audit. By closing in on one aspect, other marginalised groups might be ignored (2020, p. 147). More than 90% of AI bias studies focus on gender, 50% on ethnicity, and only 10% mention other potential sources of bias, such as age, religiosity, political leaning, etc. (Rozado 2020, p. 2). However, the rare attempts at providing a comprehensive screening of biases revealed unreported bias types. Word embeddings disproportionally discriminate in terms of political leaning (against conservatives), socioeconomic standing (against the working class), age (against senior citizens) and religion (against Muslims and atheists) (ibid., p. 13). Balakrishnan reports that instead of gender, attributes like hair length, age and facial hair correlate most with performance biases of facial recognition AIs (2020, p. 547). In this jungle of potential bias drivers, even selecting the ‘right’ one to audit can have negative knock-on effects. It can lead to ‘fairness gerrymandering’, i.e. when optimising for fairness in the audited task diminishes fairness in others (Raji et al. 2020, p. 150).

Bearing all these caveats in mind, we nonetheless join the majority in auditing biases related to ethnicity, gender and age. As these represent the largest ‘minorities’, justice along those lines is one of the most pressing issues. Furthermore, in many jurisdictions the categories of ethnicity, gender and age are defined as ‘protected’ classes by constitutions and congressional acts (Laffin 2020, p. 1). Therefore, reducing bias in those domains is not only ethical, but also legally required.

#### Which metrics?

To decide how to measure fairness, we first need to ask what ‘fairness’ is. From Aristotle to Rawls, millennia of philosophy have not produced a universal definition. It is little wonder that AI fairness research, in its brief existence, has not solved the definitional dispute. Rather, a plethora of notions of what constitutes a ‘fair’ algorithm exists, which can be loosely grouped into two categories as follows.

The first deals with normative issues of association problems. A case in point is word embedding. Females are actually overrepresented amongst ‘homemakers’ and ‘nurses’–so if the values of word vectors correlate with this distribution, is the algorithm then biased or just realistic? (Rozado 2020, p. 3). A definition used in such contexts is that ‘algorithmic bias denotes the deviation of the algorithmic results from specific social expectations, based on epistemic or normative reasons’ (Papakyriakopoulos et al. 2020, p. 446). The proviso of ‘social expectations’ and ‘epistemic or normative reasons’ externalises the definitional problem to outside the scope of AI fairness research. But in fact, it touches the fundamental question: Should AI reflect the word as it is, or as we want it to be? (Zou and Schiebinger 2018, p. 326).

The second category deals with technical issues of classification problems. These classification problems differ from the abovementioned association tasks (e.g. ‘What is a nurse?’) as they have fixed categories into which an AI can categorise entities either correctly or wrongly (e.g. is this a nurse, yes or no?). NECs face such a classification problem: According to the ‘gold standard’ the person identifies as African—has the AI categorised her as such? Fairness definitions used in classification problems are, for instance, individual fairness, which posits that an algorithm ‘is fair if it gives similar predictions to similar individuals’ (Mehrabi et al. 2019, p. 11–12), or group fairness, which deems an algorithm fair if it has no deviation ‘from equal algorithmic outcomes at the group level for distinct demographic groups’ (Rozado 2020, p. 16).^8^

**Fig. 1:**
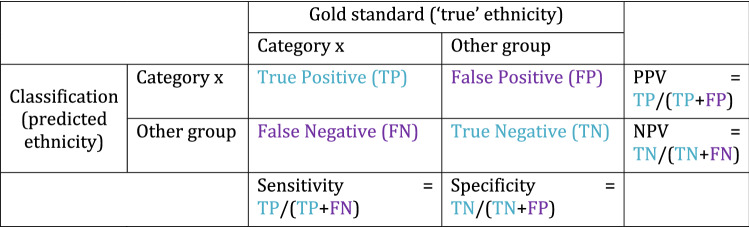
Confusion matrix

The comparison of ‘algorithmic outcomes’ illustrates how notions of fairness are operationalised through metrics. Many, increasingly sophisticated, metrics have been proposed but the most widely used are those derived from the confusion matrix.^9^ The matrix tabulates the predicted categories against the ‘true’ categories (see Fig. 1). Elements in the diagonal are correctly classified; elements outside it are misclassified. From this, four key measures are derived. *Sensitivity* is the proportion of category *x* correctly classified. *Specificity* is the proportion of categories other than *x* correctly classified. *Positive predictive value* (PPV) is the proportion of category *x* which are actually part of category *x*. *Negative predictive value* (NPV) is the proportion of categories other than *x* which are actually part of other categories (Mateos 2007, p. 254).

Those classifiers are regarded as the fairest whose sensitivity, specificity, PPV and NPV have the least variance between their classification categories. But even this quantitative approach is not immune to ambiguity. Assessing the recidivism AI Compas, both, the non-profit organisation ProPublica and Compas’ statisticians used metrics to prove their respective points. Using PPV, Compas found its AI to be fair, whereas using the false positive rate (= FP/FP + TN), ProPublica found it to be biased against Blacks (Hamilton 2019, p. 1558). This is just one of many examples of how contrasting measures can lead to conflicting impressions of an AI’s accuracy. Thus, it’s a tricky task to choose which metric to rely on. Is it more important to ‘catch’ all potential reoffenders? Then maximise PPV. Is it more important not to falsely flag reformed inmates? Then minimise the false positive rate. Ultimately, you can’t escape a value judgement.

We chose to focus our audit on sensitivity (also called true positive rate, hit rate and recall). Firstly, because this is the only measurement we can calculate for all NECs. This is the case as EthnicityEstimator’s anonymisation procedure does not let us draw conclusions about the make-up of TP, FP, TN, and FN behind the aggregate output.

However, we can approximate the true positive rate by, for example, uploading a file of 1000 Chinese names. If EthnicityEstimator predicts that 800 are Chinese, this corresponds to a true positive rate of 80%.

Secondly, because in comparison to the downsides of other fairness measures, those of the sensitivity approach are the most acceptable in the context of NECs. One such downside is that focusing on sensitivity can fail to uncover a high rate of false positives (Cortez 2019, p. 9). However, high false positive rates tend to occur in categories that are over-represented in the training data, *not* in under-represented groups. Thus, optimising the false positive rate would improve the performance for dominant social groups over minorities. Our judgement, however, is that it is more important to optimise for minority groups. Minorities are also the groups most NEC users focus on. The relevant question for their research is ‘how many of the group I’m studying are correctly classified?’ This is what sensitivity measures. For researchers interested in alternative measures, however, we provide an appendix stating the remaining confusion matrix metrics (except for EthnicityEstimator). There, we also report 95% confidence intervals for all measures, following the example of Besse et al. (2022). *P*-values are indicated throughout the study.^10^

Another shortcoming might be the selection of the ‘protected’ category which the matrix tabulates against the remaining categories. By focusing on a ‘protected’ attribute, the approach might hide unfairness in other attributes (Verma and Rubin 2018, p. 5). However, our intersectional study design also scrutinises differences in sensitivities along other attributes, namely gender and age groups. Nevertheless, it is true that we might miss further imbalances, e.g. along class.

A further shortcoming is the problem of ‘infra-marginality’. It states that as sensitivity measures fairness by looking at the mean of aggregate groups, enforcing sensitivity as fairness criterion might encourage misclassification if the variances of the underlying distributions vary across groups (Corbett-Davies and Goel 2018, p. 11). However, we mitigate the problem of infra-marginality, as the underlying distribution of names does not vary across groups. We choose the same number of names for each ethnicity to test the NECs, as well as to train our own NEC.

Lastly, another shortcoming is that sensitivity fails to disentangle ‘model bias’ from ‘input bias’. Input bias stems from the imbalances amongst population groups in the real world, which can be expected to ‘sneak into’ any dataset. Model bias denominates the bias that is added to input bias due to the specificities of the machine learning pipeline (Hellström et al. 2020, p. 5). By measuring sensitivity, we only point out whether algorithms produce the same rates of true positives, i.e. whether they are equally ‘right or wrong’. In strict machine learning terms, we measure balance, not bias, as we cannot specify how much model bias an NEC adds to the underlying input bias. Future research might investigate causal fairness inference metrics (see Loftus et al. 2018; Zhang and Bareinboim 2018) to speak to the role of NECs’ algorithm ‘proper’. But for this study, we define bias as the *overall* imbalance of sensitivities in NECs’ classifications. Conversely, we regard those classifiers as fairer whose sensitivity has the least variance between the different ethnic groups. Aristotle, Rawls and AI programmers might continue disagreeing on the theoretical nuances of fairness definitions, but for NEC practitioners this broad definition is a first indication of their tools’ overall fairness.

#### Which benchmark?

A benchmark is the ‘gold standard’ dataset against which predictions are validated. It is a crucial component of algorithmic audits since the quality of a benchmark can significantly influence the results (Gorana and Mishra 2021, p. 1). Two following benchmark characteristics are key: variety and volume.

Variety, i.e. a range of different labels, is needed for intersectional analysis. To assess performance differences at the cross sections of ethnicity, age and gender, we need a benchmark annotated with all these three categories.

Volume, i.e. a large amount of data, is indispensable for statistical accuracy. This is not news for statistical analysis in general, but fairness auditing comes with special requirements. Raji et al. advise that if one demographic is underrepresented in a benchmark, it should not be used to assess the AI’s performance within that demographic (2020, p. 147). Rather, an equal distribution of sub-group volumes is required. The reason is simple: Say a benchmark contains 90% Brits and 10% Nigerians. Even if the algorithm only randomly sorts people, the probability of correctly classifying a Brit is much higher than that of correctly classifying a Nigerian, due to the underlying distribution in the benchmark.

To equalise a benchmark researchers can supplement the existing benchmark with underrepresented individuals. However, this ‘up-sampling’ puts marginalised groups at a higher risk of predatory data collection practices (e.g. pictures of Whites might be taken from open licence celebrity databases, pictures of Blacks from non-consensual video surveillance) (Raji et al. 2020, pp. 145–147). Therefore, we opt for achieving an equal distribution through reducing the number of over-represented individuals. Nevertheless, for ‘down-sampling’ to be an option, the original volume of the benchmark needs to be large enough to still assure statistical significance after the sub-group volumes are equalised to the volume of the smallest group.

We find the conditions of variety and volume to be met in the UK government’s CompaniesHouse^11^ business register. The database comprises 7.3 million records of company officers. Of all the groups we want to test, the fewest records are of Caribbean woman under 35 years (*s* = 1013). So after equalisation we still have a benchmark volume of *n* = 11,143 for EthnicityEstimator (= *s* × 11 ethnicity categories), *n* = 9117 for NamePrism (= *s* × 9 ethnicity categories), and *n* = 12,156 for Ethnicolr (= *s* × 12 ethnicity categories, which exceeds those used in other AI audits (e.g. *n* = 1270 in Buolamwini and Gebru 2018, p. 2).

CompaniesHouse offers the necessary data variety as each record states officers’ age (date of birth), gender (prefix Ms, Mrs or Mr) and nationality. We aggregate nationalities to match the ethnicity groups used by the NECs (e.g. French, German, Austrian → ‘European’; Nigerian, Zambian, South African → ‘African’). This procedure can be problematic in case of a lack of exact correspondence between the benchmark’s categories and those of the AI’s. We work around this by finding the best matches (e.g. NamePrism’s categories ‘Celtic’ matched with Anglo-American nationalities; ‘Nordic’ matched with Scandinavian nationalities; and ‘Muslim’ matched with Arab nationalities).

#### How to build a fairer AI?

**Fig. 2:**
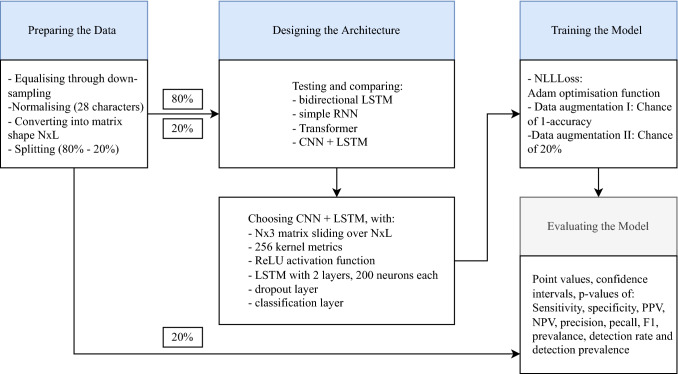
N2E machine-learning process

We use the treasure trove that is CompaniesHouse data to train a fairness-aware NEC, which we call N2E. In order to increase fairness by minimising performance differentials between ethnic groups, we follow the workflow illustrated in Fig. 2.

(1) *Preparing the data*: We split the CompaniesHouse data into two fractions, one for training (80%), the other for testing (20%). Adopting best practices to reduce biases in the input data, we follow Japkowicz and Stephen (2002, p. 429) in assembling a balanced set of names by down-sampling to the least-occurring ethnicity (i.e. 69,836 for Scandinavian, total training size = 558,688). The names are then normalised to the latin alphabet, (e.g. ê becomes e) and converted to lowercase. Spaces and hyphens are preserved, which makes the final alphabet size 28. We make each name numerically processable by converting it into a matrix of the shape *N* × *L*, where *N* corresponds to a vector assigned to each letter in the alphabet (vector length of 200 randomly assigned numbers, e.g. *a* ≙ [0.234; −0.134;…0.759]), and *L* to the amount of letters in each name (e.g. Ann = *a* ≙ [[0.234; −0.134;…0.759]; *n* ≙ [0.546; −0.721;…0.015]; *n* ≙ [0.546; −0.721;…0.015]).

(2) *Designing the architecture*: We set up and compare the following architectures: a bidirectional long-short term network (LSTM), a simple RNN, a transformer and a combination of a convolutional neural network (CNN) (see LeCun et al. 1998) combined with an LSTM (see Gers et al. 2000). The latter, i.e. the combination of CNN and LSTM, achieved the highest accuracy rates and was therefore chosen as N2E’s model architecture. This architecture is inspired by Lee et al.’s model (2017, p. 2083), but replaces their n-gram embedding technique with a single, one-dimensional convolutional layer. In the convolution a learnable kernel matrix of the shape *N* × 3 ‘slides’ over the ‘*N* × *L*-shaped’ input matrices. Thus, each convolution step processes three matrix columns (i.e. three letters) simultaneously. This is comparable to using three-gram embeddings but was found to increase performance. The convolution is applied with 256 different kernel matrices to produce 256 feature-maps. These feature-maps are then passed into the ReLU activation function (see Fukushima 1975) before being fed to the LSTM, consisting of two internal layers with 200 neurons each. The LSTM’s output is forwarded into a dropout layer (see Srivastava et al. 2014) to reduce overfitting, and, lastly, into a classification layer with a logarithmic-softmax activation function. This final layer returns a log-probability distribution *P*(*c* = Ci | *X*), i.e. the probability that the class c of the input name *X* is Ci, with *c* ∈ Ci. In other words, it returns the ethnicity to which a name most likely belongs.

(3) *Training the model*: We use the Negative-Log-Likelihood loss function (NLLLoss) to calculate the loss between the highest log-probability value of the prediction and the index of the ‘gold standard’ ethnicity stated in the CompaniesHouse data. Then, the model’s parameters are being updated by the Adam optimisation function (see Kingma and Ba 2014) with a weight decay of 1e-5. We choose a batch-size of 512 and a learning-rate starting at 0.001, which reduces by a factor of 0.95 every 100th iteration. Now, we test another best-practice from fairness-aware AI training, namely data augmentation. Following methods outlined in Chawla et al. (2002, p. 321), we synthetically generate new names by switching first and last names of two individuals belonging to the same ethnicity, thus creating a new name. In an iterative process we approach the optimal augmentation level for each ethnicity class by calculating the accuracy of each class after every training epoch. To calculate how much a class should be augmented, we subtract its accuracy from 1 and use this value as the chance that any name of this class gets modified in the next epoch. Thus, classes with a smaller accuracy have a higher chance of augmentation. Consequently, the training sample size for this class will increase, increasing the probability for accurate classification. However, this augmentation strategy did not significantly improve classification accuracy in comparison with using the same augmentation chance for each ethnicity, while slowing down the training process. Therefore, the final model made available on www.name-to-ethnicity.com applies a general augmentation chance of 20%. We make our code available on GitHub.^12^ How much improvement these measures brought will be seen in the following chapter, in which we compare our NEC’s bias to those of the other NECs.

### Analysis

A word of caution: The following audit should not be read as a comparison of different AIs’ accuracies. This is because the number of ethnicity categories varies between AIs, pre-establishing different base-probabilities. Imagine an NEC that only distinguishes between two categories. Given an equalised benchmark, even a random algorithm would achieve an accuracy of 50%. If that random algorithm had to classify into ten categories, its base-probability would drop to 10%. Therefore, instead of accuracy differentials *between* NECs, the audit scrutinises accuracy differentials *within* NECs. However, we can compare these ‘within’-differences using the average deviation in sensitivities.

$$\mathrm{Average\,Deviation}= \frac{1}{n}{\sum }_{i=1}^{n}\left|\left({x}_{i}-m(X)\right)\right|$$

With *m*(*X*) denominating the average sensitivities of an NEC, *n* the number of categories and ${x}_{i}$ the sensitivity of each respective category, average deviation measures the spread of sensitivities. By dividing through *n*, the formula accounts for the different number of categories, making NECs more comparable.

Furthermore, the audit is not a comprehensive overview of what went wrong in NEC programming. In AI ‘production’, bias can creep in at many steps. Frameworks group the potential biases into stages: two stages (data input and algorithm) (Mehrabi et al. 2019, p. 1); three stages (pre-processing, in-processing and post-processing) (Hajian et al. 2016, p. 2125; Laffin 2020, p. 2); or five stages (input, operations, output, users and feedback) (Danks and London 2017, p. 4691). As NECs’ algorithms are ‘backboxes’ our search for explanations of their overall imbalances focuses on the input stage. However, this first step is regarded as the most critical anyway. As this step serves as the foundation of all proceeding computations, the results can only be as good as the data entered into the algorithm (Zou and Schiebinger 2018, p. 325). The data entered into NECs is defined by two main characteristics: naming conventions and the distribution of names. For each axis of differentiation (ethnicity, gender, age) we consider these two sources of input bias in turn.

#### Ethnicity bias

##### Ethnicity bias as expected?

Knowing that AIs reproduce dominant power structures, we expect NECs’ biases to resemble those detected in other AIs. This gives rise to the hypothesis that categories from the marginalised Global South might receive lower accuracies than those from the dominant Global North.

**Fig. 3:**
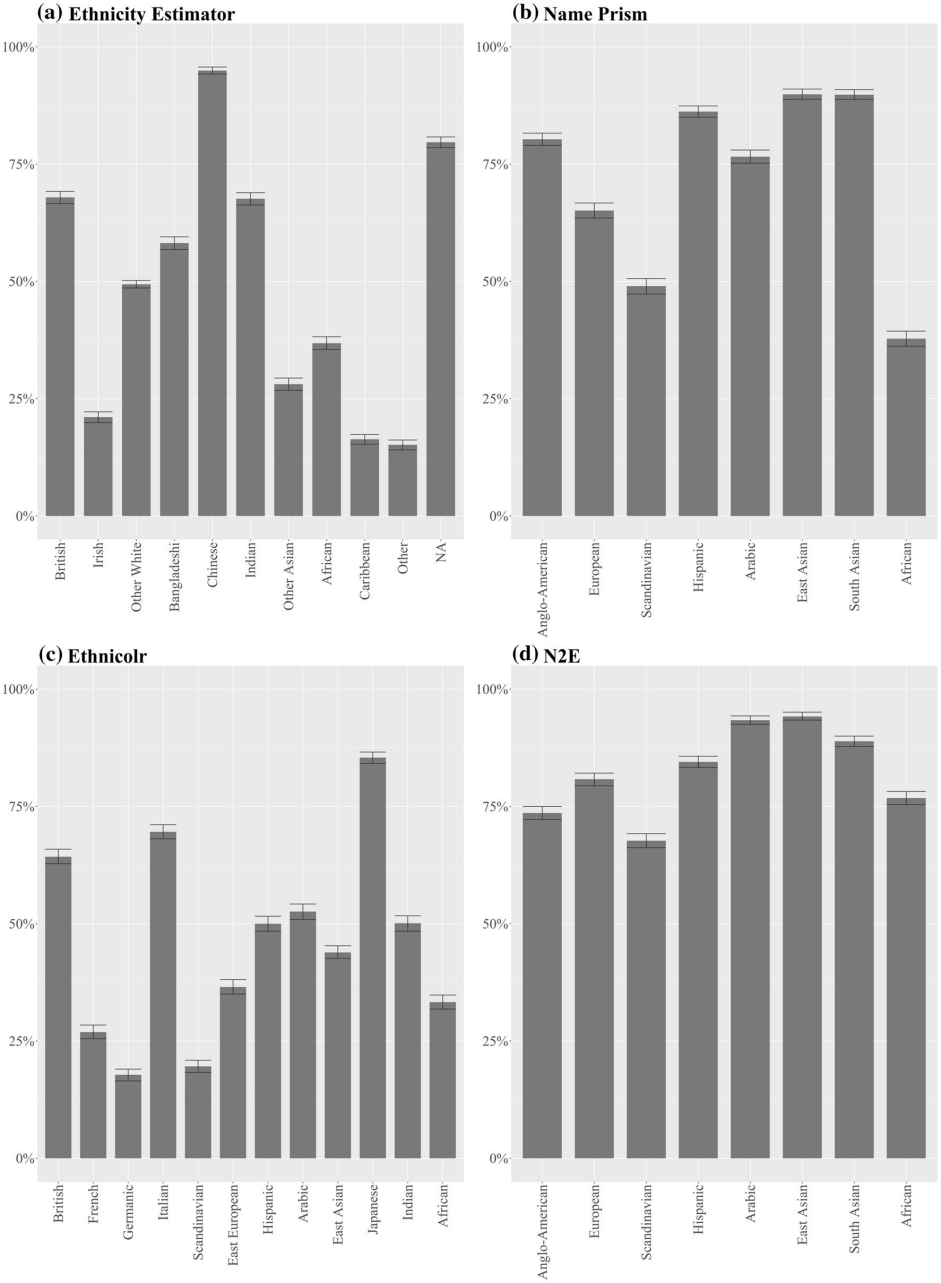
Sensitivity by ethnicity

Our audit confirms this hypothesis for the ‘extremes’ but not for the ‘in-between’ countries. At the ‘extremes’, measured either in terms of average income and living standards (e.g. Naustdalslid 1977, pp. 207–208) or profitability of production (e.g. Wallerstein 2004, p. 18), world systems scholars unanimously place Anglo-American nations on the one side, and African nations on the other. For these two opposites the three tested NECs perform homogeneously. As illustrated in Fig. 3, all achieve some of their highest sensitivities for ‘Anglo-American’ (EthnicityEstimator: 68%***; NamePrism: 80%***; Ethnicolr: 64%***) and the lowest for ‘African’ (EthnicityEstimator: 37%***; NamePrism: 38%***; Ethnicolr: 33%***). This means that if you are an American, you have an up to 80% chance of being correctly classified. If you are African, your best chance is 38%.

However, between this black-and-white contrast lie many categories that do not fit into the ‘Global South vs. Global North’ hypothesis. For example, the best-classified categories are from the Global South: 90%*** sensitivity for East and South Asians in NamePrism, and 95%*** for Chinese in EthnicityEstimator (Fig. 3a, b). Additionally, some Global North categories have mediocre sensitivities. For instance, 49%*** for Scandinavians in NamePrism and 27%*** for French and 18%*** for Germanic in Ethnicolr (Fig. 3b, c). How can we make sense of this picture, with predictable extremities but otherwise mixed biases?

##### Ethnic naming conventions as explanation?

For parents, picking their baby’s name might feel very individual. However, being embedded in the ‘cultural politics of naming’ these independent decisions are in fact systemic (Girma 2020, p. 18). Being culturally dependent, these systems of naming conventions influence the accuracies NECs can achieve for different ethnic categories. Naming systems evolve independent of migration as well as specific to immigration contexts. Therefore, it might make a difference whether NECs source their input data from a migration-independent context (e.g. NamePrism from Twitter, Ethnicolr from Wikipedia) or post-migration (EthnicityEstimator from the UK Census), where all ‘ethnic’ names are those of immigrants.

Post-migration, naming systems can be altered through naming assimilation. This occurs when immigrant parents forego names from their home country in favour of those common in the receiving country. Myriads of studies have documented the discrimination that occurs solely on the basis of ‘foreign’-sounding names on the job market (Carlsson and Rooth 2008, p. 1), housing market (Boscha et al. 2010, p. 11) and in social contexts (Girma 2020, p. 16). Seeking to lower these socio-economic barriers for their children, African parents in the US might opt for ‘Jackson’ over ‘Quaro’. This Anglicisation of traditional ‘Black’ names has its counterpart in Europe (Schnell et al. 2014, p. 231) and most other immigrant destinations as it reflects the mode of integration into the dominant host society.

Therefore, Schnell et al. opine that naming assimilation leads to under-detection of well-assimilated groups in NECs (2014, p. 246). Measured by indicators such as income, employment rates and language acquisition, Schnell et al. find that ‘better’ integrated immigrants are more often missed in name-based sampling.

However, whereas this might be true within ethnic groups (e.g. more integrated Turks vs. less integrated Turks), our audit finds no clues that this would be the case between groups (e.g. Turks vs. Chinese). The sensitivities achieved by EthnicityEstimator (Fig. 3a) indicate no correlation between integration and accuracy. On the contrary, Pakistanis have a high sensitivity of 80%*** and Africans only 37%***, even though, when measured in income, wealth and employment, both groups are furthest apart from the average amongst ‘Whites’. Similarly, Chinese (95%***) and Caribbeans (16%***) are on opposite sides of the sensitivity spectrum, but both enjoy the smallest gap to ‘Whites’ (ONS 2020). Furthermore, comparing the performance of EthnicityEstimator to the two other NECs (Fig. 3b, c) shows that, regardless of whether the training data is ‘post-migration’ or ‘migration-independent’ the sensitivities tend to be similar. This indicates that names remain tokens of cultural affiliation even in an era of global migration. Therefore, instead of the personal migration history of the classified individual, the underlying naming system blocks which are the cementations of centuries of migration—or lack thereof—seem to be more relevant for explaining accuracy differentials.

The ‘informativeness’ of these naming systems is driven by three main factors. First, the cultural mix within a region. Depending on the extent of historic migration, once homogeneous naming systems can become variegated. Lakha et al., for instance, attribute their finding that Onomap frequently misclassifies people born in Poland as Germans, and people born in Britain as South Asians, to the countries’ complicated histories of migration (2011, p. 693). These interwoven naming systems are also likely the underlying reason why the NECs tested here classify ‘melting pot’ categories, such as ‘British’, ‘Anglo-American’ and to some extent ‘European’, not as accurately as traditionally migrant-sending Asian regions.

The second factor is the ratio of people per name. For instance, nearly 40% of Vietnamese share the surname Nguyễn. The 14 most common surnames account for over 90% of the Vietnamese population. In contrast, the US’ most common surname, Smith, makes up only 1% of the US population and the 14 most common surnames amount to only 6% (Nosowitz 2017, p. 1). Given these underlying distributions, AIs will have an easier time classifying Vietnamese than US Americans.

Lastly: the cultural uniqueness of names. Whereas some names are specific to one cultural origin, others are spread amongst multiple origins. Through word embedding modelling, Mateos et al. demonstrated that 77% of surnames can be unequivocally assigned to one cultural-ethnic-linguistic group. The remainder forms part of culturally close groups, such as Slavic, Germanic or Nordic languages (2011, p. 3). By combining first and last names, Kandt and Longley increased the share that can be clearly assigned to one ethnic group to 84% (2018, p. 7). Both research teams remark that non-European groups, mostly those of Asian origin, have the most clear-cut naming boundaries (ibid.; Mateos et al 2011, p. 9). This might be the reason why all tested NECs perform extraordinarily for Asian categories, and poorly for the categories ‘European’, ‘Scandinavian’ and ‘Germanic’.

Apart from the ‘raw material’ of names, the way programmers assemble this ‘raw material’ into categories can influence accuracy rates. As a rule of thumb, more narrowly defined groups (e.g. Japanese) work better than broader ones (e.g. East Asian), as the former have a higher likelihood of being homogenous. Sometimes the categories might be a given, like the Census categories for EthnicityEstimator. Some of the Census categories are ill suited, such as Irish (21%***), which is too similar to British, and ‘Other Asian’ (28%***) and ‘Other White’ (${50\%}^{ns}$), which are pools for unrelated remainders of names. The creators of NamePrism and Ethnicolr might be freer to devise categories that are better aligned with cultural–linguistic boundaries. But these two NECs also feature groups whose performance is likely to be enhanced (e.g. Ethnicolr: Japan 85%***) or dampened (e.g. Ethnicolr: East Asian ${44\%}^{ns}$) on the basis of category assembly.

The seemingly technical issue of category design is a product of power structures. In the case of Census categories, public authorities determine which groups are relevant enough to look at. In the case of NamePrism and Ethnicolr the limiting factor is the amount of names per category that needs to be large enough to train an AI. This correlates with global power imbalances, as it is a matter of lack of access (Twitter) or lack of representation (Wikipedia).

##### Ethnically skewed input distribution as explanation?

NEC designers can control the input distribution of names. However, up- or down-sampling techniques have not been employed consistently. Therefore, where category design and naming conventions are the same, an input skew still explains the differences in the average deviation of sensitivities between NECs.

*EthnicityEstimator*. Between ethnic groups EthnicityEstimator has the greatest average deviation of sensitivities (23%***) of the tested NECs. The census seems like the most representative input source of all three NECs. But it might be representative of the wrong scale: the UK instead of the world. With over 56 million Brits, no wonder that EthnicityEstimator classifies them accurately (68%***). However, the rest of the accuracies follow input volumes only loosely. For instance, Pakistanis are the largest ethnic minority but the accuracy for Chinese outperforms them (80%*** vs. 95%***). Thus, it seems that naming conventions and the configuration of ethnicity categories are more decisive than input distributions for EthnicityEstimator.

*NamePrism*. This NEC has a moderate average deviation of sensitivity between ethnic groups (16%***). Email and Twitter are world-wide phenomena and therefore more globally representative. Nevertheless, there are differences in the usage of these services between countries. The designers of NamePrism, for instance, gathered an order of magnitude more names from the UK than from South Africa, even though both countries have a similar population size. The authors aim to mitigate this by assigning priors to names and by adjusting the real population of countries (Ye et al. 2017, p. 5). However, this seems not to fully have done the trick as the PPV and NPV values indicate (see Appendix). A low PPV of 33%*** for Anglo-Americans indicates that only 33% of those classified as Anglo-Americans were truly Anglo-Americans. The remaining 67% of classified Anglo-Americans were wrongly placed in this category—a clear sign of over-prediction due to over-coverage. In contrast, a high PPV of 83%*** for Africans means that over 83% of those classified as Africans were truly Africans, indicating under-prediction.

*Ethnicolr*. The NEC’s moderate average deviation (16%***) might be attributable to its training base, Wikipedia, a seemingly open and global platform. In fact, however, the distribution of entries by language is heavily skewed towards the Global North. For instance, there are 6.4 million entries in English, 2.6 million in German, but only 7314 in Somali (Wiki 2022). The inequality is even starker for biography entries, with five Global North countries accounting for 62% of Wikipedia’s biographical coverage (Beytía 2020, p. 806). The designers of Ethnicolr reveal the distribution of biographies which they gathered through their webcrawler. For instance, 39,735 Anglo-American, 7815 Japanese, 3819 African and 3617 German (for the full list see Ambekar et al. 2009, p. 52). This is surprising, as the crawled distribution does not match the expectation that biographies should follow overall article distribution. But the crawled distribution explains why Ethnicolr fairs well for Anglo-Americans (64%***) and Japanese (85%***); and badly for Africans (33%***) and Germans (18%***). Nevertheless, a more recent Wikipedia crawl has resulted in a distribution more in line with expectations, e.g. 8624 British, 29,271 French, 35,101 German, 17,790 Japanese (for full list see Treeratpituk and Giles 2012, p. 1143). Treeratpituk and Giles gathered the data to devise a name verification system, but in line with what we expect for NECs, they report a higher sensitivity for Germans (85%) and French (80%). The improvement shows that input volume and distribution matters. In conclusion, while Wikipedia itself might be ethnically skewed in predictable ways, our audit of Ethnicolr still resulted in some unexpected accuracies as the crawling itself brings in further distortions.

#### Gender bias

##### Gender bias as expected?

**Fig. 4:**
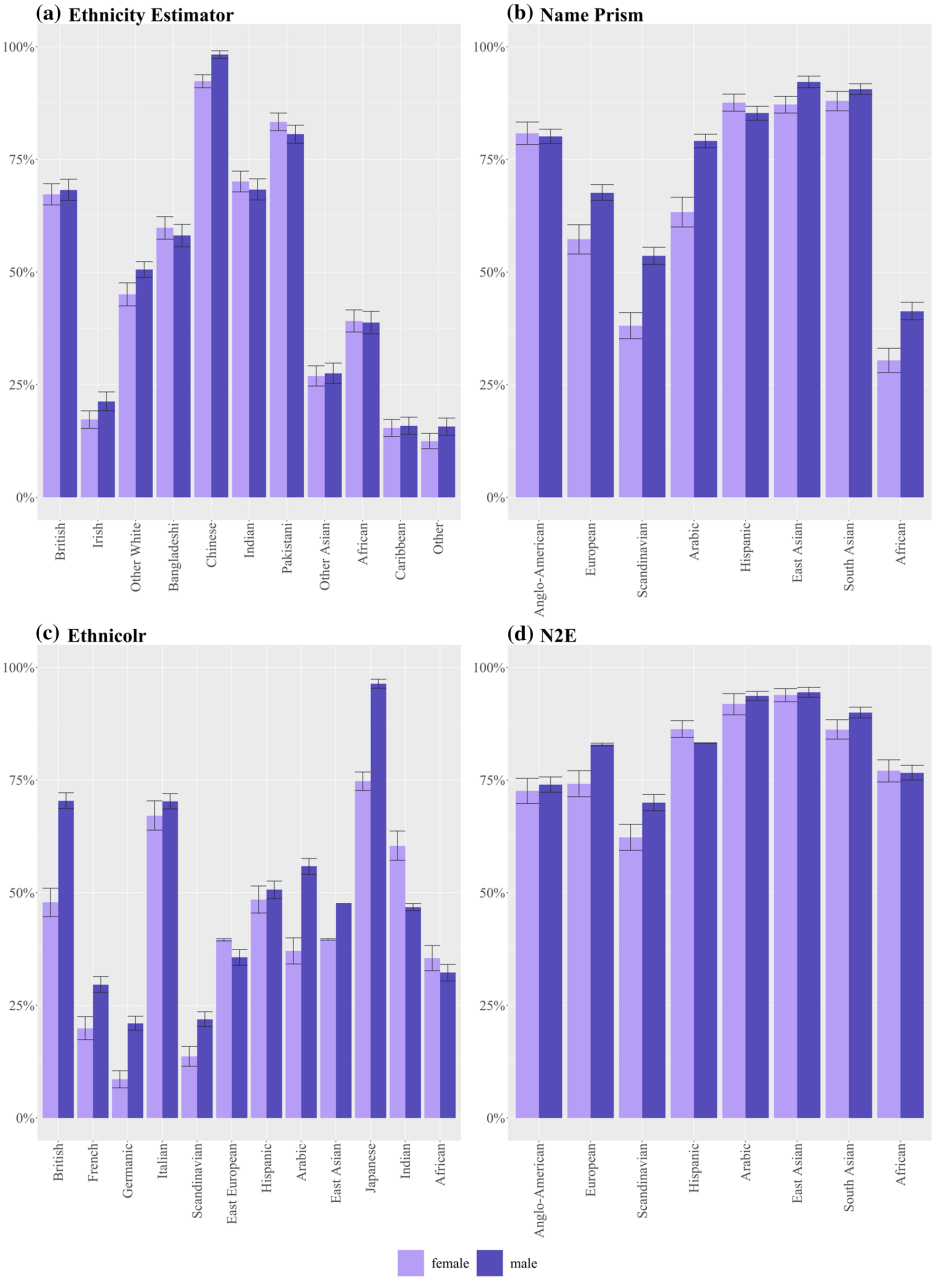
Sensitivity by gender

We expect NECs, like the AIs described above, to work better for men than for women. Indeed, two studies have made this finding in their assessment of Onomap (Lakha et al. 2011, p. 691; Kandt and Longley 2018, p. 9). In our assessment we find this ‘broad-stroke’ picture to be confirmed as well: Out of the 31 categories in the three tested NECs, over two thirds have higher sensitivities for males than females (see Fig. 4a–d). However, our intersectional analysis allows us to discern that this varies between categories of the Global North and the Global South. Out of the nine categories that worked better for women, seven are found in the Global South (EthnicityEstimator: Bangladeshi 60%***female vs. 58%***male, Indian 70%***female vs. 68%***male, Pakistani 83%**female vs. 81%***male, African 39.1%***female vs. 38.8%***male; NamePrism: Hispanic 88%***female vs. 85%***male; Ethnicolr: Indian 60%***female vs. ${47\%}^{ns}$ male, African 36%**female vs. 32%***male). Nevertheless, the size of the sensitivity differentials in these ‘women-better’ categories is relatively minor, ranging from 1% to 5% points, with the maximum being 13% points for Indians in Ethnicolr. In contrast, the size of the differentials in the ‘man-better’ categories is up to 24% points, with differentials around 10–15% points being the norm (e.g. NamePrism: Arabic ${63\%}^{ns}$ female vs. 79%***male, European: 57%***female vs. 68%***male; Ethnicolr: British 48%***female vs. 70%***male). How can we explain the more accurate performance for men, which is unequivocally the case in the Global North but not in the Global South?

##### Gendered naming conventions as explanation?

Naming a baby is probably the first act of imprinting gender on a new living being. Naming itself is a gendered process with parents’ preferences being different for boys and girls. This leads to gender-specific naming systems that might be at the root of NECs gender-skewed performance.

First, the phenomenon of post-migration name assimilation is gendered. Immigrants choose names from the host country more often for their daughters than their sons (Lieberson et al. 2000, p. 1249, Sue and Telles 2007, p. 1383, Gerhards and Tuppat 2020, p. 598). The latter study, for instance, finds a 35% points higher likelihood of choosing name assimilation for female descendants (ibid., p. 610). Possible reasons are traditional gender attitudes, in which boys are seen as representatives of ethnic or family traditions and girls as more in need of protection from name-based discrimination (Sue and Telles 2007, p. 1411). Gerhards and Tuppart have found a negative correlation between the index of gender equality in immigrants’ home country and the size of the gender name assimilation gap (2020, p. 611). In their study on Germany, Muslims are found to display the greatest gap. This might also be the case in the UK, where Muhammad is the 12th most popular boys’ name, with no equivalent accumulation of any Muslim girls’ name (Pilcher 2017, p. 814).

Given that EthnicityEstimator is trained on ‘post-immigration’ data, the gender gap in name assimilation should make females less well classifiable for this NEC. In reality, however, EthnicityEstimator has the smallest average deviation in sensitivity between the genders of all three tested NECs. Moreover, for the presumably most ‘gender-traditional’ Muslim groups EthnicityEstimator performs slightly better for females than for males (Bangladeshi 60%***female vs. 58%***male, Pakistani 83%***female vs. 81%***male). Therefore, gendered name assimilation seems to occur on a scale not significant enough to distort NECs.

Second, the degree of ‘genderedness’ of naming systems. Almost all systems use gender-specific names (Handschuh 2019, p. 550). The rare examples that don’t are overwhelmingly found in the Global South. These are mostly cultures that do not rely on a predetermined set of names but on symbolic nouns, such as spirits (Watson 1986, p. 621), concepts found in their holy scriptures (Price 2013, p. 7) or the name of the last person deceased in the community, independent of the dead’s gender (Jacobson 1995, p. 437). In such symbolic rites the named person is attributed to a spirit/concept/deceased ancestor. In contrast, in the more individualistic cultures of the Global North the name is attributed to the person, in order to reflect her characteristics, including gender.

Within these gender-differentiating naming systems, it is common that girls’ names far outnumber boys’ names. In the US, for instance, the share of names represented in the 1000 most frequent names is 75% for girls and 86% for boys. New girls’ names are invented at a rate of 2.3 new names per 10,000 for girls, and 1.6 for boys (Hahn and Bentley 2003, p. 121). The scholars attribute this to naming customs in patriarchal societies–an indicator that not only immigrants but also ‘settled’ people in Western cultures associate masculinity with ‘tradition’ and femininity with ‘creativity’. This imbalance of boys’ and girls’ names is further enhanced by the ‘gender-equality paradox’: The more dominant the value of gender-equality is in a society, the more gendered are its names (Vishkin et al. 2021, p. 1). The greater spread of female names might help explain why in the Anglo-American categories, as some of the most gender-equalitarian and individualistic societies, NECs classify females worse than males (Ethnicolr: 48%*** vs. 70%***, EthnicityEstimator 67%*** vs. 68%***).

All tested NECs take as input ‘forename + surname’-pairs. The first two reasons highlighted referred to forenames, shaped by culturally gendered preferences. The second two deal with surnames, which are often legally gendered. Surnames are an invention from the Global North. Their rise is closely tied to the emergence of private property and modern law (Scott et al. 2002, p. 4). Such systems create an interest of companies and states in tracing individuals, which is only possible with specific and officially documented names. The spread of surnames to non-Western countries was often imposed through colonialism, like in Ghana and Pakistan (Boxer and Gritsenko 2005, p. 37) or through state-led Westernisation, like in Turkey (MacClintock 2010, p. 284). Even today, parts of the Global South, especially in Asia and Africa are not covered by the surname-regime. Examples are Myanmar and Indonesia, with the former Indonesian president Suharto only having this one name (Price 2013, p. 7).

It might be unlikely that individuals with just one name move to the UK and end up in EthnicityEstimator’s dataset. But the example of Indonesia’s president shows that they may well have a Wikipedia entry or a Twitter account, thus being potentially included in Ethnicolr and NamePrism. However, if both genders do not bear surnames, this would not contribute to the gender gap anyway. In contrast, in surname-based systems the gendered rules of how surnames are structured make a great difference.

Third, gender-specific ways of appending surnames. Many systems use appendixes or morphs to indicate group-belonging in the surname. This is what might have gotten football fans confused about the players of the Icelandic national team: Edmundsson, Hansson, Jónsson… all the names sound similar as all men are named by appending –son to their father’s name. The equivalent for women is –dóttir (Kvaran 2007, p. 314). Other examples are the Russian -ovi/ -ovna (Hengst 2007, p. 623), the Swedish -son /-dotter and the Arab bin/binti (Okal 2018, p. 10–11). The custom as such is paternalistic, as there exists no recorded case in which surnames are derived from mothers’ names (Handschuh 2019 p. 557). But the degree to which male and female names are affected varies nonetheless. ‘Symmetric systems’ use appendixes for both genders, ‘asymmetric systems’ only for one gender. Handschuh finds a clear areal bias for symmetric and asymmetric patterns. Whereas Europe and the Caucasus favour asymmetrical marking, in South Asia and Africa the symmetrical system is dominant (ibid., p. 562).

NECs can be hypothesised to function with less bias in symmetrical systems. If both genders equally bear a marker, it is more likely that they can be classified equally well. This is difficult to verify in our audit, as the NEC categories do not map onto affix-regimes. However, it is conceivable that within the categories the structure of affixes contributes to ‘symmetrical’ Asian categories displaying less gender bias than ‘asymmetrical’ European ones.

Fourth, marriage naming conventions. Often, these consist in the bride shedding her birth name in favour of her partner’s name. It has been recognised that this practice distorts the functioning of NECs for women marrying outside their ethnic group (Fiscella and Fremont 2006, p. 1491; Mateos 2007, p. 5). However, this broad-stroke observation overlooks geographical variations in marriage naming practices. Some jurisdictions mandate women to change their maiden names by law. This is the case, for instance, in Turkey and other Arab nations (MacClintock 2010, p. 284). It might therefore be a contributing factor for women being classified much less accurately in these regions (e.g. NamePrism: Arabic ${63\%}^{ns}$ female vs. 79%***male; Ethnicolr: Arabic ${38\%}^{ns}$ female vs. 56%***male). In others, it is not a legal requirement but, nevertheless, customary practice. Cases in point are the US, where 90% of women choose to change their names (Gooding and Kreider 2010, p. 681), and the UK where 94% do so (Valetas 2001, p. 1). This might be related to lower sensitivities for women in the Anglo-American categories. In contrast, other world regions, mainly in the Global South, do not practice marriage name changes. Chinese, Vietnamese, Cambodians, Korean, South Asian Muslims, and Latinas all retain their names (Price 2013, p. 1). Consequently, women stay more accurately classifiable in NECs, adding a further explanation for NECs’ smaller gender sensitivity gaps in the Global South than the Global North.

##### Gendered input distribution as explanation?

*EthnicityEstimator.* Out of the tested NECs, EthnicityEstimator has the smallest average deviation of sensitivities between the genders (Fig. 4a). Its largest gender gap is 6 percentage points for ‘Other White’ (45%**female vs. ${51\%}^{ns}$ male). This is in line with expectations, as the Census represents the entire UK population, and is, therefore, gender-balanced. EthnicityEstimator works better for men in the Global North categories (British, Irish, Other White) and slightly better for women in the Global South categories (Indian, Pakistani, African). Thus, it is a reflection of what we would expect based on naming conventions.

*NamePrism*. This NEC displays major differences between the genders, with gaps as large as 16% points (Arabic ${63\%}^{ns}$ female vs. 79%***male and Scandinavian 38%***female vs. 54%***male). Twitter’s global gender split is 62% men and 38% women (Sehl 2020, p. 1). This figure varies starkly between countries. In the US and Latin America it is nearly 50%–50% (Wojcik and Hughes 2019, p. 1). This fits with ‘Anglo-American’ and ‘Hispanic’ being the only categories in NamePrism that work slightly better for women. With 86% of users being men, the highest male ratios on Twitter are found in Gambia, Niger and Congo (Sehl 2020, p. 1). This might be reflected in NamePrism’s large gender gap in the category ‘African’ (30%***female vs. 41%***male).

Furthermore, not only the distribution of users, but also the way users relate to each other on Twitter follows gendered patterns. Men tend to interact amongst each other more than with the other gender and vice versa (Ye and Skiena 2019, p. 2). Since the training of NamePrism rests on interaction networks (ibid.), it can be assumed that this ‘gender homophily’ further exacerbates the men-skew in male-dominated user landscapes.

As a result, NamePrism ends up displaying gender biases exactly opposed to EthnicityEstimator. For instance, in the ‘Anglo-American’ categories the census-based model performs better for men (67%***female vs. 68%***male), whereas the Twitter-based model performs better for women (81%***female vs. 80%***male). In the category ‘African’, relying on the Census leads to a higher sensitivity for women (39.1%***female vs.38.7%*** male), whereas relying on Twitter leads to a higher sensitivity for men (30%***female vs. 41%***male). NamePrism’s gender bias going in contrast to what we would expect based on naming conventions shows that for this NEC the gendered input distribution of names is the more relevant driver of its major gender gap.

*Ethnicolr*. Ethnicolr displays the largest gender gap of all three NECs, with the differences between sensitivities reaching up to 22% points for British (48%***female vs. 70%***male). This large skew in favour of men might be related to a large gender imbalance in Wikipedia entries. Out of all biographies on the platform, only 17% are on women (Shih 2017, p. 1). Nevertheless, there are three exceptions in which Ethnicolr works better for women: Indian (60%***female vs. ${47\%}^{ns}$ male), African (36%**female vs. 32%***male) and East European (${40\%}^{ns}$ female vs. 36%***male). These ‘female-better’ sensitivities are not attributable to more entries on women in these regions. On the contrary, with only 15% biographies on women, Africa is even below the average (Konieczny and Klein 2018, p. 4617). Instead, they are the result of naming conventions. This is an indicator that the ‘raw material’ can be influential enough to mitigate a distorted input source.

#### Age bias

##### Age bias as expected?

Given the ‘generational power gap’ arising from the numeric power of large Baby Boomer cohorts as well as from structural power within a neoliberalising global economic order (Frischmann 2005, p. 457, Hoffower 2021, p. 1), we might expect AIs to work better for older generations than younger ones. Indeed, NECs have been demonstrated to skew towards the elderly. Schnell et al. find that their name-based sampling technique is most likely to miss young migrants aged 15–22 years (2014, p. 241). Kandt and Longley show that Onomap’s accuracy increases from 47% for under 20-year-olds to 70% for over 70 year-olds (2018, p. 8).

**Fig. 5:**
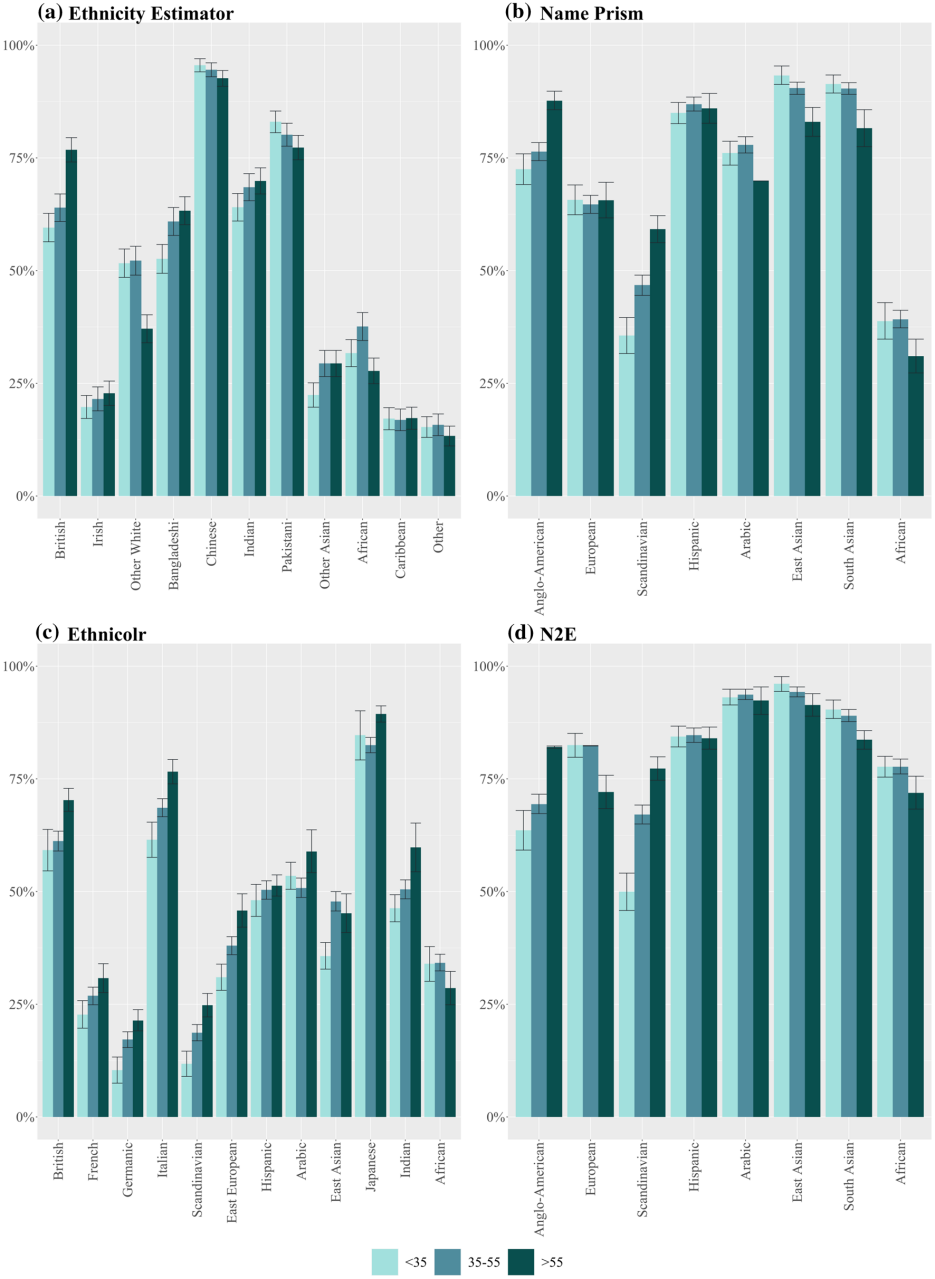
Sensitivity by age

In our intersectional analysis, the ‘old-over-young’ pattern turns out to be mostly accurate for Global North categories, and the opposite for Global South categories (see Fig. 5a–d). Out of 31 ethnicity categories, 21 have higher and 10 have lower sensitivities for those aged over 55 years than those aged under 35 years. Strikingly, amongst the ten ‘younger-better’ categories, none is from the Global North (only exception: ‘Other White’ in EthnicityEstimator, but this is a category that comprises of only non-British immigrants). High differences in sensitivities for the younger and the older groups can be discerned in the ‘older-better’ categories (e.g. Ethnicity Estimator: British 17% points; NamePrism: Anglo-American 15% points; Scandinavian 24% points) as well as, to a lesser degree, in the ‘younger-better’ categories (e.g. NamePrism: South Asian and East Asian both 10% points; EthnicityEstimator: Pakistani 6% points). Again, naming conventions and the input distribution intersect to produce this ‘Global North older better’ vs. ‘Global South younger better’ tendency.

##### Age-specific naming conventions as explanation?

When naming their offspring, most parents try to find a unique name. Paradoxically, it is exactly that desire for differentiation through which they often turn out to have been part of a fashion trend (Ainiala and Östman 2017, p. 11). Owing to the desire for novelty naming systems are not static, but dynamic (Hahn and Bentley 2003, p. 122). But their dynamism varies between cultures. Therefore, naming systems’ evolution might hold clues for explaining the Global South/Global North divide in age-dependent NEC performance.

First, post-migration name assimilation has a generational time component to it. Given that first-generation newcomers take their home-country names with them, little name assimilation can be expected in the first decades of an immigration wave. For the following generations, however, rates of name assimilation and inter-ethnic marriage accelerate. In the EthnicityEstimator’s UK dataset those belonging to the second or third generation of earlier waves of immigration are the younger ones. We can therefore expect decreasing accuracies for younger generations in the categories that have a long immigration history, but not in those that started immigrating only recently.

Figure 5a shows that this is to some extent the case. All ‘older-better’ categories are Commonwealth countries from which the first waves of ‘mass’ immigration originated. Indians (64%*** under 35 vs. 70%*** over 55) and Bangladeshis (53%*** under 35 vs. 63%*** over 55) both started arriving in the 1950s (Conway 2012, pp. 70–73). In contrast, the category that has the strongest ‘younger-better’ tilt is ‘Other White’ (52%* under 35 vs. 37%*** over 55), which came in significant numbers after the 2004 EU east-enlargement (ONS 2016). However, other categories, such as Pakistanis and Caribbeans do not fit with this picture since their accuracies are also decreasing with age despite their immigration histories dating back to the 1950s (Werbner 2005, p. 476). Given these inconsistencies, we should investigate once again whether pre-migration naming systems offer better explanations.

Second, the speed of naming systems’ change. Change occurs in most naming systems, from the Anglosphere—such as the shift from seventeenth century English names characteristic for the Puritanical era, like Faith and Patience, to nineteenth century floral and gem names, like Daisy and Ruby (Hanks and Hodges 1990, pp. xxii)—to the Middle East, from names like Islām and Fārūq, signifying virtue and piety, to names like Hanān and Sārra associated with love and happiness (Aouda and Shousha 1991, p. 164). Even though the underlying shift in values might be comparable, its speed and degree vary vastly. In the Arabic-speaking world, traditional names are still the norm. In fact, the most popular names have been the same from the early days of Islam, through the Middle Ages up to today: Muhammad, Ahmad, Ibrāhīm (Gardner 1994, p. 103).

Onomastic historians relate the speed of change to the values attached to tradition, on the one hand, and innovation on the other (Dunkling 1991, p. 52). This explanation might be embedded in the broader context of systems theories that investigate the social dynamics of innovation in different societies, such as Lévi-Strauss’ ‘cold societies’ (preserve their internal state, little ‘progress’) and ‘hot societies’ (internalise change, ‘greed for change’) (Maršálek 2020, p. 140). The distribution of ‘hot’ and ‘cold’ societies has a geographical component to it, with ‘coldness’ being preserved more in the Global South and ‘heat’ located in the Global North (Rehbein 2015, p. 54). As the evolution of names is a function of the way societies deal with change, it might be hypothesised that names in the Global North change faster than in the Global South. This would mean that the sensitivities for Global Southern categories are more stable throughout the age groups. As detailed above, the differences in percentage points are indeed smaller amongst Global South than Global North categories. But whereas the rate of change might explain age-accuracy stability, it cannot fully explain age-accuracy fluctuation. Each generation might have an overhauled, but still equally well distinguishable name pool. Therefore, the *kind* of change we see in naming systems is decisive.

Third, an increasing number of names. Onomasticians unanimously find that in all change-embracing naming systems the quantity of names increases through a greater diversity in names along with a smaller share of the most frequently used names (Ainiala and Östman 2017, p. 78; Gardner 1994, p. 188; Kællerød and Revuelta-Eugercios 2015, p. 75). For instance, a century ago, every eighth girl born in France was called Marie; today it is less than one in a hundred (The Economist 2019).

The reasons for this diversification might lie in the waning influence of religious restrictions, such as the Catholic church’s decree that all new-borns should be named after a saint (Fourquet 2019, p. 150). Apart from de-christianisation, de-collectivisation might be a further driver. In the past, instead of the concept of ‘identity’, the concept of ‘community’ was the basis of name-giving. This was reflected in personal names being largely the same within a clan, family or tribe (Kotilainen 2011, p. 52). Today’s individualisation, in contrast, drives differentiation in names within a group, and thus an ever-increasing need for novel names. As the role of religion as well as the role of the family has eroded faster in the Global North, we can expect that increasing heterogeneity makes names of younger people of this part of the world less recognisable for AIs. The NEC audit shows that this is the case, as all Global North categories’ (except ‘Other White’ in EthnicityEstimator) sensitivities decrease with decreasing age.

Fourth, the internationalisation of names. The incorporation of ‘innovative’ names has a geographic component to it. The most-travelled rout of names departs from the Anglosphere. Researchers attribute the Anglicisation of names to parents’ desire to express their modern, cosmopolitan lifestyle (Koß 2002, p. 116; Ainiala and Östman 2017, p. 54), or to their veneration of Anglo-American celebrities (Marzo and Zenner 2015, p. 10). Whereas the parents of little Britney and Beyoncé now getting their way within a once restrictive French naming legislation is a novelty (The Economist 2019), the phenomenon per se is not without historic precedent. Names used to ‘flow down’ from the nobility, the ‘celebrities’ of their time, to the bourgeoisie (Kællerød and Revuelta-Eugercios 2015, p. 74). Then as well as now this name mimicking has a power dimension to it: The less powerful try to resemble the powerful.

From a global geographic perspective, this would indicate a spread of names from the Global North to the Global South. Indeed, with names like Precious and Princess ranking amongst the most popular names in some African countries, this seems to be the case (Businesstech 2016). However, onomasticians find this phenomenon to a larger extent within the Global North’s ‘sub-centres’. Contact theory might help to explain this intra-regionalism. The theory posits that lexical borrowing occurs more where more contacts between language-spheres take place (Marzo and Zenner 2015, p. 8). Even though in the age of the Internet virtual contacts can take place independent from geography, contacts in the form of travel or a shared media landscape are still more intense within the greater proximity of Global Northern regions. Furthermore, cultural affinity is a prerequisite of ‘name borrowing’. It is thus unsurprising that the Anglicisation of names does not spread beyond the Global North to the cultural areas more opposed to Anglo-American values, such as Arabic countries in the Global South.

With Anglicisation being a source of confusion for NECs, it adds a further explanatory dimension to why youngsters in a more intertwined and Anglicised Global North are less easily classifiable than their progenitors. To conclude, age-specific naming conventions offer hints for why Global Northern categories follow the ‘older-better’ pattern, but not why Global South categories tend to be ‘younger-better’. Therefore, we need to dig into NECs’ naming input distributions.

##### Age-specific input distribution as explanation?

*EthnicityEstimator.* As half (5) of EthnicityEstimator’s categories work better for younger age groups and half (5) for older age groups, it is the most balanced of the three tested NECs with regards to the number of categories in each direction. However, within the ‘younger-better’ as well as the ‘older-better’-camp there are large percentage point differences in sensitivities. For instance, Brits over 55 are classified 17 percentage points more accurately than Brits under 35 and ‘Other Whites’ over 55 are classified 15 percentage points less accurately than those under 35.

EthnicityEstimator’s name input distribution is determined by the demographic of the UK’s population. The UK is an aging society, with one in five people being of retirement age (ONS 2019). The larger numbers of training data names from the elderly might explain the increasing accuracy for natives (i.e. the category British, and partially also Irish). Immigrants, in contrast, are on average much younger. Only one in ten immigrants is of retirement age. 70% of the foreign born are of working age, compared to 48% of the UK born (Vargas-Silva and Rienzo 2022, p. 4). Immigrants’ younger demographic has manifold reasons: increased migration is a recent phenomenon and young immigrants have not grown old yet; higher fertility amongst immigrants (Waller et al. 2014, p. 131); and circular migration in which people move back to their home country after having spent (part of) their working live in the UK (Joxhe 2018, p. 197).

However, this aggregate picture is not equally the case for all immigrant groups. It is most accurate for the EU-8 countries were the largest percentage (15%) of young people aged 16–25 originating from. This fits well with EthnicityEstimator’s large ‘younger-better’ tilt in the ‘Other White’ category. The other ‘younger-better’ categories—Chinese, Pakistani, African and Caribbean–are also listed amongst those with the youngest demographics (Vargas-Silva and Rienzo 2022, p. 5). The age statistics also provide a good explanation for why not every immigrant category falls into the ‘younger-better’ camp. The smallest percentage (5%) of people aged 16–25 are born in India, which traces through to 6% points less sensitivity for young Indians compared to older Indians.

To conclude, EthnicityEstimator’s age input distribution correlates significantly with its sensitivity rates. Its input bias therefore seems like a relevant explanatory factor for its age-specific biases. With regards to Global North categories, its ‘older-better’ tilt is in line with the hypothesis derived from naming conventions as well as from its input distribution. With regards to Global South categories, however, scrutinising the input distribution fills a gap that we were not able to predict based on naming conventions alone, namely why some Global South categories perform better for the younger generation. Due to EthnicityEstimator’s reliance on a post-migration training dataset the reason for the ‘younger-better’ tendencies of this NEC lies in the dynamics of migration (self)selection.

*NamePrism.* Figure 5b shows NamePrism’s clear-cut distinction between Global South and Global North categories. The sensitivity bar charts for all three Global North categories rise upwards with age (with increases as high as 15% points for Anglo-American and 24% points for Scandinavian), while the bars for the remaining five Global South categories diminish with age (up to 10% points for East Asian, South Asian and African). NamePrism’s input database, Twitter, is a young platform, with 62% of users being under 35 (Statista 2021). Twitter does not share a split-up of its age distribution into nationalities. It might be assumed that it loosely follows the age demographics of each country. With Global South nations having a younger population, it seems probable that the NamePrism crawl of Twitter scraped more names of younger users, thus gaining a better training database for this age group in the Global South. In the Global North, Twitter users might be older. However, the patchy evidence that exists suggests that in the US still 48% of users are under 35 (Omnicore 2022) and a crawl of Twitter in the UK suggests that 95% are under 35 (Sloan et al. 2015, p. 15). The latter figure might be exaggerated, but it still indicates that even in the Global North Twitter offers substantially better training data for younger cohorts than for older ones.

In conclusion, NamePrism’s age-related input bias might well explain the Global South’s ‘younger-better’, but not the Global North’s ‘old better’ tendency. The greater naming uniformity of older generations in the Global North is such a strong influence on AI performance that it offsets Twitter’s age demographics.

*Ethnicolr. *Except for Africa, Ethnicolr works better for older people in all categories. The sensitivity differences between the youngest and the oldest age group reach up to 20% points for East European, and 21% points for Arabic. For the remaining four Global South categories the difference is slightly smaller (e.g. 14% points for Indian, 11% points for East Asian).

Ethnicolr’s age input bias hinges on who receives a Wikipedia biography entry. This is regulated by Wikipedia’s ‘notability’ policy, which ensures that entries on people not relevant enough will be deleted (Graham 2015, p. 229). The number of personalities being deemed ‘notable’ rises as we move closer to modern times, but then decreases significantly in the last forty decades (Konieczny and Klein 2018, pp. 4615–4619). It takes a good chunk of one’s lifespan to become ‘noticeable’. This seems to be a universal phenomenon, given the ‘older-better’ sensitivities also in Global Southern categories where naming conventions would not suggest so. Just like for NamePrism, also for Ethnicolr input bias trumps naming conventions.

#### Bias reduction

**Fig. 6:**
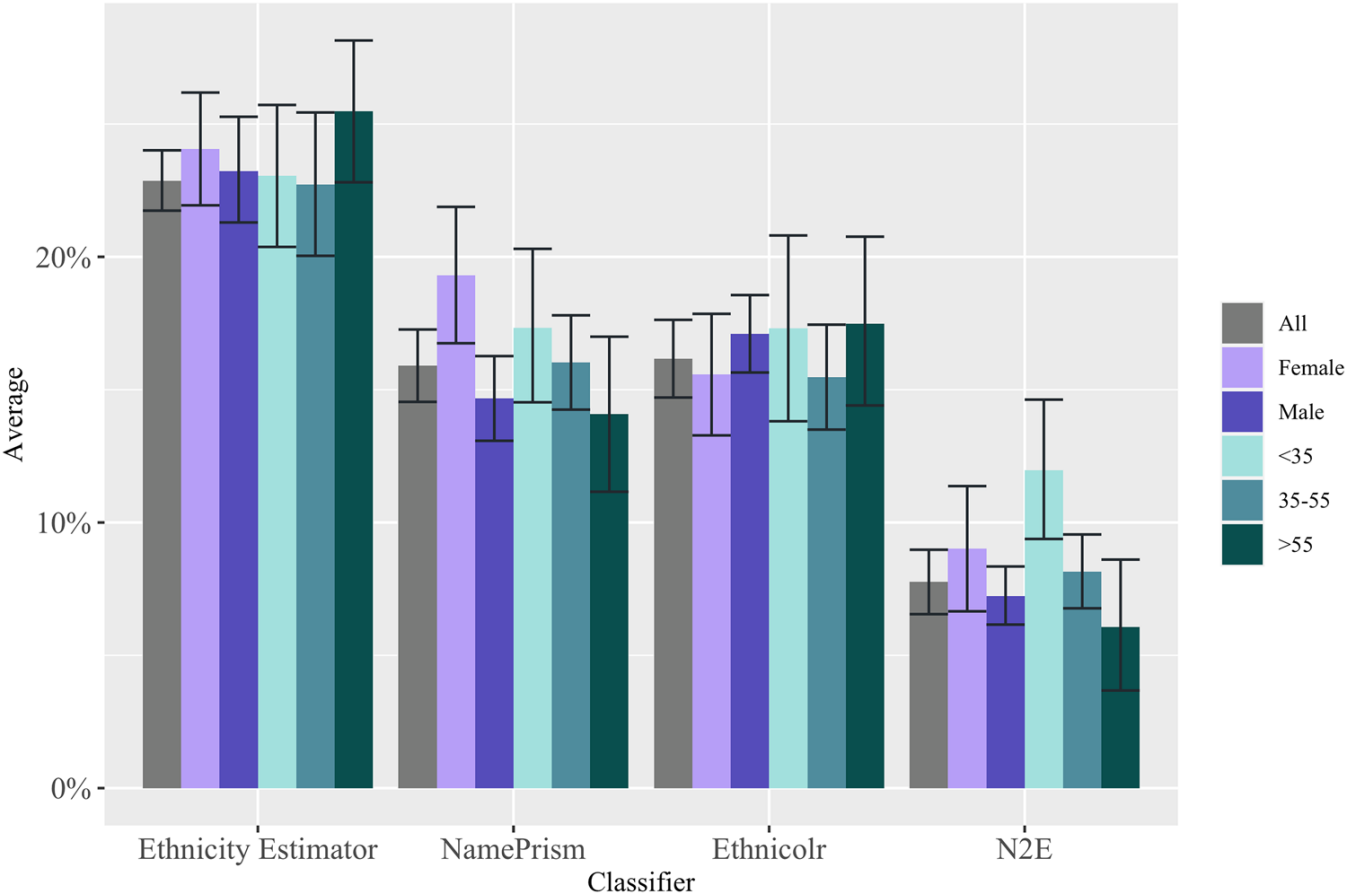
Average deviation of sensitivities

To improve on the uncovered biases, we developed N2E through the fairness-aware AI design described in the methodology section. Figure 6 shows that this endeavour was successful, measured by reduced average deviations of sensitivities. Figure 6 illustrates that each NEC has similar average deviations along ethnic, gender and age dimensions. Overlapping confidence intervals indicate that the differences within NECs are not statistically reliable, except for the gender dimension in NamePrism and age in N2E. In other words, within-NEC differences in biases are small, but between-NEC differences are significant. This is the case as ethnicity bias sets the ‘baseline’. Once it is off, its bias traces through to intra-ethnic gender and age biases.

Therefore, getting ethnicity ‘right’ first was crucial for N2E’s low average deviation in sensitivities throughout all bias types. With only 8%*** of deviation between the ethnicity categories—compared to 23%*** (EthnicityEstimator), 16%*** (NamePrism) and 16%*** (Ethnicolr)—this is the bias class in which we achieved the greatest advancements. This reduction in inequality is coupled with a boost in sensitivities. The least well-classified category is ‘Scandinavian’, which still has 68%*** sensitivity; the most accurately classified categories are ‘East Asian’ with 94%*** and ‘Arabic’ with 93%*** (see Fig. 3d). The category that best epitomises this ‘a rising tide lifts all boats’ pattern of N2E’s bias reduction is ‘African’. By identifying only 33*** (Ethnicolr), 37*** (EthnicityEstimator) and 38*** (NamePrism) out of 100 Africans, all tested NECs performed consistently poorly (see Fig. 3a–c). N2E raises this figure to 77*** out of 100.

With regards to gender bias, N2E’s average deviation for females is 9%*** and for males 7%*** (see Fig. 6). Thus, it remains within AI’s tradition of working better for men. N2E only works equally well for both genders in the categories ‘African’ and ‘Hispanic’. Given that these are both Global South categories this fits with the differences in naming conventions established above. In its remaining six categories the differences in sensitivities are up to 8% points (Scandinavian 62%***female vs. 70%***male), but on average ‘only’ 4–5% points (see Fig. 4d). In consequence, out of the tested NECs, N2E still has the smallest average deviation between the genders.

In terms of age bias, N2E’s downward-facing staircase of average deviation in sensitivities of 12%*** for those under 35 years, 8%*** for 35–55 year-olds, and 6%*** for over 55 year-olds, signals that the tool’s bias gets smaller with age (see Fig. 6). N2E has the largest tilt for ‘older-better’ in the categories ‘Anglo-American’ (64%*** for under 35 vs. ${82\%}^{ns}$ for over 55) and ‘Scandinavian’ (50%*** for under 35 vs. 77%*** for over 55). Both being Global North categories, this is in line with the hypothesis derived from naming conventions. In the remaining categories, its performance slightly decreases with age. Given N2E’s ‘headstart’ in ethnicity bias, its average deviations in sensitivities between the age groups are still smaller than those of the other tested NECs. Nevertheless, ‘age’ bias saw the smallest improvement from our fairness-aware approach.

The performance across all bias classes might be tweaked slightly upwards as AI technology improves and further fairness-enhancers get invented. However, with data input being the limiting factor, substantial improvements are only to be expected with an even larger training database. But since this needs to be annotated with ethnicity/nationality, gender as well as age, such a dataset would be a rare gem of whose existence we are unaware. Furthermore, as the training of N2E on a balanced dataset still resulted in manifold biases, naming conventions could prove to be a ceiling that all NECs hit at some point. We therefore invite researchers to refer to this paper for being transparent about the biases that get imported into their research if relying on NECs, and to use N2E (freely available at www.name-to-ethnicity.com) for a state-of-the-art classifier designed to reduce these biases.

## Conclusion

We offered a fairness audit of algorithms that infer ethnicity from names. We took a broad approach by first questioning the ethical use of NECs, concluding that their potential ethical risks are outweighed by their contribution to uncovering ethnic inequalities. Subsequently, we scrutinised the fairness of three NECs—the UK-census trained EthnicityEstimator, Twitter-trained NamePrism and Wikipedia-trained Ethnicolr—by measuring imbalances in their sensitivities along the axes of ethnicity, gender and age.

To investigate the origins of the uncovered biases, we disentangled the two dimensions that characterise NECs’ input data as follows: naming conventions and the distribution of names. Both dimensions turned out to display distinctions along the heuristic line of Global South | Global North. For instance, with regards to naming conventions, peripheral regions copy names typical in the dominant regions, to assimilate to global power centres. With regards to the distribution of names, lack of access (Twitter), representation (Wikipedia) or being a minority (UK Census) leads to people from the Global South being underrepresented in the training data. Therefore, NECs, just like most AIs, are heavily infused by global power structures.

The concrete ways in which these power structures trace through to differences in accuracy rates varies. The audit showed that whereas EthnicityEstimator has large differences in sensitivities amongst ethnic categories, its biases with regards to gender and age are relatively smaller. In contrast, the other two NECs have less variation among ethnic categories, but relatively more among gender and age groups. Also, how the two dimensions of naming conventions and input distribution interact differs by NEC. In some aspects, naming conventions overwrite input bias (e.g. for age sensitivities in NamePrism and Ethnicolr). In others, the input bias is stronger than naming conventions (e.g. for gendered sensitivities in NamePrism).

Lastly, we introduced N2E, an NEC designed with the goal of bias reduction. To improve on the tested NECs’ bias-prone input data sampled ‘in the wild’, we assembled training data ‘in the lab’. To mitigate bias in input distribution, we equalised the training data through down-sampling. To mitigate bias in naming conventions, we engaged in the synthetic name production. As these techniques succeeded in reducing bias, we invite the research community to use N2E (freely available on www.name-to-ethnicity.com) to uncover the word’s ethnic injustices more reliably.

We also disclosed which biases remain. This will enable researchers to be more transparent about potential flaws imported into their scholarship. Furthermore, we hope that the bias-relevant information about naming conventions and input distributions assembled in this paper might provide AI coders with a starting point to develop even fairer NECs in the future. Then,

**Table:** 

Andrew Smith	from ———————— and
Abubakar Shabalala	from ————————

will have equally high chances to be classified correctly.

## Supplementary Information

Below is the link to the electronic supplementary material.

Supplementary file1 (DOCX 182 KB)

## Data Availability

The datasets generated and/or analysed during the current study are available in the following repositories: Python code for N2E on Github: https://github.com/name-ethnicity-classifier/name-ethnicity-classifier. Training dataset for N2E on Companies House: http://download.companieshouse.gov.uk/en_output.html. N2E: https://www.name-to-ethnicity.com/. EthnicityEstimator: https://data.cdrc.ac.uk/ethnicity_estimator. NamePrism: https://www.name-prism.com/. Ethnicolr: https://Ethnicolr.readthedocs.io/Ethnicolr.html#underlying-data.

## Footnotes

1 For a good overview of NECs developed up to 2007, see Mateo 2007.

2 *NamSor.* https://namsor.app/.

3 *OriginsInfo.* https://www.originsinfo.eu/.

4 For a comprehensive survey of bias mitigation methods see Hort et al. (2022).

5 *NamePrism*. https://www.name-prism.com/.

6 *EthnicityEstimator.* https://data.cdrc.ac.uk/ethnicity_estimator.

7 *Ethnicolr.* https://Eethnicolr.readthedocs.io/ethnicolr.html#underlying-data.

8 For reviews of definitions of bias and fairness in AI see Gajane and Pechenizkiy (2018), Hellström et al. (2020) and Besse et al. (2022).

9 For a survey of fairness metrics see Verma and Rubin (2018) and Pagano et al. (2022).

10 ns for *p* > 0.05, * for *p* ≤ 0.05, ** for *p* ≤ 0.01 and *** for *p* ≤ 0.001.

11 *CompaniesHouse*. https://www.gov.uk/government/organisations/companies-house.

12 N2E code on GitHuB: https://github.com/name-ethnicity-classifier.
