# Supplementary material for: Equal accuracy for Andrew and Abubakar—detecting and mitigating bias in name-ethnicity classification algorithms
Source: AI Soc. 2023 Feb 9:1–25. Online ahead of print. doi: 10.1007/s00146-022-01619-4 (PMC9910274; doi:10.1007/s00146-022-01619-4)
Supplement: Supplementary file 1 — Supplementary file1 (DOCX 182 KB) [file 146_2022_1619_MOESM1_ESM.docx]

**Appendix**

Name Prism

All

|  | Sensitivity | | | Specificity | | | Positive Predictive Value | | | Negative Predictive Value | | | Precision | | |
| --- | --- | --- | --- | --- | --- | --- | --- | --- | --- | --- | --- | --- | --- | --- | --- |
|  | Value | P | CI | Value | P | CI | Value | P | CI | Value | P | CI | Value | P | CI |
| African | 0.378 | <.001 | 0.362-0.394 | 0.991 | <.001 | 0.987-0.995 | 0.833 | <.001 | 0.820-0.846 | 0.934 | <.001 | 0.926-0.942 | 0.833 | <.001 | 0.820-0.846 |
| Anglo-American | 0.803 | <.001 | 0.790-0.816 | 0.814 | <.001 | 0.802-0.826 | 0.325 | <.001 | 0.309-0.340 | 0.973 | 0.0447 | 0.968-0.979 | 0.325 | <.001 | 0.309-0.340 |
| Arabic | 0.766 | <.001 | 0.752-0.780 | 0.959 | >.1 | 0.956-0.961 | 0.676 | <.001 | 0.660-0.691 | 0.973 | 0.0538 | 0.968-0.979 | 0.676 | <.001 | 0.660-0.691 |
| East Asian | 0.899 | <.001 | 0.888-0.910 | 0.975 | <.001 | 0.970-0.980 | 0.803 | <.001 | 0.790-0.817 | 0.988 | <.001 | 0.984-0.992 | 0.803 | <.001 | 0.790-0.817 |
| European | 0.651 | <.001 | 0.635-0.667 | 0.928 | <.001 | 0.920-0.937 | 0.503 | <.001 | 0.487-0.520 | 0.959 | 0.0013 | 0.953-0.966 | 0.503 | <.001 | 0.487-0.520 |
| Hispanic | 0.862 | <.001 | 0.850-0.874 | 0.964 | 0.0137 | 0.958-0.970 | 0.73 | <.001 | 0.715-0.745 | 0.984 | <.001 | 0.980-0.988 | 0.73 | <.001 | 0.715-0.745 |
| Scandinavian | 0.49 | <.001 | 0.473-0.506 | 0.998 | <.001 | 0.996-1.001 | 0.981 | <.001 | 0.974-0.988 | 0.946 | <.001 | 0.938-0.953 | 0.981 | <.001 | 0.974-0.988 |
| South Asian | 0.898 | <.001 | 0.888-0.909 | 0.941 | <.001 | 0.934-0.949 | 0.631 | 0.0242 | 0.615-0.647 | 0.988 | <.001 | 0.984-0.992 | 0.631 | 0.0242 | 0.615-0.647 |

|  | F1 | | | Prevalence | | | Detection Rate | | | Detection Prevalence | | | Balanced Accuracy | | |
| --- | --- | --- | --- | --- | --- | --- | --- | --- | --- | --- | --- | --- | --- | --- | --- |
|  | Value | P | CI | Value | P | CI | Value | P | CI | Value | P | CI | Value | P | CI |
| African | 0.52 | <.001 | 0.504-0.537 | 0.1 | 0.0214 | 0.090-0.109 | 0.037 | <.001 | 0.031-0.044 | 0.045 | <.001 | 0.038-0.052 | 0.685 | <.001 | 0.670-0.700 |
| Anglo-American | 0.462 | <.001 | 0.446-0.479 | 0.1 | 0.0214 | 0.090-0.109 | 0.08 | <.001 | 0.071-0.089 | 0.247 | <.001 | 0.233-0.260 | 0.809 | <.001 | 0.796-0.821 |
| Arabic | 0.718 | <.001 | 0.703-0.733 | 0.1 | 0.0214 | 0.090-0.109 | 0.076 | <.001 | 0.067-0.085 | 0.113 | 0.0032 | 0.102-0.123 | 0.862 | <.001 | 0.851-0.874 |
| East Asian | 0.848 | <.001 | 0.836-0.861 | 0.1 | 0.0214 | 0.090-0.109 | 0.089 | <.001 | 0.080-0.099 | 0.111 | 0.0087 | 0.101-0.122 | 0.937 | <.001 | 0.928-0.946 |
| European | 0.568 | 0.0161 | 0.551-0.584 | 0.1 | 0.0214 | 0.090-0.109 | 0.065 | >.1 | 0.060-0.069 | 0.129 | <.001 | 0.118-0.140 | 0.79 | <.001 | 0.776-0.803 |
| Hispanic | 0.791 | <.001 | 0.777-0.804 | 0.1 | 0.0214 | 0.090-0.109 | 0.086 | <.001 | 0.077-0.095 | 0.118 | <.001 | 0.107-0.128 | 0.913 | <.001 | 0.904-0.923 |
| Scandinavian | 0.653 | <.001 | 0.637-0.669 | 0.1 | 0.0214 | 0.090-0.109 | 0.049 | <.001 | 0.041-0.056 | 0.049 | <.001 | 0.042-0.057 | 0.744 | <.001 | 0.730-0.758 |
| South Asian | 0.742 | <.001 | 0.727-0.756 | 0.099 | 0.0218 | 0.090-0.109 | 0.089 | <.001 | 0.080-0.099 | 0.142 | <.001 | 0.130-0.153 | 0.92 | <.001 | 0.910-0.929 |

Female

|  | Sensitivity | | | Specificity | | | Positive Predictive Value | | | Negative Predictive Value | | | Precision | | |
| --- | --- | --- | --- | --- | --- | --- | --- | --- | --- | --- | --- | --- | --- | --- | --- |
|  | Value | P | CI | Value | P | CI | Value | P | CI | Value | P | CI | Value | P | CI |
| African | 0.304 | <.001 | 0.277-0.331 | 0.996 | <.001 | 0.990-1.001 | 0.907 | <.001 | 0.888-0.926 | 0.92 | <.001 | 0.905-0.936 | 0.907 | <.001 | 0.888-0.926 |
| Anglo-American | 0.808 | <.001 | 0.783-0.833 | 0.767 | <.001 | 0.742-0.793 | 0.264 | <.001 | 0.236-0.292 | 0.974 | >.1 | 0.966-0.983 | 0.264 | <.001 | 0.236-0.292 |
| Arabic | 0.633 | 0.0905 | 0.600-0.666 | 0.981 | 0.001 | 0.969-0.993 | 0.657 | >.1 | 0.652-0.662 | 0.979 | 0.0832 | 0.966-0.991 | 0.657 | >.1 | 0.652-0.662 |
| East Asian | 0.872 | <.001 | 0.853-0.890 | 0.973 | <.001 | 0.965-0.982 | 0.859 | <.001 | 0.840-0.878 | 0.976 | 0.0229 | 0.968-0.984 | 0.859 | <.001 | 0.840-0.878 |
| European | 0.573 | <.001 | 0.540-0.605 | 0.932 | <.001 | 0.915-0.949 | 0.435 | <.001 | 0.402-0.467 | 0.96 | >.1 | 0.952-0.967 | 0.435 | <.001 | 0.402-0.467 |
| Hispanic | 0.876 | <.001 | 0.857-0.895 | 0.951 | >.1 | 0.946-0.955 | 0.746 | <.001 | 0.723-0.769 | 0.979 | 0.0051 | 0.970-0.987 | 0.746 | <.001 | 0.723-0.769 |
| Scandinavian | 0.381 | <.001 | 0.352-0.410 | 0.999 | <.001 | 0.994-1.004 | 0.98 | <.001 | 0.968-0.993 | 0.934 | <.001 | 0.919-0.949 | 0.98 | <.001 | 0.968-0.993 |
| South Asian | 0.88 | <.001 | 0.858-0.901 | 0.952 | >.1 | 0.950-0.955 | 0.674 | >.1 | 0.650-0.698 | 0.986 | <.001 | 0.978-0.994 | 0.674 | >.1 | 0.650-0.698 |

|  | F1 | | | Prevalence | | | Detection Rate | | | Detection Prevalence | | | Balanced Accuracy | | |
| --- | --- | --- | --- | --- | --- | --- | --- | --- | --- | --- | --- | --- | --- | --- | --- |
|  | Value | P | CI | Value | P | CI | Value | P | CI | Value | P | CI | Value | P | CI |
| African | 0.456 | <.001 | 0.427-0.485 | 0.109 | 0.0103 | 0.091-0.128 | 0.033 | <.001 | 0.022-0.044 | 0.036 | <.001 | 0.024-0.049 | 0.65 | <.001 | 0.623-0.677 |
| Anglo-American | 0.399 | <.001 | 0.368-0.429 | 0.093 | >.1 | 0.089-0.098 | 0.075 | 0.0184 | 0.059-0.092 | 0.286 | <.001 | 0.258-0.314 | 0.788 | 0.0634 | 0.764-0.811 |
| Arabic | 0.645 | <.001 | 0.605-0.684 | 0.053 | <.001 | 0.034-0.073 | 0.034 | 0.0051 | 0.018-0.049 | 0.051 | <.001 | 0.032-0.070 | 0.807 | >.1 | 0.805-0.809 |
| East Asian | 0.865 | <.001 | 0.847-0.884 | 0.155 | <.001 | 0.137-0.172 | 0.135 | <.001 | 0.118-0.151 | 0.157 | <.001 | 0.139-0.175 | 0.923 | <.001 | 0.908-0.937 |
| European | 0.494 | <.001 | 0.461-0.527 | 0.083 | >.1 | 0.075-0.090 | 0.047 | >.1 | 0.034-0.060 | 0.109 | >.1 | 0.098-0.120 | 0.752 | <.001 | 0.724-0.781 |
| Hispanic | 0.806 | <.001 | 0.784-0.827 | 0.141 | <.001 | 0.123-0.159 | 0.123 | <.001 | 0.106-0.140 | 0.165 | <.001 | 0.146-0.184 | 0.913 | <.001 | 0.897-0.929 |
| Scandinavian | 0.549 | >.1 | 0.549-0.549 | 0.102 | >.1 | 0.088-0.115 | 0.038 | 0.0019 | 0.026-0.051 | 0.039 | <.001 | 0.026-0.052 | 0.69 | <.001 | 0.662-0.718 |
| South Asian | 0.763 | <.001 | 0.737-0.790 | 0.1 | >.1 | 0.088-0.111 | 0.088 | <.001 | 0.070-0.105 | 0.13 | <.001 | 0.109-0.151 | 0.916 | <.001 | 0.898-0.934 |

Male

|  | Sensitivity | | | Specificity | | | Positive Predictive Value | | | Negative Predictive Value | | | Precision | | |
| --- | --- | --- | --- | --- | --- | --- | --- | --- | --- | --- | --- | --- | --- | --- | --- |
|  | Value | P | CI | Value | P | CI | Value | P | CI | Value | P | CI | Value | P | CI |
| African | 0.413 | <.001 | 0.394-0.433 | 0.989 | <.001 | 0.984-0.994 | 0.81 | <.001 | 0.793-0.826 | 0.94 | <.001 | 0.931-0.950 | 0.81 | <.001 | 0.793-0.826 |
| Anglo-American | 0.801 | <.001 | 0.785-0.817 | 0.834 | <.001 | 0.820-0.848 | 0.356 | <.001 | 0.337-0.374 | 0.973 | >.1 | 0.968-0.978 | 0.356 | <.001 | 0.337-0.374 |
| Arabic | 0.791 | <.001 | 0.776-0.806 | 0.949 | 0.0137 | 0.941-0.957 | 0.679 | <.001 | 0.662-0.696 | 0.971 | >.1 | 0.968-0.973 | 0.679 | <.001 | 0.662-0.696 |
| East Asian | 0.922 | <.001 | 0.909-0.935 | 0.976 | <.001 | 0.969-0.983 | 0.764 | <.001 | 0.745-0.783 | 0.993 | <.001 | 0.989-0.997 | 0.764 | <.001 | 0.745-0.783 |
| European | 0.676 | <.001 | 0.659-0.694 | 0.927 | <.001 | 0.917-0.936 | 0.526 | <.001 | 0.507-0.545 | 0.959 | 0.0026 | 0.952-0.967 | 0.526 | <.001 | 0.507-0.545 |
| Hispanic | 0.853 | <.001 | 0.837-0.868 | 0.969 | 0.0022 | 0.962-0.977 | 0.719 | <.001 | 0.700-0.738 | 0.986 | <.001 | 0.981-0.991 | 0.719 | <.001 | 0.700-0.738 |
| Scandinavian | 0.536 | <.001 | 0.517-0.555 | 0.998 | <.001 | 0.995-1.001 | 0.981 | <.001 | 0.973-0.989 | 0.951 | <.001 | 0.943-0.959 | 0.981 | <.001 | 0.973-0.989 |
| South Asian | 0.906 | <.001 | 0.894-0.918 | 0.937 | <.001 | 0.927-0.946 | 0.616 | <.001 | 0.597-0.635 | 0.989 | <.001 | 0.984-0.993 | 0.616 | <.001 | 0.597-0.635 |

|  | F1 | | | Prevalence | | | Detection Rate | | | Detection Prevalence | | | Balanced Accuracy | | |
| --- | --- | --- | --- | --- | --- | --- | --- | --- | --- | --- | --- | --- | --- | --- | --- |
|  | Value | P | CI | Value | P | CI | Value | P | CI | Value | P | CI | Value | P | CI |
| African | 0.547 | <.001 | 0.527-0.567 | 0.095 | >.1 | 0.089-0.102 | 0.039 | <.001 | 0.031-0.047 | 0.049 | <.001 | 0.039-0.058 | 0.701 | <.001 | 0.683-0.719 |
| Anglo-American | 0.493 | <.001 | 0.473-0.512 | 0.102 | 0.0148 | 0.090-0.114 | 0.082 | <.001 | 0.071-0.092 | 0.23 | <.001 | 0.214-0.246 | 0.817 | <.001 | 0.803-0.832 |
| Arabic | 0.731 | <.001 | 0.714-0.747 | 0.119 | <.001 | 0.107-0.130 | 0.094 | <.001 | 0.083-0.104 | 0.138 | <.001 | 0.126-0.151 | 0.87 | <.001 | 0.858-0.882 |
| East Asian | 0.835 | <.001 | 0.818-0.852 | 0.077 | 0.0266 | 0.065-0.089 | 0.071 | 0.0657 | 0.060-0.081 | 0.093 | >.1 | 0.085-0.100 | 0.949 | <.001 | 0.938-0.959 |
| European | 0.592 | >.1 | 0.585-0.598 | 0.107 | <.001 | 0.095-0.118 | 0.072 | 0.0141 | 0.062-0.082 | 0.137 | <.001 | 0.124-0.150 | 0.801 | <.001 | 0.786-0.817 |
| Hispanic | 0.78 | <.001 | 0.762-0.798 | 0.082 | >.1 | 0.074-0.090 | 0.07 | 0.0697 | 0.061-0.080 | 0.098 | >.1 | 0.096-0.100 | 0.911 | <.001 | 0.898-0.924 |
| Scandinavian | 0.693 | <.001 | 0.675-0.712 | 0.099 | 0.0846 | 0.089-0.109 | 0.053 | 0.0453 | 0.044-0.062 | 0.054 | <.001 | 0.044-0.063 | 0.767 | <.001 | 0.751-0.784 |
| South Asian | 0.733 | <.001 | 0.716-0.751 | 0.099 | 0.0578 | 0.088-0.110 | 0.09 | <.001 | 0.079-0.101 | 0.147 | <.001 | 0.133-0.160 | 0.921 | <.001 | 0.910-0.932 |

Under 35 Years

|  | Sensitivity | | | Specificity | | | Positive Predictive Value | | | Negative Predictive Value | | | Precision | | |
| --- | --- | --- | --- | --- | --- | --- | --- | --- | --- | --- | --- | --- | --- | --- | --- |
|  | Value | P | CI | Value | P | CI | Value | P | CI | Value | P | CI | Value | P | CI |
| African | 0.388 | <.001 | 0.348-0.429 | 0.991 | <.001 | 0.982-1.000 | 0.785 | <.001 | 0.750-0.819 | 0.952 | 0.0026 | 0.934-0.970 | 0.785 | <.001 | 0.750-0.819 |
| Anglo-American | 0.725 | >.1 | 0.691-0.759 | 0.845 | <.001 | 0.812-0.878 | 0.226 | <.001 | 0.187-0.266 | 0.98 | >.1 | 0.970-0.989 | 0.226 | <.001 | 0.187-0.266 |
| Arabic | 0.761 | <.001 | 0.734-0.787 | 0.947 | 0.0224 | 0.934-0.961 | 0.716 | <.001 | 0.688-0.744 | 0.957 | 0.004 | 0.945-0.970 | 0.716 | <.001 | 0.688-0.744 |
| East Asian | 0.933 | <.001 | 0.913-0.954 | 0.972 | 0.0632 | 0.960-0.984 | 0.799 | <.001 | 0.769-0.828 | 0.992 | <.001 | 0.984-0.999 | 0.799 | <.001 | 0.769-0.828 |
| European | 0.657 | 0.0113 | 0.624-0.690 | 0.941 | 0.0021 | 0.924-0.957 | 0.582 | <.001 | 0.548-0.616 | 0.956 | 0.0052 | 0.942-0.970 | 0.582 | <.001 | 0.548-0.616 |
| Hispanic | 0.85 | <.001 | 0.826-0.873 | 0.971 | 0.0496 | 0.960-0.982 | 0.833 | <.001 | 0.809-0.857 | 0.974 | >.1 | 0.971-0.978 | 0.833 | <.001 | 0.809-0.857 |
| Scandinavian | 0.356 | <.001 | 0.316-0.396 | 0.998 | <.001 | 0.992-1.005 | 0.96 | <.001 | 0.940-0.979 | 0.952 | 0.0029 | 0.934-0.970 | 0.96 | <.001 | 0.940-0.979 |
| South Asian | 0.914 | <.001 | 0.894-0.934 | 0.94 | <.001 | 0.925-0.955 | 0.702 | 0.0027 | 0.673-0.732 | 0.986 | 0.0029 | 0.977-0.994 | 0.702 | 0.0027 | 0.673-0.732 |

|  | F1 | | | Prevalence | | | Detection Rate | | | Detection Prevalence | | | Balanced Accuracy | | |
| --- | --- | --- | --- | --- | --- | --- | --- | --- | --- | --- | --- | --- | --- | --- | --- |
|  | Value | P | CI | Value | P | CI | Value | P | CI | Value | P | CI | Value | P | CI |
| African | 0.52 | <.001 | 0.478-0.561 | 0.074 | >.1 | 0.058-0.091 | 0.029 | <.001 | 0.013-0.044 | 0.037 | <.001 | 0.019-0.054 | 0.69 | <.001 | 0.652-0.728 |
| Anglo-American | 0.345 | <.001 | 0.301-0.390 | 0.058 | 0.012 | 0.036-0.081 | 0.042 | 0.0443 | 0.022-0.062 | 0.188 | <.001 | 0.151-0.224 | 0.785 | 0.0085 | 0.747-0.823 |
| Arabic | 0.738 | <.001 | 0.710-0.765 | 0.148 | <.001 | 0.127-0.169 | 0.112 | <.001 | 0.093-0.131 | 0.157 | <.001 | 0.135-0.179 | 0.854 | 0.0114 | 0.832-0.876 |
| East Asian | 0.861 | <.001 | 0.835-0.887 | 0.104 | >.1 | 0.088-0.120 | 0.097 | <.001 | 0.076-0.118 | 0.121 | 0.0274 | 0.098-0.145 | 0.953 | <.001 | 0.936-0.970 |
| European | 0.617 | 0.0209 | 0.583-0.651 | 0.11 | 0.0238 | 0.089-0.132 | 0.072 | >.1 | 0.063-0.082 | 0.125 | 0.0088 | 0.102-0.148 | 0.799 | 0.0194 | 0.771-0.827 |
| Hispanic | 0.841 | <.001 | 0.817-0.865 | 0.143 | <.001 | 0.122-0.164 | 0.122 | <.001 | 0.102-0.141 | 0.146 | <.001 | 0.125-0.168 | 0.91 | <.001 | 0.892-0.929 |
| Scandinavian | 0.519 | 0.0012 | 0.477-0.561 | 0.072 | >.1 | 0.052-0.091 | 0.025 | <.001 | 0.010-0.040 | 0.026 | <.001 | 0.011-0.042 | 0.677 | <.001 | 0.638-0.716 |
| South Asian | 0.795 | <.001 | 0.768-0.821 | 0.132 | <.001 | 0.111-0.153 | 0.121 | <.001 | 0.100-0.141 | 0.172 | <.001 | 0.148-0.196 | 0.927 | <.001 | 0.909-0.945 |

35-55 Years

|  | Sensitivity | | | Specificity | | | Positive Predictive Value | | | Negative Predictive Value | | | Precision | | |
| --- | --- | --- | --- | --- | --- | --- | --- | --- | --- | --- | --- | --- | --- | --- | --- |
|  | Value | P | CI | Value | P | CI | Value | P | CI | Value | P | CI | Value | P | CI |
| African | 0.392 | <.001 | 0.373-0.412 | 0.991 | <.001 | 0.986-0.996 | 0.856 | <.001 | 0.841-0.871 | 0.927 | <.001 | 0.916-0.937 | 0.856 | <.001 | 0.841-0.871 |
| Anglo-American | 0.764 | <.001 | 0.744-0.784 | 0.81 | <.001 | 0.792-0.828 | 0.265 | <.001 | 0.244-0.286 | 0.974 | >.1 | 0.968-0.981 | 0.265 | <.001 | 0.244-0.286 |
| Arabic | 0.779 | <.001 | 0.761-0.797 | 0.96 | >.1 | 0.955-0.965 | 0.689 | <.001 | 0.669-0.709 | 0.974 | 0.0782 | 0.968-0.981 | 0.689 | <.001 | 0.669-0.709 |
| East Asian | 0.905 | <.001 | 0.891-0.918 | 0.975 | <.001 | 0.968-0.982 | 0.813 | <.001 | 0.796-0.830 | 0.988 | <.001 | 0.983-0.993 | 0.813 | <.001 | 0.796-0.830 |
| European | 0.647 | <.001 | 0.627-0.667 | 0.932 | <.001 | 0.921-0.942 | 0.521 | <.001 | 0.500-0.543 | 0.958 | 0.0029 | 0.950-0.967 | 0.521 | <.001 | 0.500-0.543 |
| Hispanic | 0.869 | <.001 | 0.854-0.885 | 0.964 | 0.0537 | 0.956-0.972 | 0.721 | <.001 | 0.702-0.741 | 0.985 | <.001 | 0.980-0.991 | 0.721 | <.001 | 0.702-0.741 |
| Scandinavian | 0.468 | <.001 | 0.445-0.490 | 0.999 | <.001 | 0.995-1.002 | 0.98 | <.001 | 0.971-0.989 | 0.949 | <.001 | 0.940-0.959 | 0.98 | <.001 | 0.971-0.989 |
| South Asian | 0.904 | <.001 | 0.891-0.917 | 0.934 | <.001 | 0.924-0.944 | 0.617 | 0.0018 | 0.597-0.638 | 0.988 | <.001 | 0.983-0.993 | 0.617 | 0.0018 | 0.597-0.638 |

|  | F1 | | | Prevalence | | | Detection Rate | | | Detection Prevalence | | | Balanced Accuracy | | |
| --- | --- | --- | --- | --- | --- | --- | --- | --- | --- | --- | --- | --- | --- | --- | --- |
|  | Value | P | CI | Value | P | CI | Value | P | CI | Value | P | CI | Value | P | CI |
| African | 0.538 | <.001 | 0.518-0.558 | 0.113 | <.001 | 0.101-0.126 | 0.044 | <.001 | 0.036-0.053 | 0.052 | <.001 | 0.042-0.061 | 0.692 | <.001 | 0.674-0.710 |
| Anglo-American | 0.394 | <.001 | 0.371-0.416 | 0.082 | >.1 | 0.074-0.089 | 0.062 | >.1 | 0.060-0.065 | 0.236 | <.001 | 0.217-0.256 | 0.787 | <.001 | 0.768-0.806 |
| Arabic | 0.731 | <.001 | 0.712-0.750 | 0.1 | 0.0361 | 0.087-0.113 | 0.078 | <.001 | 0.067-0.089 | 0.113 | 0.0186 | 0.100-0.127 | 0.87 | <.001 | 0.855-0.884 |
| East Asian | 0.856 | <.001 | 0.841-0.872 | 0.104 | 0.0056 | 0.091-0.117 | 0.094 | <.001 | 0.082-0.106 | 0.115 | 0.006 | 0.102-0.129 | 0.94 | <.001 | 0.929-0.951 |
| European | 0.578 | >.1 | 0.571-0.584 | 0.102 | 0.0149 | 0.089-0.115 | 0.066 | >.1 | 0.059-0.072 | 0.127 | <.001 | 0.113-0.141 | 0.79 | <.001 | 0.772-0.807 |
| Hispanic | 0.789 | <.001 | 0.770-0.807 | 0.095 | >.1 | 0.088-0.103 | 0.083 | <.001 | 0.071-0.095 | 0.115 | 0.0097 | 0.101-0.129 | 0.917 | <.001 | 0.904-0.929 |
| Scandinavian | 0.633 | <.001 | 0.611-0.655 | 0.09 | >.1 | 0.088-0.091 | 0.042 | <.001 | 0.032-0.051 | 0.042 | <.001 | 0.033-0.052 | 0.733 | <.001 | 0.713-0.753 |
| South Asian | 0.734 | <.001 | 0.715-0.753 | 0.104 | 0.0042 | 0.091-0.117 | 0.094 | <.001 | 0.082-0.106 | 0.153 | <.001 | 0.138-0.168 | 0.919 | <.001 | 0.907-0.931 |

Over 55 Years

|  | F1 | | | Prevalence | | | Detection Rate | | | Detection Prevalence | | | Balanced Accuracy | | |
| --- | --- | --- | --- | --- | --- | --- | --- | --- | --- | --- | --- | --- | --- | --- | --- |
|  | Value | P | CI | Value | P | CI | Value | P | CI | Value | P | CI | Value | P | CI |
| African | 0.31 | <.001 | 0.273-0.348 | 0.992 | <.001 | 0.982-1.001 | 0.782 | <.001 | 0.748-0.816 | 0.939 | <.001 | 0.920-0.959 | 0.782 | <.001 | 0.748-0.816 |
| Anglo-American | 0.877 | <.001 | 0.857-0.898 | 0.789 | <.001 | 0.768-0.810 | 0.508 | <.001 | 0.480-0.536 | 0.963 | >.1 | 0.962-0.963 | 0.508 | <.001 | 0.480-0.536 |
| Arabic | 0.699 | >.1 | 0.699-0.699 | 0.966 | >.1 | 0.953-0.978 | 0.497 | <.001 | 0.442-0.551 | 0.985 | 0.0274 | 0.970-1.000 | 0.497 | <.001 | 0.442-0.551 |
| East Asian | 0.83 | <.001 | 0.798-0.862 | 0.977 | 0.0036 | 0.964-0.991 | 0.771 | <.001 | 0.736-0.806 | 0.984 | 0.0035 | 0.973-0.995 | 0.771 | <.001 | 0.736-0.806 |
| European | 0.656 | 0.0151 | 0.617-0.696 | 0.904 | <.001 | 0.880-0.929 | 0.377 | <.001 | 0.337-0.418 | 0.967 | >.1 | 0.962-0.972 | 0.377 | <.001 | 0.337-0.418 |
| Hispanic | 0.86 | <.001 | 0.827-0.893 | 0.958 | >.1 | 0.954-0.962 | 0.59 | >.1 | 0.571-0.609 | 0.989 | 0.0011 | 0.979-1.000 | 0.59 | >.1 | 0.571-0.609 |
| Scandinavian | 0.592 | <.001 | 0.562-0.622 | 0.998 | <.001 | 0.992-1.004 | 0.989 | <.001 | 0.975-1.003 | 0.927 | <.001 | 0.912-0.943 | 0.989 | <.001 | 0.975-1.003 |
| South Asian | 0.816 | <.001 | 0.775-0.857 | 0.963 | >.1 | 0.953-0.973 | 0.543 | 0.005 | 0.491-0.594 | 0.989 | 0.0044 | 0.977-1.002 | 0.543 | 0.005 | 0.491-0.594 |

|  | Recall | | | F1 | | | Prevalence | | | Detection Rate | | | Detection Prevalence | | |
| --- | --- | --- | --- | --- | --- | --- | --- | --- | --- | --- | --- | --- | --- | --- | --- |
|  | Value | P | CI | Value | P | CI | Value | P | CI | Value | P | CI | Value | P | CI |
| African | 0.444 | <.001 | 0.404-0.485 | 0.084 | >.1 | 0.075-0.094 | 0.026 | <.001 | 0.012-0.040 | 0.033 | <.001 | 0.017-0.049 | 0.651 | <.001 | 0.613-0.689 |
| Anglo-American | 0.644 | <.001 | 0.616-0.671 | 0.198 | <.001 | 0.177-0.220 | 0.174 | <.001 | 0.154-0.194 | 0.343 | <.001 | 0.318-0.368 | 0.833 | >.1 | 0.826-0.841 |
| Arabic | 0.581 | >.1 | 0.558-0.604 | 0.045 | 0.0013 | 0.021-0.069 | 0.031 | 0.0215 | 0.011-0.052 | 0.064 | 0.0203 | 0.036-0.092 | 0.832 | >.1 | 0.826-0.839 |
| East Asian | 0.8 | <.001 | 0.766-0.833 | 0.082 | >.1 | 0.070-0.094 | 0.068 | >.1 | 0.059-0.077 | 0.088 | >.1 | 0.076-0.101 | 0.904 | <.001 | 0.878-0.929 |
| European | 0.479 | <.001 | 0.438-0.521 | 0.08 | >.1 | 0.066-0.094 | 0.053 | >.1 | 0.044-0.061 | 0.14 | <.001 | 0.111-0.169 | 0.78 | 0.0014 | 0.746-0.815 |
| Hispanic | 0.7 | <.001 | 0.657-0.742 | 0.065 | 0.0248 | 0.041-0.088 | 0.056 | >.1 | 0.051-0.060 | 0.095 | >.1 | 0.089-0.100 | 0.909 | <.001 | 0.881-0.936 |
| Scandinavian | 0.741 | <.001 | 0.713-0.768 | 0.16 | <.001 | 0.138-0.182 | 0.095 | <.001 | 0.077-0.112 | 0.096 | >.1 | 0.091-0.100 | 0.795 | 0.0035 | 0.770-0.820 |
| South Asian | 0.652 | <.001 | 0.602-0.702 | 0.05 | 0.0024 | 0.026-0.074 | 0.041 | >.1 | 0.019-0.062 | 0.075 | >.1 | 0.049-0.102 | 0.89 | <.001 | 0.856-0.923 |

Ethnicolr

All

|  | Sensitivity | | | Specificity | | | Positive Predictive Value | | | Negative Predictive Value | | | Precision | | |
| --- | --- | --- | --- | --- | --- | --- | --- | --- | --- | --- | --- | --- | --- | --- | --- |
|  | Value | P | CI | Value | P | CI | Value | P | CI | Value | P | CI | Value | P | CI |
| African | 0.333 | <.001 | 0.318-0.348 | 0.954 | >.1 | 0.949-0.959 | 0.344 | <.001 | 0.328-0.359 | 0.952 | 0.0057 | 0.945-0.959 | 0.344 | <.001 | 0.328-0.359 |
| Arabic | 0.526 | <.001 | 0.509-0.542 | 0.918 | <.001 | 0.910-0.927 | 0.316 | <.001 | 0.301-0.331 | 0.964 | >.1 | 0.960-0.968 | 0.316 | <.001 | 0.301-0.331 |
| British | 0.643 | <.001 | 0.628-0.659 | 0.854 | <.001 | 0.843-0.865 | 0.239 | <.001 | 0.225-0.253 | 0.971 | <.001 | 0.965-0.976 | 0.239 | <.001 | 0.225-0.253 |
| East Asian | 0.439 | >.1 | 0.426-0.453 | 0.992 | <.001 | 0.988-0.995 | 0.798 | <.001 | 0.785-0.811 | 0.961 | >.1 | 0.960-0.961 | 0.798 | <.001 | 0.785-0.811 |
| East European | 0.365 | <.001 | 0.350-0.381 | 0.94 | 0.0056 | 0.932-0.948 | 0.305 | <.001 | 0.290-0.320 | 0.954 | 0.026 | 0.947-0.960 | 0.305 | <.001 | 0.290-0.320 |
| French | 0.269 | <.001 | 0.255-0.284 | 0.974 | <.001 | 0.969-0.979 | 0.431 | >.1 | 0.421-0.440 | 0.949 | <.001 | 0.942-0.956 | 0.431 | >.1 | 0.421-0.440 |
| Germanic | 0.178 | <.001 | 0.165-0.190 | 0.984 | <.001 | 0.980-0.988 | 0.45 | >.1 | 0.438-0.463 | 0.943 | <.001 | 0.936-0.951 | 0.45 | >.1 | 0.438-0.463 |
| Hispanic | 0.5 | <.001 | 0.484-0.516 | 0.952 | >.1 | 0.949-0.954 | 0.427 | >.1 | 0.414-0.440 | 0.963 | >.1 | 0.960-0.967 | 0.427 | >.1 | 0.414-0.440 |
| Indian | 0.501 | <.001 | 0.484-0.517 | 0.93 | <.001 | 0.922-0.939 | 0.341 | <.001 | 0.326-0.356 | 0.963 | >.1 | 0.960-0.965 | 0.341 | <.001 | 0.326-0.356 |
| Italian | 0.696 | <.001 | 0.681-0.711 | 0.929 | <.001 | 0.921-0.938 | 0.415 | 0.0012 | 0.399-0.431 | 0.977 | <.001 | 0.972-0.982 | 0.415 | 0.0012 | 0.399-0.431 |
| Japanese | 0.854 | <.001 | 0.842-0.866 | 0.992 | <.001 | 0.988-0.995 | 0.887 | <.001 | 0.876-0.898 | 0.989 | <.001 | 0.985-0.993 | 0.887 | <.001 | 0.876-0.898 |
| Scandinavian | 0.196 | <.001 | 0.183-0.209 | 0.982 | <.001 | 0.978-0.987 | 0.45 | >.1 | 0.438-0.462 | 0.944 | <.001 | 0.937-0.952 | 0.45 | >.1 | 0.438-0.462 |

|  | F1 | | | Prevalence | | | Detection Rate | | | Detection Prevalence | | | Balanced Accuracy | | |
| --- | --- | --- | --- | --- | --- | --- | --- | --- | --- | --- | --- | --- | --- | --- | --- |
|  | Value | P | CI | Value | P | CI | Value | P | CI | Value | P | CI | Value | P | CI |
| African | 0.338 | <.001 | 0.323-0.354 | 0.066 | >.1 | 0.066-0.066 | 0.022 | 0.0026 | 0.017-0.027 | 0.064 | 0.0025 | 0.056-0.072 | 0.644 | <.001 | 0.628-0.659 |
| Arabic | 0.395 | 0.0019 | 0.379-0.410 | 0.066 | >.1 | 0.066-0.066 | 0.035 | 0.0624 | 0.029-0.040 | 0.11 | <.001 | 0.100-0.120 | 0.722 | 0.002 | 0.707-0.736 |
| British | 0.349 | <.001 | 0.333-0.364 | 0.066 | >.1 | 0.066-0.066 | 0.042 | <.001 | 0.036-0.049 | 0.178 | <.001 | 0.166-0.191 | 0.748 | <.001 | 0.734-0.763 |
| East Asian | 0.567 | <.001 | 0.551-0.583 | 0.066 | >.1 | 0.066-0.066 | 0.029 | >.1 | 0.028-0.030 | 0.036 | <.001 | 0.030-0.043 | 0.715 | 0.032 | 0.701-0.730 |
| East European | 0.332 | <.001 | 0.317-0.348 | 0.066 | >.1 | 0.066-0.066 | 0.024 | 0.0295 | 0.019-0.029 | 0.079 | >.1 | 0.076-0.082 | 0.653 | <.001 | 0.637-0.668 |
| French | 0.331 | <.001 | 0.316-0.347 | 0.066 | >.1 | 0.066-0.066 | 0.017 | <.001 | 0.013-0.022 | 0.041 | <.001 | 0.034-0.048 | 0.622 | <.001 | 0.606-0.637 |
| Germanic | 0.255 | <.001 | 0.241-0.269 | 0.066 | >.1 | 0.066-0.066 | 0.011 | <.001 | 0.008-0.015 | 0.026 | <.001 | 0.020-0.031 | 0.581 | <.001 | 0.565-0.597 |
| Hispanic | 0.461 | <.001 | 0.445-0.477 | 0.066 | >.1 | 0.066-0.066 | 0.033 | >.1 | 0.029-0.036 | 0.078 | >.1 | 0.076-0.079 | 0.726 | <.001 | 0.711-0.740 |
| Indian | 0.406 | >.1 | 0.392-0.419 | 0.066 | >.1 | 0.066-0.066 | 0.033 | >.1 | 0.029-0.036 | 0.097 | <.001 | 0.088-0.107 | 0.715 | 0.031 | 0.701-0.730 |
| Italian | 0.52 | <.001 | 0.504-0.536 | 0.066 | >.1 | 0.066-0.066 | 0.046 | <.001 | 0.039-0.053 | 0.111 | <.001 | 0.101-0.121 | 0.813 | <.001 | 0.800-0.826 |
| Japanese | 0.87 | <.001 | 0.859-0.882 | 0.066 | >.1 | 0.066-0.066 | 0.056 | <.001 | 0.049-0.064 | 0.064 | 0.0018 | 0.056-0.072 | 0.923 | <.001 | 0.914-0.932 |
| Scandinavian | 0.273 | <.001 | 0.258-0.288 | 0.066 | >.1 | 0.066-0.066 | 0.013 | <.001 | 0.009-0.017 | 0.029 | <.001 | 0.023-0.034 | 0.589 | <.001 | 0.573-0.605 |

Female

|  | Sensitivity | | | Specificity | | | Positive Predictive Value | | | Negative Predictive Value | | | Precision | | |
| --- | --- | --- | --- | --- | --- | --- | --- | --- | --- | --- | --- | --- | --- | --- | --- |
|  | Value | P | CI | Value | P | CI | Value | P | CI | Value | P | CI | Value | P | CI |
| African | 0.355 | 0.0012 | 0.327-0.383 | 0.919 | <.001 | 0.903-0.935 | 0.264 | <.001 | 0.238-0.290 | 0.946 | 0.0301 | 0.932-0.959 | 0.264 | <.001 | 0.238-0.290 |
| Arabic | 0.371 | >.1 | 0.342-0.400 | 0.956 | >.1 | 0.947-0.965 | 0.269 | <.001 | 0.235-0.304 | 0.972 | 0.0583 | 0.959-0.985 | 0.269 | <.001 | 0.235-0.304 |
| British | 0.479 | <.001 | 0.447-0.510 | 0.875 | <.001 | 0.855-0.895 | 0.207 | <.001 | 0.181-0.233 | 0.961 | >.1 | 0.957-0.964 | 0.207 | <.001 | 0.181-0.233 |
| East Asian | 0.396 | >.1 | 0.395-0.398 | 0.99 | <.001 | 0.984-0.996 | 0.83 | <.001 | 0.811-0.849 | 0.931 | <.001 | 0.918-0.943 | 0.83 | <.001 | 0.811-0.849 |
| East European | 0.396 | >.1 | 0.393-0.398 | 0.936 | >.1 | 0.923-0.949 | 0.254 | <.001 | 0.223-0.284 | 0.965 | >.1 | 0.957-0.974 | 0.254 | <.001 | 0.223-0.284 |
| French | 0.199 | <.001 | 0.174-0.225 | 0.97 | <.001 | 0.959-0.981 | 0.322 | <.001 | 0.293-0.351 | 0.945 | 0.0332 | 0.931-0.959 | 0.322 | <.001 | 0.293-0.351 |
| Germanic | 0.086 | <.001 | 0.067-0.105 | 0.992 | <.001 | 0.985-0.999 | 0.432 | 0.0027 | 0.401-0.464 | 0.943 | 0.0139 | 0.928-0.958 | 0.432 | 0.0027 | 0.401-0.464 |
| Hispanic | 0.485 | <.001 | 0.455-0.515 | 0.938 | >.1 | 0.927-0.948 | 0.369 | >.1 | 0.348-0.390 | 0.96 | >.1 | 0.957-0.963 | 0.369 | >.1 | 0.348-0.390 |
| Indian | 0.604 | <.001 | 0.572-0.637 | 0.927 | 0.0027 | 0.909-0.944 | 0.331 | <.001 | 0.299-0.362 | 0.975 | 0.0073 | 0.964-0.986 | 0.331 | <.001 | 0.299-0.362 |
| Italian | 0.671 | <.001 | 0.639-0.704 | 0.883 | <.001 | 0.861-0.904 | 0.241 | <.001 | 0.212-0.271 | 0.979 | <.001 | 0.969-0.990 | 0.241 | <.001 | 0.212-0.271 |
| Japanese | 0.748 | <.001 | 0.727-0.768 | 0.988 | <.001 | 0.982-0.994 | 0.899 | <.001 | 0.884-0.915 | 0.966 | 0.0585 | 0.957-0.975 | 0.899 | <.001 | 0.884-0.915 |
| Scandinavian | 0.137 | <.001 | 0.115-0.159 | 0.986 | <.001 | 0.978-0.994 | 0.426 | 0.0071 | 0.396-0.456 | 0.94 | 0.0025 | 0.926-0.955 | 0.426 | 0.0071 | 0.396-0.456 |

|  | F1 | | | Prevalence | | | Detection Rate | | | Detection Prevalence | | | Balanced Accuracy | | |
| --- | --- | --- | --- | --- | --- | --- | --- | --- | --- | --- | --- | --- | --- | --- | --- |
|  | Value | P | CI | Value | P | CI | Value | P | CI | Value | P | CI | Value | P | CI |
| African | 0.303 | <.001 | 0.276-0.329 | 0.075 | >.1 | 0.066-0.083 | 0.026 | >.1 | 0.025-0.028 | 0.101 | <.001 | 0.083-0.118 | 0.637 | 0.0051 | 0.609-0.665 |
| Arabic | 0.312 | 0.0181 | 0.276-0.348 | 0.041 | 0.0052 | 0.025-0.057 | 0.015 | 0.0417 | 0.005-0.025 | 0.057 | 0.0446 | 0.038-0.075 | 0.664 | >.1 | 0.654-0.673 |
| British | 0.289 | <.001 | 0.260-0.317 | 0.063 | >.1 | 0.059-0.067 | 0.03 | >.1 | 0.027-0.032 | 0.147 | <.001 | 0.125-0.169 | 0.677 | >.1 | 0.672-0.681 |
| East Asian | 0.537 | <.001 | 0.512-0.561 | 0.108 | <.001 | 0.093-0.123 | 0.042 | <.001 | 0.032-0.052 | 0.051 | <.001 | 0.040-0.062 | 0.693 | 0.0606 | 0.671-0.715 |
| East European | 0.309 | 0.0046 | 0.277-0.341 | 0.051 | 0.0651 | 0.036-0.067 | 0.02 | >.1 | 0.012-0.028 | 0.08 | >.1 | 0.076-0.085 | 0.666 | >.1 | 0.658-0.673 |
| French | 0.246 | <.001 | 0.219-0.273 | 0.065 | >.1 | 0.063-0.067 | 0.013 | 0.0021 | 0.005-0.020 | 0.04 | <.001 | 0.027-0.053 | 0.585 | <.001 | 0.554-0.615 |
| Germanic | 0.143 | <.001 | 0.120-0.166 | 0.061 | >.1 | 0.055-0.067 | 0.005 | <.001 | -0.01 | 0.012 | <.001 | 0.003-0.020 | 0.539 | <.001 | 0.507-0.570 |
| Hispanic | 0.419 | <.001 | 0.389-0.449 | 0.069 | >.1 | 0.067-0.071 | 0.033 | >.1 | 0.027-0.039 | 0.091 | 0.0635 | 0.075-0.106 | 0.711 | 0.0035 | 0.684-0.739 |
| Indian | 0.427 | <.001 | 0.395-0.460 | 0.056 | >.1 | 0.044-0.068 | 0.034 | >.1 | 0.027-0.040 | 0.102 | 0.002 | 0.082-0.122 | 0.765 | <.001 | 0.737-0.794 |
| Italian | 0.355 | >.1 | 0.353-0.357 | 0.052 | 0.0769 | 0.036-0.068 | 0.035 | >.1 | 0.027-0.043 | 0.146 | <.001 | 0.121-0.170 | 0.777 | <.001 | 0.748-0.806 |
| Japanese | 0.817 | <.001 | 0.798-0.835 | 0.119 | <.001 | 0.104-0.134 | 0.089 | <.001 | 0.076-0.102 | 0.099 | <.001 | 0.085-0.113 | 0.868 | <.001 | 0.851-0.885 |
| Scandinavian | 0.207 | <.001 | 0.182-0.232 | 0.067 | >.1 | 0.067-0.067 | 0.009 | <.001 | 0.002-0.015 | 0.021 | <.001 | 0.011-0.031 | 0.561 | <.001 | 0.531-0.592 |

Male

|  | Sensitivity | | | Specificity | | | Positive Predictive Value | | | Negative Predictive Value | | | Precision | | |
| --- | --- | --- | --- | --- | --- | --- | --- | --- | --- | --- | --- | --- | --- | --- | --- |
|  | Value | P | CI | Value | P | CI | Value | P | CI | Value | P | CI | Value | P | CI |
| African | 0.323 | <.001 | 0.304-0.341 | 0.968 | <.001 | 0.961-0.975 | 0.407 | <.001 | 0.387-0.426 | 0.954 | 0.0605 | 0.947-0.962 | 0.407 | <.001 | 0.387-0.426 |
| Arabic | 0.559 | <.001 | 0.541-0.576 | 0.903 | <.001 | 0.892-0.913 | 0.324 | <.001 | 0.307-0.341 | 0.961 | >.1 | 0.960-0.961 | 0.324 | <.001 | 0.307-0.341 |
| British | 0.704 | <.001 | 0.687-0.722 | 0.845 | <.001 | 0.832-0.859 | 0.249 | <.001 | 0.233-0.266 | 0.975 | <.001 | 0.969-0.981 | 0.249 | <.001 | 0.233-0.266 |
| East Asian | 0.476 | >.1 | 0.476-0.476 | 0.992 | <.001 | 0.988-0.997 | 0.777 | <.001 | 0.758-0.795 | 0.972 | 0.0054 | 0.965-0.980 | 0.777 | <.001 | 0.758-0.795 |
| East European | 0.357 | <.001 | 0.339-0.374 | 0.942 | 0.0309 | 0.933-0.950 | 0.326 | <.001 | 0.308-0.343 | 0.949 | <.001 | 0.941-0.957 | 0.326 | <.001 | 0.308-0.343 |
| French | 0.296 | <.001 | 0.279-0.314 | 0.976 | <.001 | 0.969-0.982 | 0.472 | >.1 | 0.460-0.484 | 0.95 | 0.0015 | 0.942-0.958 | 0.472 | >.1 | 0.460-0.484 |
| Germanic | 0.21 | <.001 | 0.195-0.226 | 0.981 | <.001 | 0.975-0.986 | 0.453 | >.1 | 0.445-0.461 | 0.943 | <.001 | 0.935-0.952 | 0.453 | >.1 | 0.445-0.461 |
| Hispanic | 0.507 | <.001 | 0.487-0.526 | 0.957 | 0.0776 | 0.949-0.965 | 0.456 | >.1 | 0.451-0.461 | 0.965 | >.1 | 0.961-0.968 | 0.456 | >.1 | 0.451-0.461 |
| Indian | 0.468 | >.1 | 0.460-0.476 | 0.932 | <.001 | 0.923-0.941 | 0.345 | <.001 | 0.328-0.363 | 0.958 | >.1 | 0.954-0.961 | 0.345 | <.001 | 0.328-0.363 |
| Italian | 0.703 | <.001 | 0.686-0.720 | 0.948 | >.1 | 0.947-0.950 | 0.517 | <.001 | 0.498-0.535 | 0.976 | <.001 | 0.970-0.982 | 0.517 | <.001 | 0.498-0.535 |
| Japanese | 0.964 | <.001 | 0.954-0.974 | 0.993 | <.001 | 0.989-0.997 | 0.877 | <.001 | 0.862-0.893 | 0.998 | <.001 | 0.995-1.001 | 0.877 | <.001 | 0.862-0.893 |
| Scandinavian | 0.219 | <.001 | 0.203-0.236 | 0.981 | <.001 | 0.975-0.987 | 0.457 | >.1 | 0.453-0.461 | 0.946 | <.001 | 0.937-0.955 | 0.457 | >.1 | 0.453-0.461 |

|  | F1 | | | Prevalence | | | Detection Rate | | | Detection Prevalence | | | Balanced Accuracy | | |
| --- | --- | --- | --- | --- | --- | --- | --- | --- | --- | --- | --- | --- | --- | --- | --- |
|  | Value | P | CI | Value | P | CI | Value | P | CI | Value | P | CI | Value | P | CI |
| African | 0.36 | <.001 | 0.341-0.379 | 0.063 | >.1 | 0.059-0.066 | 0.02 | 0.0012 | 0.014-0.026 | 0.05 | <.001 | 0.041-0.059 | 0.645 | <.001 | 0.627-0.664 |
| Arabic | 0.41 | <.001 | 0.392-0.428 | 0.076 | 0.0164 | 0.067-0.086 | 0.042 | <.001 | 0.035-0.050 | 0.132 | <.001 | 0.120-0.144 | 0.731 | 0.0188 | 0.715-0.747 |
| British | 0.368 | <.001 | 0.350-0.387 | 0.067 | >.1 | 0.066-0.069 | 0.047 | <.001 | 0.039-0.055 | 0.191 | <.001 | 0.176-0.206 | 0.775 | <.001 | 0.759-0.791 |
| East Asian | 0.59 | <.001 | 0.569-0.612 | 0.05 | 0.0017 | 0.040-0.060 | 0.023 | 0.0567 | 0.017-0.030 | 0.03 | <.001 | 0.022-0.038 | 0.734 | 0.0228 | 0.715-0.753 |
| East European | 0.341 | <.001 | 0.323-0.358 | 0.072 | >.1 | 0.065-0.079 | 0.025 | >.1 | 0.020-0.031 | 0.079 | >.1 | 0.076-0.082 | 0.649 | <.001 | 0.632-0.667 |
| French | 0.364 | <.001 | 0.346-0.382 | 0.067 | >.1 | 0.066-0.067 | 0.019 | <.001 | 0.014-0.025 | 0.042 | <.001 | 0.034-0.050 | 0.636 | <.001 | 0.618-0.654 |
| Germanic | 0.287 | <.001 | 0.270-0.305 | 0.068 | >.1 | 0.066-0.071 | 0.014 | <.001 | 0.009-0.019 | 0.031 | <.001 | 0.024-0.038 | 0.596 | <.001 | 0.577-0.614 |
| Hispanic | 0.48 | <.001 | 0.461-0.499 | 0.065 | >.1 | 0.064-0.066 | 0.033 | >.1 | 0.030-0.035 | 0.072 | >.1 | 0.068-0.077 | 0.732 | 0.0197 | 0.715-0.749 |
| Indian | 0.397 | <.001 | 0.379-0.416 | 0.07 | >.1 | 0.066-0.075 | 0.033 | >.1 | 0.030-0.035 | 0.095 | <.001 | 0.084-0.106 | 0.7 | >.1 | 0.686-0.714 |
| Italian | 0.596 | <.001 | 0.578-0.614 | 0.072 | >.1 | 0.065-0.078 | 0.05 | <.001 | 0.042-0.058 | 0.098 | <.001 | 0.087-0.109 | 0.826 | <.001 | 0.812-0.840 |
| Japanese | 0.918 | <.001 | 0.905-0.932 | 0.045 | <.001 | 0.035-0.055 | 0.044 | <.001 | 0.034-0.053 | 0.05 | <.001 | 0.040-0.060 | 0.979 | <.001 | 0.970-0.987 |
| Scandinavian | 0.296 | <.001 | 0.279-0.314 | 0.066 | >.1 | 0.066-0.066 | 0.014 | <.001 | 0.009-0.019 | 0.031 | <.001 | 0.024-0.039 | 0.6 | <.001 | 0.581-0.619 |

Under 35 Years

|  | Sensitivity | | | Specificity | | | Positive Predictive Value | | | Negative Predictive Value | | | Precision | | |
| --- | --- | --- | --- | --- | --- | --- | --- | --- | --- | --- | --- | --- | --- | --- | --- |
|  | Value | P | CI | Value | P | CI | Value | P | CI | Value | P | CI | Value | P | CI |
| African | 0.34 | <.001 | 0.301-0.378 | 0.951 | >.1 | 0.944-0.957 | 0.283 | <.001 | 0.246-0.320 | 0.962 | >.1 | 0.957-0.966 | 0.283 | <.001 | 0.246-0.320 |
| Arabic | 0.535 | <.001 | 0.505-0.565 | 0.878 | <.001 | 0.858-0.897 | 0.332 | 0.0016 | 0.303-0.360 | 0.943 | 0.0153 | 0.929-0.957 | 0.332 | 0.0016 | 0.303-0.360 |
| British | 0.592 | <.001 | 0.546-0.638 | 0.858 | <.001 | 0.826-0.890 | 0.152 | <.001 | 0.118-0.186 | 0.98 | 0.014 | 0.965-0.994 | 0.152 | <.001 | 0.118-0.186 |
| East Asian | 0.357 | <.001 | 0.328-0.387 | 0.991 | <.001 | 0.984-0.998 | 0.822 | <.001 | 0.798-0.846 | 0.933 | <.001 | 0.917-0.948 | 0.822 | <.001 | 0.798-0.846 |
| East European | 0.31 | <.001 | 0.281-0.339 | 0.938 | >.1 | 0.931-0.945 | 0.346 | 0.0427 | 0.316-0.376 | 0.928 | <.001 | 0.912-0.944 | 0.346 | 0.0427 | 0.316-0.376 |
| French | 0.227 | <.001 | 0.197-0.258 | 0.974 | <.001 | 0.962-0.987 | 0.406 | 0.0629 | 0.372-0.439 | 0.943 | 0.0485 | 0.928-0.958 | 0.406 | 0.0629 | 0.372-0.439 |
| Germanic | 0.104 | <.001 | 0.075-0.133 | 0.989 | <.001 | 0.978-1.000 | 0.31 | 0.0014 | 0.269-0.352 | 0.959 | >.1 | 0.957-0.961 | 0.31 | 0.0014 | 0.269-0.352 |
| Hispanic | 0.481 | <.001 | 0.445-0.516 | 0.948 | >.1 | 0.944-0.952 | 0.421 | 0.004 | 0.386-0.456 | 0.959 | >.1 | 0.957-0.960 | 0.421 | 0.004 | 0.386-0.456 |
| Indian | 0.463 | <.001 | 0.433-0.493 | 0.918 | <.001 | 0.902-0.935 | 0.391 | >.1 | 0.374-0.408 | 0.938 | <.001 | 0.923-0.952 | 0.391 | >.1 | 0.374-0.408 |
| Italian | 0.615 | <.001 | 0.576-0.654 | 0.92 | 0.0044 | 0.898-0.942 | 0.314 | <.001 | 0.277-0.351 | 0.975 | 0.0203 | 0.962-0.988 | 0.314 | <.001 | 0.277-0.351 |
| Japanese | 0.847 | <.001 | 0.792-0.901 | 0.991 | 0.0053 | 0.974-1.007 | 0.616 | <.001 | 0.544-0.689 | 0.997 | 0.0076 | 0.986-1.008 | 0.616 | <.001 | 0.544-0.689 |
| Scandinavian | 0.118 | <.001 | 0.090-0.146 | 0.983 | <.001 | 0.972-0.995 | 0.289 | <.001 | 0.251-0.326 | 0.952 | >.1 | 0.946-0.958 | 0.289 | <.001 | 0.251-0.326 |

|  | F1 | | | Prevalence | | | Detection Rate | | | Detection Prevalence | | | Balanced Accuracy | | |
| --- | --- | --- | --- | --- | --- | --- | --- | --- | --- | --- | --- | --- | --- | --- | --- |
|  | Value | P | CI | Value | P | CI | Value | P | CI | Value | P | CI | Value | P | CI |
| African | 0.309 | 0.0066 | 0.271-0.347 | 0.053 | >.1 | 0.041-0.065 | 0.018 | >.1 | 0.010-0.026 | 0.064 | >.1 | 0.051-0.077 | 0.645 | 0.0577 | 0.608-0.682 |
| Arabic | 0.41 | <.001 | 0.380-0.439 | 0.101 | <.001 | 0.083-0.119 | 0.054 | <.001 | 0.040-0.068 | 0.164 | <.001 | 0.142-0.186 | 0.706 | 0.0414 | 0.679-0.734 |
| British | 0.242 | <.001 | 0.202-0.282 | 0.041 | 0.0335 | 0.021-0.060 | 0.024 | >.1 | 0.023-0.025 | 0.159 | <.001 | 0.125-0.193 | 0.725 | 0.0247 | 0.683-0.767 |
| East Asian | 0.498 | <.001 | 0.468-0.529 | 0.099 | <.001 | 0.081-0.117 | 0.035 | 0.0337 | 0.024-0.046 | 0.043 | <.001 | 0.030-0.056 | 0.674 | >.1 | 0.669-0.680 |
| East European | 0.327 | 0.0258 | 0.298-0.357 | 0.095 | <.001 | 0.076-0.113 | 0.029 | >.1 | 0.025-0.033 | 0.085 | >.1 | 0.076-0.094 | 0.624 | <.001 | 0.594-0.654 |
| French | 0.291 | <.001 | 0.258-0.324 | 0.069 | >.1 | 0.064-0.075 | 0.015 | 0.0838 | 0.006-0.025 | 0.039 | <.001 | 0.024-0.054 | 0.601 | <.001 | 0.566-0.636 |
| Germanic | 0.156 | <.001 | 0.123-0.189 | 0.044 | 0.0594 | 0.025-0.063 | 0.004 | 0.0024 | -0.012 | 0.014 | <.001 | 0.002-0.027 | 0.546 | <.001 | 0.502-0.591 |
| Hispanic | 0.449 | <.001 | 0.414-0.484 | 0.072 | >.1 | 0.063-0.080 | 0.034 | 0.0942 | 0.024-0.044 | 0.082 | >.1 | 0.076-0.088 | 0.714 | 0.0238 | 0.682-0.746 |
| Indian | 0.424 | <.001 | 0.394-0.454 | 0.101 | <.001 | 0.083-0.119 | 0.046 | <.001 | 0.034-0.059 | 0.119 | <.001 | 0.100-0.139 | 0.691 | >.1 | 0.678-0.703 |
| Italian | 0.416 | 0.0014 | 0.376-0.455 | 0.056 | >.1 | 0.046-0.065 | 0.034 | >.1 | 0.024-0.044 | 0.109 | 0.001 | 0.084-0.134 | 0.767 | <.001 | 0.733-0.801 |
| Japanese | 0.713 | <.001 | 0.646-0.781 | 0.016 | 0.0066 | -0.037 | 0.013 | >.1 | 0.000-0.026 | 0.022 | 0.0044 | -0.046 | 0.919 | <.001 | 0.877-0.961 |
| Scandinavian | 0.167 | <.001 | 0.136-0.199 | 0.052 | >.1 | 0.040-0.065 | 0.006 | 0.0021 | -0.013 | 0.021 | <.001 | 0.008-0.034 | 0.551 | <.001 | 0.510-0.591 |

35-55 Years

|  | Sensitivity | | | Specificity | | | Positive Predictive Value | | | Negative Predictive Value | | | Precision | | |
| --- | --- | --- | --- | --- | --- | --- | --- | --- | --- | --- | --- | --- | --- | --- | --- |
|  | Value | P | CI | Value | P | CI | Value | P | CI | Value | P | CI | Value | P | CI |
| African | 0.342 | <.001 | 0.324-0.361 | 0.952 | >.1 | 0.949-0.955 | 0.38 | <.001 | 0.360-0.399 | 0.944 | <.001 | 0.935-0.953 | 0.38 | <.001 | 0.360-0.399 |
| Arabic | 0.508 | <.001 | 0.487-0.530 | 0.918 | <.001 | 0.907-0.930 | 0.311 | <.001 | 0.292-0.331 | 0.962 | >.1 | 0.960-0.965 | 0.311 | <.001 | 0.292-0.331 |
| British | 0.612 | <.001 | 0.590-0.634 | 0.858 | <.001 | 0.842-0.874 | 0.21 | <.001 | 0.192-0.229 | 0.972 | 0.0036 | 0.965-0.980 | 0.21 | <.001 | 0.192-0.229 |
| East Asian | 0.478 | 0.0034 | 0.457-0.500 | 0.991 | <.001 | 0.986-0.996 | 0.798 | <.001 | 0.780-0.815 | 0.964 | >.1 | 0.960-0.968 | 0.798 | <.001 | 0.780-0.815 |
| East European | 0.38 | <.001 | 0.360-0.400 | 0.938 | 0.0111 | 0.929-0.948 | 0.335 | <.001 | 0.316-0.354 | 0.949 | 0.002 | 0.940-0.958 | 0.335 | <.001 | 0.316-0.354 |
| French | 0.269 | <.001 | 0.249-0.288 | 0.974 | <.001 | 0.967-0.982 | 0.424 | >.1 | 0.410-0.438 | 0.95 | 0.0157 | 0.941-0.960 | 0.424 | >.1 | 0.410-0.438 |
| Germanic | 0.172 | <.001 | 0.154-0.189 | 0.985 | <.001 | 0.979-0.991 | 0.433 | >.1 | 0.430-0.437 | 0.949 | 0.0077 | 0.939-0.959 | 0.433 | >.1 | 0.430-0.437 |
| Hispanic | 0.504 | <.001 | 0.483-0.524 | 0.95 | >.1 | 0.949-0.952 | 0.444 | >.1 | 0.436-0.452 | 0.96 | >.1 | 0.960-0.961 | 0.444 | >.1 | 0.436-0.452 |
| Indian | 0.505 | <.001 | 0.484-0.526 | 0.925 | <.001 | 0.914-0.936 | 0.339 | <.001 | 0.319-0.358 | 0.961 | >.1 | 0.960-0.961 | 0.339 | <.001 | 0.319-0.358 |
| Italian | 0.686 | <.001 | 0.666-0.706 | 0.93 | <.001 | 0.919-0.941 | 0.404 | 0.0013 | 0.383-0.426 | 0.977 | <.001 | 0.970-0.984 | 0.404 | 0.0013 | 0.383-0.426 |
| Japanese | 0.825 | <.001 | 0.808-0.842 | 0.992 | <.001 | 0.987-0.996 | 0.873 | <.001 | 0.858-0.888 | 0.988 | <.001 | 0.983-0.993 | 0.873 | <.001 | 0.858-0.888 |
| Scandinavian | 0.187 | <.001 | 0.169-0.205 | 0.984 | <.001 | 0.978-0.990 | 0.432 | >.1 | 0.426-0.437 | 0.949 | 0.0081 | 0.939-0.959 | 0.432 | >.1 | 0.426-0.437 |

|  | F1 | | | Prevalence | | | Detection Rate | | | Detection Prevalence | | | Balanced Accuracy | | |
| --- | --- | --- | --- | --- | --- | --- | --- | --- | --- | --- | --- | --- | --- | --- | --- |
|  | Value | P | CI | Value | P | CI | Value | P | CI | Value | P | CI | Value | P | CI |
| African | 0.36 | <.001 | 0.341-0.379 | 0.078 | 0.0109 | 0.067-0.088 | 0.026 | >.1 | 0.023-0.030 | 0.07 | >.1 | 0.063-0.077 | 0.647 | <.001 | 0.628-0.666 |
| Arabic | 0.386 | 0.0021 | 0.366-0.407 | 0.067 | >.1 | 0.066-0.068 | 0.034 | >.1 | 0.029-0.039 | 0.109 | <.001 | 0.096-0.123 | 0.713 | >.1 | 0.698-0.729 |
| British | 0.313 | <.001 | 0.292-0.334 | 0.058 | >.1 | 0.049-0.067 | 0.035 | >.1 | 0.029-0.041 | 0.168 | <.001 | 0.152-0.185 | 0.735 | <.001 | 0.715-0.755 |
| East Asian | 0.598 | <.001 | 0.577-0.619 | 0.065 | >.1 | 0.064-0.066 | 0.031 | >.1 | 0.029-0.032 | 0.039 | <.001 | 0.030-0.047 | 0.735 | <.001 | 0.716-0.754 |
| East European | 0.356 | <.001 | 0.337-0.376 | 0.075 | 0.0656 | 0.065-0.084 | 0.028 | >.1 | 0.027-0.029 | 0.085 | >.1 | 0.076-0.093 | 0.659 | <.001 | 0.640-0.678 |
| French | 0.329 | <.001 | 0.309-0.349 | 0.064 | >.1 | 0.062-0.066 | 0.017 | <.001 | 0.011-0.023 | 0.04 | <.001 | 0.031-0.049 | 0.622 | <.001 | 0.601-0.642 |
| Germanic | 0.246 | <.001 | 0.227-0.266 | 0.059 | >.1 | 0.052-0.066 | 0.01 | <.001 | 0.005-0.015 | 0.023 | <.001 | 0.016-0.031 | 0.579 | <.001 | 0.557-0.601 |
| Hispanic | 0.472 | <.001 | 0.452-0.492 | 0.072 | >.1 | 0.065-0.079 | 0.036 | 0.0416 | 0.029-0.043 | 0.082 | >.1 | 0.076-0.087 | 0.727 | 0.0015 | 0.709-0.745 |
| Indian | 0.406 | >.1 | 0.394-0.417 | 0.07 | >.1 | 0.065-0.074 | 0.035 | 0.0888 | 0.029-0.041 | 0.104 | <.001 | 0.092-0.117 | 0.715 | 0.0729 | 0.698-0.733 |
| Italian | 0.509 | <.001 | 0.487-0.531 | 0.064 | >.1 | 0.062-0.066 | 0.044 | <.001 | 0.035-0.053 | 0.108 | <.001 | 0.095-0.122 | 0.808 | <.001 | 0.791-0.825 |
| Japanese | 0.848 | <.001 | 0.832-0.865 | 0.062 | >.1 | 0.058-0.066 | 0.051 | <.001 | 0.041-0.061 | 0.059 | 0.0012 | 0.048-0.069 | 0.908 | <.001 | 0.895-0.922 |
| Scandinavian | 0.261 | <.001 | 0.241-0.281 | 0.06 | >.1 | 0.053-0.066 | 0.011 | <.001 | 0.006-0.016 | 0.026 | <.001 | 0.018-0.033 | 0.585 | <.001 | 0.564-0.607 |

Over 55 Years

|  | Sensitivity | | | Specificity | | | Positive Predictive Value | | | Negative Predictive Value | | | Precision | | |
| --- | --- | --- | --- | --- | --- | --- | --- | --- | --- | --- | --- | --- | --- | --- | --- |
|  | Value | P | CI | Value | P | CI | Value | P | CI | Value | P | CI | Value | P | CI |
| African | 0.303 | <.001 | 0.264-0.343 | 0.965 | 0.1732 | 0.952-0.978 | 0.323 | <.001 | 0.283-0.363 | 0.961 | 0.8024 | 0.959-0.964 | 0.323 | <.001 | 0.283-0.363 |
| Anglo-American | 0.746 | <.001 | 0.709-0.784 | 0.829 | <.001 | 0.798-0.860 | 0.376 | <.001 | 0.335-0.417 | 0.959 | 0.5587 | 0.954-0.965 | 0.376 | <.001 | 0.335-0.417 |
| Arabic | 0.632 | <.001 | 0.591-0.673 | 0.948 | 0.6149 | 0.942-0.953 | 0.313 | <.001 | 0.274-0.353 | 0.985 | 0.0049 | 0.974-0.996 | 0.313 | <.001 | 0.274-0.353 |
| East Asian | 0.463 | 0.0019 | 0.421-0.505 | 0.993 | <.001 | 0.983-1.002 | 0.764 | <.001 | 0.728-0.801 | 0.974 | 0.2082 | 0.963-0.985 | 0.764 | <.001 | 0.728-0.801 |
| East European | 0.506 | 0.3743 | 0.486-0.526 | 0.947 | 0.5389 | 0.940-0.953 | 0.163 | <.001 | 0.130-0.195 | 0.989 | <.001 | 0.979-0.999 | 0.163 | <.001 | 0.130-0.195 |
| French | 0.321 | <.001 | 0.281-0.361 | 0.972 | 0.0283 | 0.957-0.987 | 0.49 | 0.4161 | 0.472-0.508 | 0.944 | 0.0081 | 0.925-0.964 | 0.49 | 0.4161 | 0.472-0.508 |
| Germanic | 0.249 | <.001 | 0.212-0.287 | 0.973 | 0.0182 | 0.958-0.988 | 0.522 | 0.0136 | 0.480-0.565 | 0.917 | <.001 | 0.894-0.940 | 0.522 | 0.0136 | 0.480-0.565 |
| Hispanic | 0.521 | 0.9098 | 0.518-0.524 | 0.955 | 0.8474 | 0.952-0.958 | 0.392 | <.001 | 0.351-0.434 | 0.973 | 0.2821 | 0.963-0.982 | 0.392 | <.001 | 0.351-0.434 |
| Indian | 0.607 | <.001 | 0.565-0.648 | 0.953 | 1 | 0.953-0.954 | 0.285 | <.001 | 0.246-0.324 | 0.987 | 0.0021 | 0.976-0.998 | 0.285 | <.001 | 0.246-0.324 |
| Italian | 0.778 | <.001 | 0.742-0.814 | 0.931 | 0.0104 | 0.909-0.953 | 0.538 | <.001 | 0.496-0.581 | 0.976 | 0.1365 | 0.963-0.988 | 0.538 | <.001 | 0.496-0.581 |
| Japanese | 0.896 | <.001 | 0.868-0.924 | 0.992 | <.001 | 0.983-1.001 | 0.952 | <.001 | 0.931-0.973 | 0.983 | 0.0136 | 0.971-0.995 | 0.952 | <.001 | 0.931-0.973 |
| Scandinavian | 0.267 | <.001 | 0.229-0.305 | 0.976 | 0.007 | 0.962-0.990 | 0.562 | <.001 | 0.520-0.604 | 0.921 | <.001 | 0.898-0.944 | 0.562 | <.001 | 0.520-0.604 |

|  | F1 | | | Prevalence | | | Detection Rate | | | Detection Prevalence | | | Balanced Accuracy | | |
| --- | --- | --- | --- | --- | --- | --- | --- | --- | --- | --- | --- | --- | --- | --- | --- |
|  | Value | P | CI | Value | P | CI | Value | P | CI | Value | P | CI | Value | P | CI |
| African | 0.303 | <.001 | 0.264-0.343 | 0.313 | <.001 | 0.273-0.353 | 0.051 | 0.0468 | 0.032-0.071 | 0.015 | 0.0021 | 0.003-0.027 | 0.048 | 0.0017 | 0.029-0.067 |
| Anglo-American | 0.746 | <.001 | 0.709-0.784 | 0.5 | 0.0526 | 0.458-0.542 | 0.121 | <.001 | 0.093-0.148 | 0.09 | <.001 | 0.066-0.114 | 0.24 | <.001 | 0.204-0.275 |
| Arabic | 0.632 | <.001 | 0.591-0.673 | 0.419 | 0.0314 | 0.377-0.461 | 0.036 | <.001 | 0.019-0.053 | 0.022 | 0.0302 | 0.009-0.036 | 0.072 | 0.3606 | 0.061-0.084 |
| East Asian | 0.463 | 0.0019 | 0.421-0.505 | 0.577 | <.001 | 0.535-0.619 | 0.046 | 0.0107 | 0.027-0.064 | 0.021 | 0.0189 | 0.008-0.034 | 0.028 | <.001 | 0.012-0.043 |
| East European | 0.506 | 0.3743 | 0.486-0.526 | 0.246 | <.001 | 0.209-0.283 | 0.019 | <.001 | 0.006-0.033 | 0.01 | <.001 | -1.22 | 0.061 | 0.0527 | 0.040-0.082 |
| French | 0.321 | <.001 | 0.281-0.361 | 0.388 | <.001 | 0.346-0.429 | 0.077 | 0.7313 | 0.072-0.081 | 0.024 | 0.0565 | 0.010-0.038 | 0.05 | 0.003 | 0.031-0.070 |
| Germanic | 0.249 | <.001 | 0.212-0.287 | 0.338 | <.001 | 0.297-0.378 | 0.104 | 0.0025 | 0.078-0.130 | 0.026 | 0.0803 | 0.011-0.040 | 0.049 | 0.0024 | 0.030-0.069 |
| Hispanic | 0.521 | 0.9098 | 0.518-0.524 | 0.448 | 0.5053 | 0.433-0.463 | 0.052 | 0.0497 | 0.032-0.071 | 0.027 | 0.1117 | 0.013-0.041 | 0.069 | 0.2192 | 0.053-0.084 |
| Indian | 0.607 | <.001 | 0.565-0.648 | 0.388 | <.001 | 0.346-0.429 | 0.029 | <.001 | 0.013-0.045 | 0.017 | 0.0051 | 0.005-0.030 | 0.062 | 0.0681 | 0.041-0.084 |
| Italian | 0.778 | <.001 | 0.742-0.814 | 0.637 | <.001 | 0.596-0.677 | 0.093 | 0.052 | 0.071-0.115 | 0.072 | <.001 | 0.050-0.094 | 0.134 | <.001 | 0.105-0.163 |
| Japanese | 0.896 | <.001 | 0.868-0.924 | 0.923 | <.001 | 0.898-0.948 | 0.14 | <.001 | 0.111-0.169 | 0.126 | <.001 | 0.098-0.153 | 0.132 | <.001 | 0.103-0.161 |
| Scandinavian | 0.267 | <.001 | 0.229-0.305 | 0.362 | <.001 | 0.321-0.403 | 0.102 | 0.0045 | 0.076-0.128 | 0.027 | 0.1169 | 0.013-0.041 | 0.048 | 0.0017 | 0.029-0.067 |

N2E

All

|  | Sensitivity | | | Specificity | | | Positive Predictive Value | | | Negative Predictive Value | | | Precision | | |
| --- | --- | --- | --- | --- | --- | --- | --- | --- | --- | --- | --- | --- | --- | --- | --- |
|  | Value | P | CI | Value | P | CI | Value | P | CI | Value | P | CI | Value | P | CI |
| African | 0.768 | <.001 | 0.754-0.782 | 0.951 | <.001 | 0.944-0.958 | 0.665 | <.001 | 0.649-0.680 | 0.97 | <.001 | 0.964-0.976 | 0.665 | <.001 | 0.649-0.680 |
| Anglo-American | 0.736 | <.001 | 0.722-0.750 | 0.935 | <.001 | 0.928-0.943 | 0.589 | <.001 | 0.573-0.605 | 0.966 | <.001 | 0.960-0.971 | 0.589 | <.001 | 0.573-0.605 |
| Arabic | 0.934 | <.001 | 0.925-0.943 | 0.953 | 0.001 | 0.946-0.960 | 0.714 | <.001 | 0.699-0.729 | 0.991 | <.001 | 0.988-0.994 | 0.714 | <.001 | 0.699-0.729 |
| East Asian | 0.942 | <.001 | 0.934-0.951 | 0.981 | <.001 | 0.976-0.986 | 0.865 | <.001 | 0.853-0.877 | 0.992 | <.001 | 0.989-0.996 | 0.865 | <.001 | 0.853-0.877 |
| European | 0.808 | 0.002 | 0.794-0.821 | 0.968 | 0.0246 | 0.963-0.974 | 0.764 | 0.0055 | 0.750-0.778 | 0.975 | >.1 | 0.973-0.978 | 0.764 | 0.0055 | 0.750-0.778 |
| Hispanic | 0.845 | <.001 | 0.833-0.857 | 0.982 | <.001 | 0.977-0.987 | 0.859 | <.001 | 0.847-0.871 | 0.98 | >.1 | 0.977-0.983 | 0.859 | <.001 | 0.847-0.871 |
| Scandinavian | 0.677 | <.001 | 0.662-0.692 | 0.987 | <.001 | 0.983-0.991 | 0.873 | <.001 | 0.861-0.884 | 0.96 | <.001 | 0.954-0.967 | 0.873 | <.001 | 0.861-0.884 |
| South Asian | 0.889 | <.001 | 0.878-0.900 | 0.938 | <.001 | 0.930-0.946 | 0.643 | <.001 | 0.627-0.658 | 0.985 | <.001 | 0.981-0.989 | 0.643 | <.001 | 0.627-0.658 |

|  | F1 | | | Prevalence | | | Detection Rate | | | Detection Prevalence | | | Balanced Accuracy | | |
| --- | --- | --- | --- | --- | --- | --- | --- | --- | --- | --- | --- | --- | --- | --- | --- |
|  | Value | P | CI | Value | P | CI | Value | P | CI | Value | P | CI | Value | P | CI |
| African | 0.712 | <.001 | 0.698-0.727 | 0.111 | >.1 | 0.111-0.111 | 0.085 | >.1 | 0.078-0.092 | 0.128 | >.1 | 0.124-0.132 | 0.859 | <.001 | 0.848-0.871 |
| Anglo-American | 0.655 | <.001 | 0.639-0.670 | 0.111 | >.1 | 0.111-0.111 | 0.081 | 0.0215 | 0.072-0.091 | 0.138 | 0.005 | 0.127-0.150 | 0.836 | <.001 | 0.824-0.848 |
| Arabic | 0.809 | <.001 | 0.796-0.822 | 0.111 | >.1 | 0.111-0.111 | 0.103 | 0.0044 | 0.093-0.113 | 0.145 | <.001 | 0.133-0.157 | 0.944 | <.001 | 0.935-0.952 |
| East Asian | 0.902 | <.001 | 0.892-0.913 | 0.111 | >.1 | 0.111-0.111 | 0.104 | 0.0022 | 0.094-0.114 | 0.121 | >.1 | 0.116-0.125 | 0.962 | <.001 | 0.955-0.969 |
| European | 0.785 | >.1 | 0.777-0.794 | 0.111 | >.1 | 0.111-0.111 | 0.089 | >.1 | 0.087-0.091 | 0.117 | >.1 | 0.108-0.126 | 0.888 | >.1 | 0.882-0.894 |
| Hispanic | 0.852 | <.001 | 0.840-0.864 | 0.111 | >.1 | 0.111-0.111 | 0.093 | >.1 | 0.091-0.096 | 0.109 | 0.0013 | 0.098-0.119 | 0.914 | <.001 | 0.904-0.923 |
| Scandinavian | 0.762 | 0.0111 | 0.748-0.777 | 0.111 | >.1 | 0.111-0.111 | 0.075 | <.001 | 0.066-0.084 | 0.086 | <.001 | 0.076-0.095 | 0.832 | <.001 | 0.820-0.844 |
| South Asian | 0.746 | <.001 | 0.732-0.760 | 0.111 | >.1 | 0.111-0.111 | 0.098 | 0.0991 | 0.090-0.106 | 0.153 | <.001 | 0.141-0.165 | 0.913 | <.001 | 0.904-0.923 |

Female

|  | Sensitivity | | | Specificity | | | Positive Predictive Value | | | Negative Predictive Value | | | Precision | | |
| --- | --- | --- | --- | --- | --- | --- | --- | --- | --- | --- | --- | --- | --- | --- | --- |
|  | Value | P | CI | Value | P | CI | Value | P | CI | Value | P | CI | Value | P | CI |
| African | 0.771 | <.001 | 0.746-0.795 | 0.949 | 0.0254 | 0.937-0.962 | 0.67 | <.001 | 0.642-0.697 | 0.969 | 0.0928 | 0.961-0.977 | 0.67 | <.001 | 0.642-0.697 |
| Anglo-American | 0.726 | <.001 | 0.698-0.754 | 0.919 | <.001 | 0.902-0.936 | 0.498 | <.001 | 0.467-0.529 | 0.968 | 0.0708 | 0.958-0.977 | 0.498 | <.001 | 0.467-0.529 |
| Arabic | 0.919 | <.001 | 0.895-0.942 | 0.967 | >.1 | 0.960-0.974 | 0.633 | <.001 | 0.594-0.673 | 0.994 | 0.0013 | 0.987-1.002 | 0.633 | <.001 | 0.594-0.673 |
| East Asian | 0.939 | <.001 | 0.924-0.953 | 0.978 | <.001 | 0.970-0.986 | 0.894 | <.001 | 0.877-0.912 | 0.987 | <.001 | 0.981-0.994 | 0.894 | <.001 | 0.877-0.912 |
| European | 0.742 | <.001 | 0.713-0.771 | 0.962 | >.1 | 0.961-0.963 | 0.656 | <.001 | 0.625-0.688 | 0.974 | >.1 | 0.972-0.976 | 0.656 | <.001 | 0.625-0.688 |
| Hispanic | 0.863 | <.001 | 0.845-0.882 | 0.978 | <.001 | 0.969-0.986 | 0.875 | <.001 | 0.857-0.894 | 0.976 | >.1 | 0.975-0.976 | 0.875 | <.001 | 0.857-0.894 |
| Scandinavian | 0.623 | <.001 | 0.594-0.652 | 0.988 | <.001 | 0.981-0.996 | 0.872 | <.001 | 0.851-0.894 | 0.955 | <.001 | 0.943-0.968 | 0.872 | <.001 | 0.851-0.894 |
| South Asian | 0.862 | <.001 | 0.841-0.884 | 0.946 | 0.0062 | 0.933-0.960 | 0.658 | <.001 | 0.629-0.687 | 0.983 | >.1 | 0.975-0.990 | 0.658 | <.001 | 0.629-0.687 |

|  | F1 | | | Prevalence | | | Detection Rate | | | Detection Prevalence | | | Balanced Accuracy | | |
| --- | --- | --- | --- | --- | --- | --- | --- | --- | --- | --- | --- | --- | --- | --- | --- |
|  | Value | P | CI | Value | P | CI | Value | P | CI | Value | P | CI | Value | P | CI |
| African | 0.717 | 0.002 | 0.690-0.743 | 0.116 | >.1 | 0.110-0.122 | 0.089 | >.1 | 0.089-0.090 | 0.134 | >.1 | 0.123-0.144 | 0.86 | 0.0059 | 0.840-0.880 |
| Anglo-American | 0.591 | <.001 | 0.560-0.622 | 0.099 | >.1 | 0.086-0.113 | 0.072 | 0.0272 | 0.055-0.089 | 0.145 | 0.0305 | 0.122-0.167 | 0.822 | <.001 | 0.799-0.846 |
| Arabic | 0.75 | >.1 | 0.748-0.752 | 0.057 | <.001 | 0.037-0.077 | 0.052 | <.001 | 0.033-0.071 | 0.083 | <.001 | 0.059-0.106 | 0.943 | <.001 | 0.923-0.963 |
| East Asian | 0.916 | <.001 | 0.900-0.932 | 0.164 | <.001 | 0.146-0.183 | 0.154 | <.001 | 0.136-0.172 | 0.172 | <.001 | 0.154-0.191 | 0.958 | <.001 | 0.946-0.970 |
| European | 0.696 | <.001 | 0.666-0.727 | 0.088 | 0.0147 | 0.068-0.107 | 0.065 | 0.0038 | 0.048-0.082 | 0.099 | 0.0113 | 0.079-0.120 | 0.852 | <.001 | 0.828-0.875 |
| Hispanic | 0.869 | <.001 | 0.850-0.888 | 0.15 | <.001 | 0.131-0.168 | 0.129 | <.001 | 0.112-0.147 | 0.147 | 0.0033 | 0.129-0.166 | 0.921 | <.001 | 0.906-0.935 |
| Scandinavian | 0.727 | 0.0344 | 0.700-0.754 | 0.108 | >.1 | 0.105-0.111 | 0.067 | 0.0035 | 0.051-0.083 | 0.077 | <.001 | 0.060-0.094 | 0.806 | <.001 | 0.782-0.829 |
| South Asian | 0.747 | >.1 | 0.741-0.752 | 0.106 | >.1 | 0.100-0.112 | 0.091 | >.1 | 0.090-0.093 | 0.139 | >.1 | 0.122-0.155 | 0.904 | 0.0168 | 0.886-0.923 |

Male

|  | Sensitivity | | | Specificity | | | Positive Predictive Value | | | Negative Predictive Value | | | Precision | | |
| --- | --- | --- | --- | --- | --- | --- | --- | --- | --- | --- | --- | --- | --- | --- | --- |
|  | Value | P | CI | Value | P | CI | Value | P | CI | Value | P | CI | Value | P | CI |
| African | 0.766 | <.001 | 0.750-0.783 | 0.952 | 0.002 | 0.943-0.960 | 0.662 | <.001 | 0.643-0.681 | 0.971 | 0.0036 | 0.964-0.977 | 0.662 | <.001 | 0.643-0.681 |
| Anglo-American | 0.74 | <.001 | 0.723-0.757 | 0.943 | <.001 | 0.934-0.952 | 0.633 | <.001 | 0.614-0.651 | 0.965 | <.001 | 0.957-0.972 | 0.633 | <.001 | 0.614-0.651 |
| Arabic | 0.937 | <.001 | 0.927-0.947 | 0.946 | <.001 | 0.938-0.954 | 0.731 | 0.0014 | 0.715-0.747 | 0.989 | <.001 | 0.985-0.993 | 0.731 | 0.0014 | 0.715-0.747 |
| East Asian | 0.945 | <.001 | 0.934-0.956 | 0.983 | <.001 | 0.976-0.989 | 0.842 | <.001 | 0.826-0.859 | 0.994 | <.001 | 0.990-0.998 | 0.842 | <.001 | 0.826-0.859 |
| European | 0.829 | >.1 | 0.826-0.832 | 0.971 | 0.0059 | 0.965-0.978 | 0.803 | <.001 | 0.787-0.818 | 0.976 | >.1 | 0.973-0.979 | 0.803 | <.001 | 0.787-0.818 |
| Hispanic | 0.832 | >.1 | 0.831-0.833 | 0.984 | <.001 | 0.978-0.990 | 0.847 | <.001 | 0.831-0.863 | 0.982 | >.1 | 0.978-0.987 | 0.847 | <.001 | 0.831-0.863 |
| Scandinavian | 0.7 | <.001 | 0.682-0.718 | 0.987 | <.001 | 0.982-0.992 | 0.873 | <.001 | 0.859-0.886 | 0.963 | <.001 | 0.955-0.970 | 0.873 | <.001 | 0.859-0.886 |
| South Asian | 0.9 | <.001 | 0.888-0.912 | 0.934 | <.001 | 0.924-0.944 | 0.637 | <.001 | 0.618-0.655 | 0.986 | 0.0022 | 0.981-0.991 | 0.637 | <.001 | 0.618-0.655 |

|  | F1 | | | Prevalence | | | Detection Rate | | | Detection Prevalence | | | Balanced Accuracy | | |
| --- | --- | --- | --- | --- | --- | --- | --- | --- | --- | --- | --- | --- | --- | --- | --- |
|  | Value | P | CI | Value | P | CI | Value | P | CI | Value | P | CI | Value | P | CI |
| African | 0.711 | <.001 | 0.692-0.729 | 0.108 | >.1 | 0.106-0.111 | 0.083 | 0.0918 | 0.073-0.093 | 0.125 | >.1 | 0.124-0.126 | 0.859 | <.001 | 0.845-0.873 |
| Anglo-American | 0.682 | <.001 | 0.664-0.700 | 0.116 | >.1 | 0.110-0.122 | 0.085 | >.1 | 0.078-0.093 | 0.135 | 0.0623 | 0.123-0.148 | 0.841 | <.001 | 0.827-0.855 |
| Arabic | 0.821 | <.001 | 0.807-0.836 | 0.134 | <.001 | 0.122-0.147 | 0.126 | <.001 | 0.114-0.138 | 0.173 | <.001 | 0.159-0.186 | 0.941 | <.001 | 0.932-0.951 |
| East Asian | 0.891 | <.001 | 0.876-0.905 | 0.087 | <.001 | 0.074-0.100 | 0.082 | 0.0981 | 0.071-0.093 | 0.097 | <.001 | 0.084-0.111 | 0.964 | <.001 | 0.955-0.973 |
| European | 0.815 | <.001 | 0.800-0.830 | 0.121 | 0.0589 | 0.109-0.132 | 0.1 | 0.0981 | 0.091-0.109 | 0.125 | >.1 | 0.124-0.125 | 0.9 | >.1 | 0.896-0.904 |
| Hispanic | 0.84 | <.001 | 0.824-0.856 | 0.093 | 0.0047 | 0.081-0.106 | 0.078 | 0.0118 | 0.066-0.089 | 0.092 | <.001 | 0.079-0.104 | 0.908 | 0.0568 | 0.895-0.921 |
| Scandinavian | 0.777 | >.1 | 0.767-0.787 | 0.112 | >.1 | 0.110-0.113 | 0.078 | 0.0085 | 0.067-0.089 | 0.09 | <.001 | 0.078-0.101 | 0.843 | <.001 | 0.829-0.858 |
| South Asian | 0.746 | <.001 | 0.729-0.763 | 0.113 | >.1 | 0.110-0.115 | 0.101 | 0.0587 | 0.090-0.113 | 0.159 | <.001 | 0.145-0.174 | 0.917 | <.001 | 0.906-0.928 |

Under 35 Years

|  | Sensitivity | | | Specificity | | | Positive Predictive Value | | | Negative Predictive Value | | | Precision | | |
| --- | --- | --- | --- | --- | --- | --- | --- | --- | --- | --- | --- | --- | --- | --- | --- |
|  | Value | P | CI | Value | P | CI | Value | P | CI | Value | P | CI | Value | P | CI |
| African | 0.777 | >.1 | 0.754-0.800 | 0.947 | 0.0207 | 0.928-0.966 | 0.56 | <.001 | 0.519-0.601 | 0.98 | >.1 | 0.978-0.981 | 0.56 | <.001 | 0.519-0.601 |
| Anglo-American | 0.636 | <.001 | 0.592-0.680 | 0.946 | 0.0321 | 0.927-0.966 | 0.443 | <.001 | 0.397-0.488 | 0.975 | >.1 | 0.970-0.979 | 0.443 | <.001 | 0.397-0.488 |
| Arabic | 0.931 | <.001 | 0.914-0.949 | 0.94 | <.001 | 0.926-0.954 | 0.743 | >.1 | 0.732-0.755 | 0.986 | 0.052 | 0.978-0.994 | 0.743 | >.1 | 0.732-0.755 |
| East Asian | 0.961 | <.001 | 0.944-0.977 | 0.983 | <.001 | 0.973-0.994 | 0.88 | <.001 | 0.856-0.905 | 0.995 | <.001 | 0.988-1.001 | 0.88 | <.001 | 0.856-0.905 |
| European | 0.825 | 0.0285 | 0.798-0.851 | 0.974 | 0.0639 | 0.963-0.986 | 0.813 | <.001 | 0.786-0.841 | 0.976 | >.1 | 0.974-0.979 | 0.813 | <.001 | 0.786-0.841 |
| Hispanic | 0.844 | <.001 | 0.821-0.867 | 0.983 | <.001 | 0.975-0.992 | 0.904 | <.001 | 0.884-0.924 | 0.972 | >.1 | 0.965-0.979 | 0.904 | <.001 | 0.884-0.924 |
| Scandinavian | 0.5 | <.001 | 0.458-0.541 | 0.989 | <.001 | 0.979-0.999 | 0.801 | <.001 | 0.767-0.835 | 0.96 | <.001 | 0.943-0.976 | 0.801 | <.001 | 0.767-0.835 |
| South Asian | 0.904 | <.001 | 0.884-0.925 | 0.943 | <.001 | 0.928-0.958 | 0.722 | >.1 | 0.709-0.735 | 0.983 | >.1 | 0.978-0.989 | 0.722 | >.1 | 0.709-0.735 |

|  | F1 | | | Prevalence | | | Detection Rate | | | Detection Prevalence | | | Balanced Accuracy | | |
| --- | --- | --- | --- | --- | --- | --- | --- | --- | --- | --- | --- | --- | --- | --- | --- |
|  | Value | P | CI | Value | P | CI | Value | P | CI | Value | P | CI | Value | P | CI |
| African | 0.651 | <.001 | 0.612-0.690 | 0.079 | 0.0058 | 0.056-0.102 | 0.061 | 0.004 | 0.040-0.082 | 0.109 | >.1 | 0.092-0.127 | 0.862 | >.1 | 0.841-0.883 |
| Anglo-American | 0.522 | <.001 | 0.476-0.568 | 0.062 | <.001 | 0.038-0.085 | 0.039 | <.001 | 0.020-0.059 | 0.089 | 0.0105 | 0.062-0.116 | 0.791 | <.001 | 0.754-0.829 |
| Arabic | 0.827 | <.001 | 0.803-0.851 | 0.156 | <.001 | 0.134-0.178 | 0.146 | <.001 | 0.124-0.167 | 0.196 | <.001 | 0.172-0.220 | 0.936 | <.001 | 0.919-0.952 |
| East Asian | 0.919 | <.001 | 0.897-0.940 | 0.11 | >.1 | 0.108-0.111 | 0.105 | >.1 | 0.091-0.120 | 0.12 | >.1 | 0.114-0.125 | 0.972 | <.001 | 0.958-0.986 |
| European | 0.819 | <.001 | 0.792-0.846 | 0.117 | >.1 | 0.110-0.123 | 0.096 | >.1 | 0.092-0.101 | 0.118 | >.1 | 0.111-0.125 | 0.9 | 0.0585 | 0.878-0.921 |
| Hispanic | 0.873 | <.001 | 0.851-0.895 | 0.152 | <.001 | 0.129-0.174 | 0.128 | <.001 | 0.107-0.148 | 0.141 | 0.0679 | 0.122-0.161 | 0.914 | <.001 | 0.896-0.932 |
| Scandinavian | 0.615 | <.001 | 0.575-0.656 | 0.076 | 0.0031 | 0.052-0.099 | 0.038 | <.001 | 0.020-0.055 | 0.047 | <.001 | 0.027-0.067 | 0.744 | <.001 | 0.708-0.781 |
| South Asian | 0.803 | <.001 | 0.777-0.829 | 0.14 | 0.0017 | 0.118-0.162 | 0.126 | <.001 | 0.105-0.148 | 0.175 | <.001 | 0.151-0.199 | 0.924 | <.001 | 0.906-0.942 |

35-55 Years

|  | Sensitivity | | | Specificity | | | Positive Predictive Value | | | Negative Predictive Value | | | Precision | | |
| --- | --- | --- | --- | --- | --- | --- | --- | --- | --- | --- | --- | --- | --- | --- | --- |
|  | Value | P | CI | Value | P | CI | Value | P | CI | Value | P | CI | Value | P | CI |
| African | 0.777 | <.001 | 0.761-0.794 | 0.953 | 0.0173 | 0.944-0.961 | 0.705 | <.001 | 0.687-0.723 | 0.967 | <.001 | 0.960-0.974 | 0.705 | <.001 | 0.687-0.723 |
| Anglo-American | 0.694 | <.001 | 0.673-0.716 | 0.938 | <.001 | 0.926-0.949 | 0.528 | <.001 | 0.505-0.551 | 0.968 | <.001 | 0.960-0.976 | 0.528 | <.001 | 0.505-0.551 |
| Arabic | 0.937 | <.001 | 0.926-0.949 | 0.954 | 0.0742 | 0.947-0.962 | 0.722 | 0.0559 | 0.704-0.741 | 0.991 | <.001 | 0.987-0.996 | 0.722 | 0.0559 | 0.704-0.741 |
| East Asian | 0.943 | <.001 | 0.932-0.954 | 0.98 | <.001 | 0.973-0.986 | 0.859 | <.001 | 0.844-0.875 | 0.992 | <.001 | 0.988-0.996 | 0.859 | <.001 | 0.844-0.875 |
| European | 0.823 | >.1 | 0.823-0.824 | 0.968 | 0.0865 | 0.960-0.975 | 0.767 | <.001 | 0.748-0.785 | 0.977 | >.1 | 0.975-0.978 | 0.767 | <.001 | 0.748-0.785 |
| Hispanic | 0.847 | 0.0015 | 0.831-0.863 | 0.982 | <.001 | 0.976-0.988 | 0.852 | <.001 | 0.836-0.868 | 0.981 | >.1 | 0.978-0.985 | 0.852 | <.001 | 0.836-0.868 |
| Scandinavian | 0.671 | <.001 | 0.650-0.692 | 0.987 | <.001 | 0.981-0.993 | 0.854 | <.001 | 0.837-0.870 | 0.964 | <.001 | 0.956-0.972 | 0.854 | <.001 | 0.837-0.870 |
| South Asian | 0.89 | <.001 | 0.877-0.904 | 0.928 | <.001 | 0.918-0.939 | 0.621 | <.001 | 0.600-0.641 | 0.984 | 0.0257 | 0.979-0.990 | 0.621 | <.001 | 0.600-0.641 |

|  | F1 | | | Prevalence | | | Detection Rate | | | Detection Prevalence | | | Balanced Accuracy | | |
| --- | --- | --- | --- | --- | --- | --- | --- | --- | --- | --- | --- | --- | --- | --- | --- |
|  | Value | P | CI | Value | P | CI | Value | P | CI | Value | P | CI | Value | P | CI |
| African | 0.739 | <.001 | 0.722-0.757 | 0.125 | 0.0045 | 0.112-0.139 | 0.097 | >.1 | 0.089-0.105 | 0.138 | 0.0225 | 0.124-0.152 | 0.865 | <.001 | 0.851-0.879 |
| Anglo-American | 0.6 | <.001 | 0.577-0.623 | 0.09 | 0.0042 | 0.077-0.104 | 0.063 | <.001 | 0.051-0.074 | 0.119 | >.1 | 0.112-0.125 | 0.816 | <.001 | 0.798-0.834 |
| Arabic | 0.816 | <.001 | 0.799-0.833 | 0.111 | >.1 | 0.109-0.113 | 0.104 | 0.0145 | 0.091-0.117 | 0.144 | 0.0022 | 0.129-0.159 | 0.946 | <.001 | 0.936-0.956 |
| East Asian | 0.899 | <.001 | 0.886-0.913 | 0.115 | >.1 | 0.108-0.121 | 0.108 | 0.001 | 0.095-0.121 | 0.126 | >.1 | 0.124-0.127 | 0.961 | <.001 | 0.952-0.970 |
| European | 0.794 | 0.0074 | 0.777-0.811 | 0.113 | >.1 | 0.109-0.117 | 0.093 | >.1 | 0.090-0.096 | 0.121 | >.1 | 0.117-0.125 | 0.895 | >.1 | 0.892-0.899 |
| Hispanic | 0.849 | <.001 | 0.833-0.865 | 0.106 | >.1 | 0.101-0.110 | 0.089 | >.1 | 0.088-0.091 | 0.105 | 0.0025 | 0.091-0.119 | 0.914 | <.001 | 0.902-0.927 |
| Scandinavian | 0.751 | 0.0117 | 0.732-0.771 | 0.099 | >.1 | 0.087-0.111 | 0.066 | <.001 | 0.055-0.078 | 0.078 | <.001 | 0.065-0.090 | 0.829 | <.001 | 0.812-0.846 |
| South Asian | 0.731 | <.001 | 0.713-0.750 | 0.115 | >.1 | 0.108-0.122 | 0.103 | 0.0254 | 0.090-0.115 | 0.165 | <.001 | 0.150-0.181 | 0.909 | 0.003 | 0.897-0.922 |

Over 55 Years

|  | Sensitivity | | | Specificity | | | Positive Predictive Value | | | Negative Predictive Value | | | Precision | | |
| --- | --- | --- | --- | --- | --- | --- | --- | --- | --- | --- | --- | --- | --- | --- | --- |
|  | Value | P | CI | Value | P | CI | Value | P | CI | Value | P | CI | Value | P | CI |
| African | 0.719 | <.001 | 0.683-0.756 | 0.951 | >.1 | 0.938-0.964 | 0.626 | <.001 | 0.587-0.666 | 0.967 | >.1 | 0.961-0.974 | 0.626 | <.001 | 0.587-0.666 |
| Anglo-American | 0.821 | >.1 | 0.818-0.823 | 0.911 | <.001 | 0.895-0.926 | 0.742 | >.1 | 0.725-0.759 | 0.942 | <.001 | 0.929-0.954 | 0.742 | >.1 | 0.725-0.759 |
| Arabic | 0.924 | <.001 | 0.893-0.954 | 0.962 | >.1 | 0.961-0.963 | 0.585 | <.001 | 0.531-0.639 | 0.995 | 0.0095 | 0.985-1.005 | 0.585 | <.001 | 0.531-0.639 |
| East Asian | 0.914 | <.001 | 0.889-0.939 | 0.984 | 0.0025 | 0.973-0.996 | 0.867 | <.001 | 0.837-0.896 | 0.99 | 0.0062 | 0.981-1.000 | 0.867 | <.001 | 0.837-0.896 |
| European | 0.721 | <.001 | 0.684-0.758 | 0.964 | >.1 | 0.962-0.966 | 0.685 | 0.0121 | 0.646-0.724 | 0.97 | >.1 | 0.965-0.974 | 0.685 | 0.0121 | 0.646-0.724 |
| Hispanic | 0.84 | >.1 | 0.816-0.865 | 0.981 | 0.0219 | 0.967-0.995 | 0.795 | <.001 | 0.757-0.833 | 0.986 | 0.0729 | 0.974-0.998 | 0.795 | <.001 | 0.757-0.833 |
| Scandinavian | 0.773 | <.001 | 0.747-0.799 | 0.986 | <.001 | 0.977-0.994 | 0.929 | <.001 | 0.910-0.949 | 0.948 | <.001 | 0.934-0.961 | 0.929 | <.001 | 0.910-0.949 |
| South Asian | 0.837 | >.1 | 0.816-0.857 | 0.962 | >.1 | 0.961-0.963 | 0.587 | <.001 | 0.536-0.638 | 0.989 | 0.0544 | 0.976-1.001 | 0.587 | <.001 | 0.536-0.638 |

|  | F1 | | | Prevalence | | | Detection Rate | | | Detection Prevalence | | | Balanced Accuracy | | |
| --- | --- | --- | --- | --- | --- | --- | --- | --- | --- | --- | --- | --- | --- | --- | --- |
|  | Value | P | CI | Value | P | CI | Value | P | CI | Value | P | CI | Value | P | CI |
| African | 0.669 | <.001 | 0.631-0.708 | 0.101 | >.1 | 0.085-0.116 | 0.072 | 0.0655 | 0.050-0.095 | 0.116 | >.1 | 0.106-0.126 | 0.835 | <.001 | 0.805-0.865 |
| Anglo-American | 0.779 | >.1 | 0.761-0.797 | 0.237 | <.001 | 0.214-0.261 | 0.195 | <.001 | 0.173-0.216 | 0.263 | <.001 | 0.239-0.287 | 0.866 | 0.0023 | 0.846-0.885 |
| Arabic | 0.716 | 0.0224 | 0.667-0.766 | 0.054 | <.001 | 0.028-0.081 | 0.05 | 0.0035 | 0.025-0.075 | 0.086 | 0.019 | 0.054-0.118 | 0.943 | <.001 | 0.916-0.969 |
| East Asian | 0.89 | <.001 | 0.862-0.917 | 0.098 | >.1 | 0.080-0.117 | 0.09 | >.1 | 0.087-0.093 | 0.104 | 0.095 | 0.080-0.127 | 0.949 | <.001 | 0.929-0.969 |
| European | 0.703 | <.001 | 0.665-0.741 | 0.096 | >.1 | 0.075-0.117 | 0.069 | 0.0358 | 0.047-0.091 | 0.101 | 0.0638 | 0.075-0.127 | 0.843 | <.001 | 0.812-0.873 |
| Hispanic | 0.817 | 0.0024 | 0.780-0.853 | 0.078 | 0.0056 | 0.052-0.103 | 0.065 | 0.0253 | 0.041-0.089 | 0.082 | 0.002 | 0.056-0.108 | 0.911 | >.1 | 0.888-0.934 |
| Scandinavian | 0.844 | <.001 | 0.820-0.868 | 0.192 | <.001 | 0.168-0.215 | 0.148 | <.001 | 0.127-0.170 | 0.159 | <.001 | 0.137-0.182 | 0.879 | >.1 | 0.867-0.892 |
| South Asian | 0.69 | <.001 | 0.642-0.738 | 0.06 | <.001 | 0.034-0.086 | 0.05 | 0.0021 | 0.026-0.074 | 0.086 | 0.0129 | 0.055-0.116 | 0.899 | >.1 | 0.889-0.909 |

Ethnicity Estimator

All

|  | Sensitivity | | |
| --- | --- | --- | --- |
|  | Value | P | CI |
| British | 0.6792 | <.001 | 0.666-0.692 |
| Irish | 0.2109 | <.001 | 0.199-0.222 |
| Other White | 0.4939 | >.1 | 0.486-0.502 |
| Bangladeshi | 0.5817 | <.001 | 0.568-0.595 |
| Chinese | 0.9494 | <.001 | 0.942-0.957 |
| Indian | 0.6764 | <.001 | 0.663-0.689 |
| Pakistani | 0.7968 | <.001 | 0.785-0.808 |
| Other Asian | 0.281 | <.001 | 0.268-0.294 |
| African | 0.3682 | <.001 | 0.355-0.382 |
| Caribbean | 0.1635 | <.001 | 0.153-0.174 |
| Other | 0.1517 | <.001 | 0.141-0.162 |

Female

|  | Sensitivity | | |
| --- | --- | --- | --- |
|  | Value | P | CI |
| British | 0.6725 | <.001 | 0.649-0.696 |
| Irish | 0.1728 | <.001 | 0.153-0.192 |
| Other White | 0.4505 | 0.0031 | 0.425-0.476 |
| Bangladeshi | 0.5982 | <.001 | 0.573-0.623 |
| Chinese | 0.9237 | <.001 | 0.909-0.938 |
| Indian | 0.7012 | <.001 | 0.678-0.724 |
| Pakistani | 0.8333 | <.001 | 0.814-0.853 |
| Other Asian | 0.2693 | <.001 | 0.247-0.292 |
| African | 0.3914 | <.001 | 0.367-0.416 |
| Caribbean | 0.1542 | <.001 | 0.135-0.173 |
| Other | 0.1249 | <.001 | 0.108-0.142 |

Male

|  | Sensitivity | | |
| --- | --- | --- | --- |
|  | Value | P | CI |
| British | 0.6822 | <.001 | 0.659-0.706 |
| Irish | 0.2128 | <.001 | 0.192-0.234 |
| Other White | 0.5058 | >.1 | 0.488-0.523 |
| Bangladeshi | 0.5814 | <.001 | 0.556-0.606 |
| Chinese | 0.9828 | <.001 | 0.974-0.991 |
| Indian | 0.6831 | <.001 | 0.66-0.707 |
| Pakistani | 0.8062 | <.001 | 0.786-0.826 |
| Other Asian | 0.2753 | <.001 | 0.253-0.298 |
| African | 0.3879 | <.001 | 0.363-0.413 |
| Caribbean | 0.1589 | <.001 | 0.14-0.178 |
| Other | 0.1572 | <.001 | 0.138-0.176 |

Under 35 Years

|  | Sensitivity | | |
| --- | --- | --- | --- |
|  | Value | P | CI |
| British | 0.5953 | <.001 | 0.564-0.627 |
| Irish | 0.1974 | <.001 | 0.172-0.223 |
| Other White | 0.5163 | 0.0263 | 0.485-0.548 |
| Bangladeshi | 0.5262 | <.001 | 0.494-0.558 |
| Chinese | 0.9556 | <.001 | 0.941-0.97 |
| Indian | 0.6407 | <.001 | 0.61-0.671 |
| Pakistani | 0.8302 | <.001 | 0.806-0.854 |
| Other Asian | 0.2241 | <.001 | 0.197-0.251 |
| African | 0.3169 | <.001 | 0.287-0.347 |
| Caribbean | 0.1718 | <.001 | 0.147-0.196 |
| Other | 0.153 | <.001 | 0.13-0.176 |

35-55 Years

|  | Sensitivity | | |
| --- | --- | --- | --- |
|  | Value | P | CI |
| British | 0.6397 | <.001 | 0.609-0.67 |
| Irish | 0.2152 | <.001 | 0.189-0.242 |
| Other White | 0.5222 | 0.0095 | 0.49-0.554 |
| Bangladeshi | 0.6091 | <.001 | 0.578-0.64 |
| Chinese | 0.9457 | <.001 | 0.93-0.961 |
| Indian | 0.6851 | <.001 | 0.655-0.715 |
| Pakistani | 0.8016 | <.001 | 0.776-0.827 |
| Other Asian | 0.2942 | <.001 | 0.265-0.323 |
| African | 0.3761 | <.001 | 0.345-0.407 |
| Caribbean | 0.1688 | <.001 | 0.145-0.193 |
| Other | 0.1579 | <.001 | 0.134-0.182 |

Over 55 Years

|  | Sensitivity | | |
| --- | --- | --- | --- |
|  | Value | P | CI |
| British | 0.768 | <.001 | 0.741-0.795 |
| Irish | 0.228 | <.001 | 0.201-0.255 |
| Other White | 0.3712 | <.001 | 0.34-0.402 |
| Bangladeshi | 0.6328 | <.001 | 0.602-0.664 |
| Chinese | 0.9269 | <.001 | 0.909-0.944 |
| Indian | 0.6989 | <.001 | 0.67-0.728 |
| Pakistani | 0.773 | <.001 | 0.746-0.8 |
| Other Asian | 0.2942 | <.001 | 0.265-0.323 |
| African | 0.2774 | <.001 | 0.249-0.306 |
| Caribbean | 0.1728 | <.001 | 0.148-0.197 |
| Other | 0.1333 | <.001 | 0.111-0.155 |

Deviation of Sensitivities

All

|  | Average Deviation | P | CI |
| --- | --- | --- | --- |
| Ethnicity Estimator | 0.2287 | <.001 | 0.217-0.24 |
| Ethnicolr | 0.1617 | <.001 | 0.147-0.176 |
| NamePrism | 0.159 | <.001 | 0.145-0.173 |
| N2E | 0.0776 | <.001 | 0.066-0.09 |

Female

|  | Average Deviation | P | CI |
| --- | --- | --- | --- |
| Ethnicity Estimator | 0.2406 | <.001 | 0.219-0.262 |
| Ethnicolr | 0.1557 | <.001 | 0.133-0.179 |
| NamePrism | 0.1931 | <.001 | 0.167-0.219 |
| N2E | 0.0901 | <.001 | 0.067-0.114 |

Male

|  | Average Deviation | P | CI |
| --- | --- | --- | --- |
| Ethnicity Estimator | 0.2323 | <.001 | 0.213-0.253 |
| Ethnicolr | 0.171 | <.001 | 0.156-0.186 |
| NamePrism | 0.1467 | <.001 | 0.131-0.163 |
| N2E | 0.0724 | <.001 | 0.062-0.083 |

Under 35 Years

|  | Average Deviation | P | CI |
| --- | --- | --- | --- |
| Ethnicity Estimator | 0.2305 | <.001 | 0.204-0.257 |
| Ethnicolr | 0.1731 | <.001 | 0.138-0.208 |
| NamePrism | 0.1732 | <.001 | 0.145-0.203 |
| N2E | 0.1197 | <.001 | 0.094-0.146 |

35-55 Years

|  | Average Deviation | P | CI |
| --- | --- | --- | --- |
| Ethnicity Estimator | 0.2272 | <.001 | 0.2-0.254 |
| Ethnicolr | 0.1547 | <.001 | 0.135-0.174 |
| NamePrism | 0.1602 | <.001 | 0.142-0.178 |
| N2E | 0.0816 | <.001 | 0.068-0.096 |

Over 55 Years

|  | Average Deviation | P | CI |
| --- | --- | --- | --- |
| Ethnicity Estimator | 0.2548 | <.001 | 0.228-0.281 |
| Ethnicolr | 0.1747 | <.001 | 0.144-0.208 |
| NamePrism | 0.1407 | <.001 | 0.112-0.17 |
| N2E | 0.0607 | <.001 | 0.037-0.086 |
